# Fat Metabolism Regulates Satiety Behavior in *C. elegans*

**DOI:** 10.1038/srep24841

**Published:** 2016-04-21

**Authors:** Moonjung Hyun, Kristen Davis, Inhwan Lee, Jeongho Kim, Catherine Dumur, Young-Jai You

**Affiliations:** 1 Department of Biochemistry and Molecular Biology, Virginia Commonwealth University, Richmond, Virginia, USA; 2 Department of Biological Sciences, Inha University, Incheon, 402-751, South Korea; 3 Department of Pathology, Virginia Commonwealth University, Richmond, VA 23298, USA.

## Abstract

Animals change feeding behavior depending on their metabolic status; starved animals are eager to eat and satiated animals stop eating. *C. elegans* exhibits satiety quiescence under certain conditions that mimics many aspects of post-prandial sleep in mammals. Here we show that this feeding behavior depends on fat metabolism mediated by the SREBP-SCD pathway, an acetyl-CoA carboxylase (ACC) and certain nuclear hormone receptors (NRs). Mutations of the genes in the SREBP-SCD pathway reduce satiety quiescence. An RNA interference (RNAi) screen of the genes that regulate glucose and fatty acid metabolism identified an ACC necessary for satiety quiescence in *C. elegans*. ACC catalyzes the first step in *de novo* fatty acid biosynthesis known to be downstream of the SREBP pathway in mammals. We identified 28 NRs by microarray whose expression changes during refeeding after being starved. When individually knocked down by RNAi, 11 NRs among 28 affect both fat storage and satiety behavior. Our results show that the major fat metabolism pathway regulates feeding behavior and NRs could be the mediators to link the feeding behavior to the metabolic changes.

Obesity and overweight pose a major risk for chronic diseases, including Type 2 Diabetes, cardiovascular disease, hypertension, stroke, and certain forms of cancer^1^. One of the main causes of the pandemic of obesity and overweight is misregulation of feeding. Feeding is controlled by physiological, environmental and genetic factors. Correctly assessing nutritional status is critical to maintain energy balance and body weight, since many feeding control signals are directly regulated by the nutritional status^2^.

Patients deficient in leptin fail to control feeding and as a result become severely obese, indicating that a single gene mutation is sufficient to affect feeding behavior^3^,^4^. Leptin is produced in adipose tissue and signals to the brain to reduce feeding, indicating that communication between the fat storage and the brain is critical for proper regulation of feeding. This communication between the fat storage and the brain is likely conserved in other animals as well because almost all animals store their surplus energy in fat and because a mechanism to assess the amount of energy stored would be critical to feeding behavior and development (e.g. reproduction). Leptin only exists in vertebrates, however, raising the question of how the brain senses fat storage to control feeding and other developmental processes in invertebrate animals. This suggests ‘fat to brain’ communications that are evolutionarily conserved but as yet to be discovered.

*C. elegans* has been a useful genetic model system to study metabolism; although it lacks a designated fat tissue, it stores fat in the form of triglycerides in the intestine or in the hypodermis. Storing fat in the visceral tissue (intestine) or the subcutaneous layer (hypodermis) other than a designated fat tissue is common in many animals including humans and considered as an ancient way of storing fat before a designated fat tissue evolved. In addition, *C. elegans* has highly conserved genes that regulate fat metabolism, such as sterol response element binding protein (SREBP)^5^,^6^,^7^. Moreover, it exhibits conserved behavioral responses to starvation and satiety^8^,^9^, making it a suitable genetic model system to study how fat metabolism regulates feeding behavior.

Nuclear hormone receptors (NRs) are a family of transcription factors, many of which regulate metabolism in response to changes of nutritional inputs. For instance, peroxisome proliferator activated receptor α (PPARα) is a master regulator of gene expression in response to fasting^10^. PPARγ regulates fatty acid synthesis to regulate adipogenesis to store fat under good nutrition^11^. However, most of the NRs still remain orphans with unknown ligands, implying that we do not fully appreciate their significance in metabolism. *C. elegans* has about 7 times as many NRs (293, called NHR) as mammals (40), signifying the importance of this gene family and suggesting the potential use of *C. elegans* as a powerful genetic model to study the diversified roles of NRs. The functions of most of them are unknown but among several known NHRs are ones involved in dauer formation (DAF-12)^12^,^13^ and molting (NHR-23 and NHR-25)^14^,^15^, which are essential for critical development decisions or processes based on metabolism. Dauer is a dormant stage in worms that survives unfavorable environmental conditions such as starvation. Since *C. elegans* becomes a dauer when it lacks nutrient signals such as insulin^16^, the dauer decision is tightly linked to an animal’s metabolic status. Molting is also tightly linked to metabolism; malnourished *C. elegans* grows slowly and delays molting (Young-Jai You, unpublished data). Furthermore, Watson and others showed that NHRs regulate the transcriptional network in order to respond to dietary differences and to coordinate appropriate metabolic changes^17^. These studies show that *C. elegans* NHRs serve conserved functions to regulate metabolism in response to changes of nutritional input, like those of mammals. However, whether a NR regulates feeding behavior relevant to metabolic changes has not been tested in *C. elegans* or mammals.

In this study, we report that fat metabolism regulates satiety quiescence in *C. elegans*; addition of external fat promotes satiety quiescence and deficiency of fat reduces satiety quiescence. The canonical SREBP-SCD pathway that regulates *de novo* fatty acid synthesis is necessary in worms to promote satiety quiescence. From an RNAi screen of the genes that regulate glucose and fatty acid metabolisms, we identified *pod-2*, which encodes an acetyl-CoA carboxylase to catalyze the first step in *de novo* fatty acid biosynthesis downstream of SREBP. Finally, we identified a group of nuclear hormone receptors whose expression changes by starvation and refeeding; when knocked down these NHRs affect both satiety quiescence and fat storage. Our results show that fat metabolism regulates feeding through SREBP and that certain NHRs in *C. elegans* potentially link the metabolic status to feeding behavior.

## Results

### Mutants of SREBP and SCDs are defective in satiety quiescence

**Figure 1:**
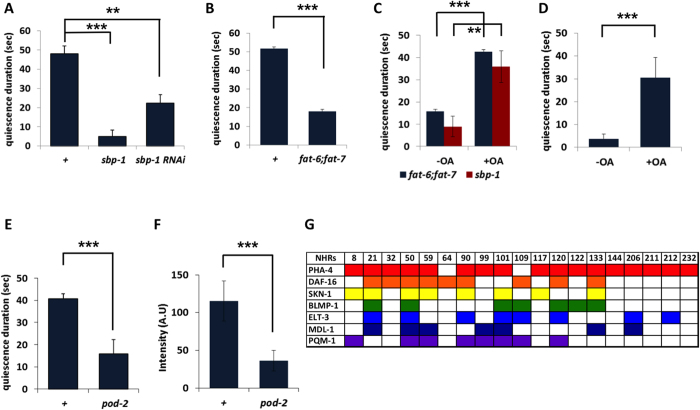
Fat is necessary for satiety quiescence. (**A**–**D**) Satiety quiescence depends on fat storage. (**A**) RNAi of *sbp-1* or a reduced function mutation of *sbp-1* reduces satiety quiescence. (**B**) *fat-6; fat-7* double mutants show reduced quiescence compared to wild type (+) after 12 hrs of fasting and 6 hrs of subsequent refeeding. (**C**) 6 hrs of refeeding on oleic acid (OA, 600 μM) restores normal quiescence to *fat-6; fat-7* double mutants and to reduced-function mutants of *sbp-1*. (**D**) Under conditions where wild type worms are rarely quiescent, oleic acid (OA, 600 μM) induces significant quiescence (see Materials and Methods). For satiety quiescence test, 10 mutants and 10 con-current wild-type animals were tested. The experiments were independently repeated three times. **p < *0.01*, ***p < *0.001* by Student’s *t* test. (**E**,**F**) Acetyl-CoA carboxylase (ACC) regulates fat storage and satiety quiescence. (**E**) *pod-2* mutants have reduced satiety quiescence. 10 mutants and 10 con-current wild-type animals were tested. The experiments were independently repeated three times. ***p < *0.001* by Student’s *t* test. (**F**) *pod-2* mutants have reduced fat storage. More than 20 mutants and 20 wild type animals were tested. The experiments were independently repeated three times. ***p < *0.001* by Student’s *t* test. (**G**) Seven transcription factors have their binding sites on the putative promoters of 19 NHRs among the 28 NHRs. The transcription factors are color-coded and the binding sites are shown for each NHR gene.

Previous studies have shown that *C. elegans* has a conserved pathway for fatty acid synthesis. Sterol regulatory element-binding protein-1C (SREBP-1C) is the major transcription factor to regulate the expression of genes for fatty acids synthesis^18^,^19^,^20^,^21^. Loss-of-function mutants of *sbp-1*, the *C. elegans* homolog of SREBP-1C, are arrested at the first stage of larval development (L1) and fail to grow^7^,^22^. *C. elegans* SREBP pathway also consists of conserved downstream targets such as stearoyl-CoA desaturase (SCD)^7^,^23^. To examine the role of the SREBP pathway on feeding behavior, we performed RNAi on components of this pathway and measured changes in satiety quiescence. RNA interference (RNAi) or a reduced function mutation in *sbp-1* both significantly reduces satiety quiescence (Fig. 1A). *C. elegans* has three SCD genes, *fat-5*, *fat-6* and *fat-7* that function downstream of *sbp-1* to produce unsaturated fatty acids; individual mutants do not show particular phenotypes. However, triple mutants of *fat-5; fat-6; fat-7* are larval lethal^22^, phenocopying *sbp-1* mutants. A double mutant of *fat-6* and *fat-7* are viable yet grow slow and store less fat than wild type^22^,^23^. To test whether the defect of *sbp-1* mutants in quiescence is due to altered fat metabolism mediated by the SCDs, we examined satiety quiescence of *fat-6; fat-7* mutants. The double mutants are also defective in satiety quiescence (Fig. 1B), suggesting intact fat metabolism is necessary for satiety quiescence. The defects in satiety quiescence in *sbp-1* or *fat-6; fat-7* mutants were completely rescued by growing them with the addition of an exogenous fat source, oleic acid (600 μM) (Fig. 1C). It has been shown that oleic acid supplement (600 μM) rescued several fat-deficient mutants including *sbp-1* by restoring their fat storage^22^,^23^. Moreover, addition of exogenous oleic acid could promote satiety quiescence under the conditions where we normally do not observe strong quiescence (Fig. 1D, Materials and Methods). These results show that fat synthesis mediated by the canonical SREBP-SCD pathway is necessary for worms to show satiety quiescence.

### *pod-2*, a *de novo* fatty acid synthase is necessary for satiety quiescence

To further investigate how metabolism affects satiety behavior, we performed an RNAi screen of selected metabolic genes that regulate mostly fat and carbohydrate metabolisms^24^. Among 143 of the tested genes (Supplemental Table S1), *pod-2* was identified to regulate satiety quiescence from the primary screen. A temperature sensitive *pod-2* mutant showed reduced satiety quiescence at a non-permissive temperature, validating the RNAi screen result (Fig. 1E). *pod-2* encodes a homolog of acetyl-CoA carboxylase (ACC) which is essential in embryo development and required for osmotic protection of the egg shell^25^,^26^. From sequence homology, POD-2 is predicted to catalyze the first step in *de novo* fatty acid biosynthesis. Indeed, the *pod-2* mutants have reduced fat storage compared to wild type (Fig. 1F), suggesting the reduced fat storage that resulted from reduced fatty acid synthesis could be a cause of reduced satiety quiescence. Together with the defect in satiety quiescence of the mutants in the SREBP pathway, these results suggest that fatty acid metabolism plays a role in satiety quiescence.

### Starvation and refeeding changes expression of a group of nuclear hormone receptor genes

We have observed that satiety quiescence is consistent when worms are refed after starvation. To find other genes that link metabolic changes to satiety quiescence, we profiled the gene expression changes during starvation and refeeding. These profiles would reveal the genes whose expression changes depend on metabolic status and thus provide potential targets that link metabolism and feeding behavior. For example, genes that encode for neuropeptides that regulate feeding behavior have expression levels dependent on the metabolic state.

The microarrays were performed on samples from animals that were either well-fed, starved, or refed after starvation. We collected triplicates of each sample from 5 time points of starvation and refeeding: (1) well-fed (WF: as control, before starvation), (2) 12 hour-starved (12S), (3) 12 hour-starved and 1 hr-refed (12S1RF), (4) 12 hour-starved and 2 hr-refed (12S2RF), (5) 12 hour-starved and 3 hr-refed (12S3RF) (see Materials and Methods). We flanked the starvation time point with a well-fed time point right before starvation and three refed time points after starvation (1, 2 and 3 hr of refeeding). This will allow us to compare the expression of the gene before starvation, during starvation and after starvation. We reasoned that these five time points would produce optimal differences in genes whose expression is dependent on metabolic state.

We found 708 genes that change their expression in these 5 time points (Supplemental Table S2). Because our sampling time points represent differences in metabolism as well as in time, we expect that some genes changed not because of the metabolic state, but because of time (e.g. developmental genes). There are 300 genes total that showed steady decrease (274) or increase (26) throughout the time course, independent of the metabolic state (Supplemental Tables S3 and S4 respectively). Most of the decreased genes are collagens or major sperm proteins, which are development stage specific. The expression of these genes was reduced over time likely because these genes are only necessary in the young adult stage. The 26 up-regulated genes were without known functions or known homologs, or we failed to find specific characters in common.

We further divided the remaining 408 genes into two groups: one is a group of genes upregulated by starvation then downregulated by refeeding (284 genes, Supplemental Table S5). The other group is genes downregulated by starvation then upregulated by refeeding (124 genes, Supplemental Table S6). Many of the genes that are upregulated during starvation and downregulated after refeeding are known to be involved in stress responses. Because the expression of these genes is tied to metabolic changes, both groups of genes could regulate feeding behavior.

We noticed that among 284 genes that are upregulated by starvation and downregulated by refeeding, 28 of them are members of the nuclear hormone receptor (NHR) family (Table 1). The microarray results were validated by qRT-PCR (Table 1). The microarray results regarding the 28 NHRs is interesting because of three reasons: First, even considering the large number of NHR genes in worms (293), 9.6% is highly over-represented compared to other groups of genes with large numbers such as GPCR. Among more than 1000 GPCR genes, there are only 10 GPCRs (1%) whose expression is changed. Second, all 28 NHRs change their expression in the same way: they are upregulated during starvation and downregulated during refeeding, suggesting a correlation between expression of these NHRs and metabolic status. Third, and most importantly to us, although NRs play critical roles in metabolism in mammals, their roles in feeding behavior are not clear. This is also true in *C. elegans*; many NHRs play important roles in developmental decisions, fat metabolism and longevity, but none have been shown to influence feeding behavior.

We used a TGFβ mutant to examine whether the expression of the 28 NHRs would change in mutants defective in satiety behavior. Previously, we showed that a neuronal TGFβ pathway regulates satiety quiescence, potentially sensing the animal’s nutritional status^9^,^27^. The mutants with reduced TGFβ signaling have reduced satiety quiescence, increase feeding and store more fat. We reasoned that since starvation increases the expression of the 28 NHRs, then their expression would also be increased in TGFβ mutants that cannot sense its well-fed status, and thus show starvation-like responses. The expression of the 28 NHRs is upregulated in a TGFβ ligand mutant (*daf-7*) (Table 2). This supports the idea that the expression of the 28 NHRs could be linked to metabolic status; both in the starved wild-type animals and in the mutants that behave as if starved, the 28 NHRs’ expression was increased.

The observation that the expressions of the 28 NHRs are upregulated together during starvation suggests possible common transcription factor(s) that control these NHRs. An analysis of the modENCODE database of *C. elegans* revealed 7 transcription factors which have binding sites in 19 of the 28 NHRs: *pha-4, daf-16, skn-1, blmp-1, elt-3, mdl-1* and *pqm-1* (Fig. 1G). There were insufficient data for the remaining 9 NHRs. PHA-4 and DAF-16 are very well known to regulate critical development processes; their human orthologs are FOXA and FOXO respectively. DAF-16, in particular, is downstream of insulin signaling and mediates various starvation responses including dauer decision^28^. SKN-1 is important for diet-restricted longevity^29^. The fact that the 28 NHRs have single or multiple binding sites for these transcription factors and are thus potentially regulated by these factors again support the hypothesis that these 28 NHRs play a role in the starvation response.

### 11 of the 28 NHRs regulate fat storage and satiety quiescence

Next, we further examined whether the 28 NHRs regulate satiety quiescence by altering fat storage, because we showed that fat metabolism influenced satiety quiescence and because NRs play roles in fat metabolism. We have developed an automated method to measure an animal’s individual locomotive activity and analyze its behavioral status^30^. We performed RNAi to individually knock down each of the 28 NHRs and measured the satiety quiescence and fat storage in these animals (Table 3, see Materials and Methods).

We have two methods to measure satiety quiescence: One is refeeding after starvation and the other is to have the animal feed ad-lib. The wild type and mutants we have tested show consistent trends in both methods; animals show satiety in the ‘refeeding after starvation’ method also show satiety in the ‘ad-lib’ method and visa versa. We decided to use the ad-lib method to test the NHRs because it allowed us to test many animals in a reasonable period of time. In addition, the ad-lib method is more useful in cases when the starvation and refeeding method occludes any difference due to a ceiling effect on satiety behavior. The automated ad-lib method was performed as previously described^30^. Briefly, worms were isolated from L4 for 24 hours to synchronize their ages, and then placed on a new bacterial lawn to be tested under video cameras. 11 NHRs of the 28 NHRs showed changes in both satiety and fat storage. We categorized the 11 NHRs in 4 different groups depending on the correlations between two phenotypes. Group 1 showed both satiety quiescence and fat storage decrease; *nhr-170* and *nhr-206* belong to this group. It is possible that in the absence of these NHRs, *C. elegans* stored less fat due to a defect in fat synthesis or storage, which in turn would reduce satiety quiescence. Group 2 showed both satiety quiescence and fat storage increase; *nhr-21* and *nhr-64* belong to this group. A simple explanation of this phenotype is that this is the opposite case of the Group 1, where in the absence of these genes *C. elegans* fat synthesis and storage increase, which in turn increases satiety quiescence. We are particularly encouraged from identification of NHR-64 as such a regulator, because it has been shown that NHR-64 antagonizes the SREBP pathway through a genetic interaction with POD-2^31^. Therefore, lack of *nhr-64* should show opposite phenotypes to those of SREBP mutants: increase in fat storage and satiety. It is possible that in the absence of NHR-64, the SREBP signal is enhanced and the animal stores more fat and enhances satiety quiescence.

The results of Group 1 and 2 suggest that altering fat storage could alter satiety quiescence. However, fat storage can also be a result of misregulation of feeding behavior; we have shown that certain mutants defective in sensing nutritional status misregulate satiety behavior and end up storing either more or less fat^9^. Group 3 and 4 could be those cases. Group 3 showed increased satiety quiescence but decreased fat storage. Five NHRs belong to this group (*nhr-8*, *nhr-50*, *nhr-99*, *nhr-120, nhr-144*). Group 4 showed decreased satiety quiescence but increased fat storage; *nhr-162* and *nhr-212* belong to this group. Our previous study showed that mutants that cannot sense their metabolic status showed such an inverse relationship between feeding and fat storage. For instance, TGFβ mutants reduce satiety quiescence and increase fat storage because the mutant constantly eats^27^. On the contrary, a gain of function mutant of a cGMP-activated-kinase (EGL-4) showed excessive satiety quiescence but stored less fat^9^,^32^. Therefore, the 7 NHRs belonging to group 3 and 4 could link fat storage to feeding behavior and potentially work as a sensor; in the absence of these genes the animals could not sense their metabolic status and therefore do not show proper feeding behavior. Almost nothing is known about the function or tissue-expression patterns of these 7 NHRs except NHR-8. NHR-8 plays critical roles in xenobiotic detoxification, fat metabolism and cholesterol metabolism^33^,^34^,^35^, all of which could be involved in regulation of feeding behavior by modifying the sensation of the metabolic status of the animals.

Seven NHRs among the remaining 17 NHRs did not change feeding behavior. We consider them as negative controls; their expression is affected by metabolic changes but they do not alter the two phenotypes we examined. It is possible that those NHRs regulate other aspects of starvation responses.

## Discussion

In this study we show that the conserved fatty acid synthesis pathway regulates satiety quiescence in *C. elegans*. An RNAi screen identified an ACC as a potential mediator between fat metabolism and feeding behavior. It has been shown that one of the ACCs in mammals is downstream of the SREBP pathway^19^. Our data support that this pathway could also exist in *C. elegans*. Profiling of starved and refed animals identified 28 NHRs whose expression significantly changes by starvation and refeeding. Eleven of these NHRs also alter fat storage and satiety quiescence. Interestingly, one of the NHRs we found from RNAi was NHR-64, which is known to antagonize the SREBP pathway in *C. elegans* via a genetic interaction with POD-2, a *C. elegans* ACC.

Depleting energy storage promotes feeding; in mammals, leptin, a peptide hormone, is released from adipose tissue to regulate feeding so that an animal can gauge how much fat storage it has. Lack of this signaling system causes morbid obesity, showing the importance of the balance between fat storage and proper feeding to maintain health. Leptin only exists in vertebrates, however, raising a question of how the brain senses fat storage to control feeding in invertebrate animals. Unlike leptin, NHRs are conserved in both vertebrates and invertebrates and play critical roles in metabolism, often acting as receptors for steroid or lipid hormones (e.g. estrogen or ecdysone). Yet, it is unknown whether any NHRs regulate feeding behavior. Our finding that certain NHRs regulate feeding and fat storage suggests that NHRs might mediate leptin-like responses in other invertebrate animals. Therefore our study provides useful insights for a potential role of NHRs in relaying metabolic status to feeding behavior.

## Methods

### Strains

Worms were cultured and handled as described previously^36^ with the following modifications: worms were routinely grown on NGMSR plates^37^. All worms were maintained at 20 °C on *E. coli* strain HB101 unless indicated otherwise. The wild-type strain was *C. elegans* variant Bristol, strain N2. Mutant strains used were CB1372 *daf-7*(*e1372ts*) *III*, CB1376 *daf-3*(*e1376*) *X*, HY520 *pod-2*(*ye60*) II, BX156 *fat-6* IV (*tm331*); *fat-7* V (*wa36*), CE541 *sbp-1*(*ep79*) III.

### Satiety quiescence assay

Satiety quiescence was measured as previously described^9^. Briefly, worms were fasted for 12 hrs and refed for 3 or 6 hrs to examine satiety quiescence. Once worms were found to be quiescent, the duration was measured for 10 worms then averaged. Satiety quiescence of RNAi of the 28 NHRs was measured with an automated method^27^. In short, the automated method involves recording individual worm’s behavior for 30 minutes, then using a custom written tracker in MATLAB to track their movement. The output for this is coordinate data, which is further analyzed using a custom written Mathematica notebook to break their behavior down into categories based on locomotive speed using a Hidden Markov Model^30^. For the non-starved assay on DA837 (in Fig. 1D), the worms were picked at L4s to synchronize the age and then singly tested for satiety quiescence 12 hrs from L4s^9^.

### RNAi screen for deficiency in satiety quiescence

Among 183 major metabolic genes selected and tested by Wang *et al.*^24^, 143 available clones from Ahringer feeding library were tested by bacteria-mediated feeding RNAi (See Supplementary Table S1)^38^,^39^. The plates containing NGM agar with 1 mM IPTG and 50 mg/ml carbenicillin were inoculated with bacterial cultures grown 16–18 hrs for each targeted gene. Three L4 stage worms were transferred in the plates for each gene and left at 20 °C. 36 hrs later adults were removed. Another 36 hrs later, 20 L4 worms were picked to perform the satiety quiescence assay^9^. For each test, 7 concurrent control RNAi treated worms and 7 each RNAi treated worms were used. Once an RNAi changed satiety quiescence, RNAi of the gene was repeated three times.

### Fat storage measurement

Fat storage of 1 day old adults (24 hrs from L4s) was quantified as described using oil red O^40^. The same stage worms were used to measure their satiety quiescence. L4 worms were transferred to the RNAi plates. After they became adults and laid eggs, adult worms were removed. After 24 hrs from the L4s of the second generation, the adults were tested for fat storage by oil red O staining as described^8^. For oil red O staining, 100–200 of 24 hr old adult from L4 were harvested and washed by 1× PBS, pH7.4. The worms were permeabilized and fixed after being resuspended in 400 μl of PBS to which an equal volume of 2× MRWB (160 mM KCl, 40 mM NaCl, 14 mM Na_2_EGTA, 1 mM spermidine-HCl, 0.4 mM spermine, 30 mM Na-PIPES pH 7.4, 0.2% β-mercaptoethanol) buffer containing 4% paraformaldehyde (PFA) was added. Samples were gently rocked for 2 hrs at room temperature. The worms were allowed to settle by gravity, buffer was aspirated, and the worms were washed with 1× PBS containing glycine to remove PFA and then washed with 1× PBS (pH7.4). The worms were resuspended in 60% isopropanol and incubated for 15 minutes at room temperature to dehydrate. After allowing worms to settle, isopropanol was removed, 1 mL of 60% oil red dye was added, and animals were incubated overnight with rocking. The dye was removed after allowing the worms to settle, and 200 μL of 1× PBS 0.01% Triton X-100 was added. The worms were observed under differential interference contrast (DIC) or a GFP filter using a Zeiss Axio Imager 2 at 10× magnification. Images were acquired using Zeiss Axiovision software.

### Microarray

#### RNA extraction

Total RNA was extracted and the quality evaluated using a sample processing method previously established in our laboratory^41^. Total RNA was extracted from 400 *C. elegans* worms using the MagMAX™-96 for Microarrays Total RNA Isolation Kit (InvitrogenTM Life Technologies, Carlsbad, CA), in an automated fashion using the magnetic particle processors MagMAXTM Express. RNA purity was judged by spectrophotometry at 260, 270, and 280 nm. RNA integrity as well as cDNA and cRNA synthesis products were assessed by running 1 μL of every sample in RNA 6000 Nano LabChips® on the 2100 Bioanalyzer (Agilent Technologies, Foster City, CA).

#### Gene expression microarray analyses

The Affymetrix® protocol utilized for our microarray analyses has been previously described^41^ and was used with the following modifications. Starting from 500 ng of total RNA, we performed a single-strand cDNA synthesis primed with a T7- (dT24) oligonucleotide. Second strand cDNA synthesis was performed with the *E. coli* DNA Polymerase I, and biotinylation of the cRNA was achieved by *in vitro* transcription (IVT) reaction using the using the GeneChip® 3′ IVT Express Kit (Affymetrix, Santa Clara, CA). After a 37 °C-incubation for 16 hrs, the labeled cRNA was purified using the cRNA cleanup reagents from the GeneChip® Sample Cleanup Module. As per the Affymetrix® protocol, 10 μg of fragmented cRNA were hybridized on the GeneChip® *C. elegans* Genome array (Affymetrix Inc., Santa Clara, CA) for 16 hours at 60 rpm in a 45 °C hybridization oven. The GeneChip® *C. elegans* Genome array provides a comprehensive coverage of the transcribed *C. elegans* genome by analyzing the expression level of over 22,500 well-characterized transcripts. The arrays were washed and stained with streptavidin phycoerythrin (SAPE; Molecular Probes, Eugene, OR) in the Affymetrix® fluidics workstation. Every chip was scanned at a high resolution, on the Affymetrix® GeneChip Scanner 3000 7G according to the GeneChip® Expression Analysis Technical Manual procedures (Affymetrix). After scanning, the raw intensities for every probe were stored in electronic files (in .DAT and .CEL formats) by the GeneChip® Operating Software v1.4 (GCOS) (Affymetrix). Overall quality of each array was assessed by monitoring the 3′/5′ ratios for the housekeeping gene, glyceraldehyde 3-phosphate dehydrogenase (Gapdh), and the percentage of “Present” genes (%P). Arrays exhibiting Gapdh 3′/5′ < 3.0 and %P > 40% were considered good quality arrays.

#### Statistical analysis

For the microarray data analysis, background correction, normalization, and estimation of probe set expression summaries was performed using the log-scale robust multi-array analysis (RMA) method^42^. Hierarchical cluster analyses were performed with the BRB-ArrayTools v3.1.0 (Biometric Research Branch, National Cancer Institute), an Excel add-in that collates microarray data with sample annotations. In order to identify differentially expressed genes between the different classes, we performed t-tests for each probe set from biological replicates in each class. Statistical significance for multivariate analysis to assess probe set specific false discovery rates (FDR) was performed by estimating the q-values, using the Bioconductor q-value package^43^.

### Quantitative RT-PCR

#### Total RNA preparation

*C. elegans* (from mixed and individual stages) were grown on NGM plates at 20 °C or 25 °C, washed with M9 buffer and re-suspended in Trizol (Invitrogen). After vortexing for 60 s, the mixture was frozen in liquid nitrogen and thawed at room temperature. After chloroform extraction, DNA was removed using DNase I. After ethanol precipitation, the air-dried pellet was dissolved in DEPC water.

#### cDNA preparation and qPCR

1~2 μg of total RNA in a 20 μl reaction was used to synthesize the cDNA (synthesis kit, Biovision, Bio65043). Quantitative RT-PCR was carried out in a C-1000 thermal cycler Real-Time PCR system (Biorad, CFX96 optics module) and analyzed using the Ct method^44^. mRNA levels of *ama-1* (RNA polymerase II) was used for normalization as previously described^45^. The average of at least three repeats was used for each data point. Oligonucleotides used for qPCR and their sequences are shown in Supplementary Table S7.

## Additional Information

**How to cite this article**: Hyun, M. *et al.* Fat Metabolism Regulates Satiety Behavior in *C. elegans.* *Sci. Rep.* **6**, 24841; doi: 10.1038/srep24841 (2016).

## Supplementary Material

Supplementary Information

## Figures and Tables

**Table 1:** Nuclear hormone receptor genes whose expression changes during starvation and refeeding.

Genes	Fold Changes (Microarray)		Fold Changes (qRT-PCR)		Putative Homologs
	12S	12S 1RF	12S	12S 1RF	
*nhr-8*	3.91	2.70	6.07	2.68	VDR
*nhr-18*	5.50	1.86	2.47	1.33	CYP21
*nhr-21*	3.11	0.87	2.61	1.10	HNF4-alpha
*nhr-32*	3.14	0.79	2.86	1.09	HNF4-alpha
*nhr-36*	2.66	0.94	4.28	1.05	HNF4-alpha
*nhr-40*	1.32	1.28	1.98	1.53	HNF4-alpha
*nhr-50*	2.28	1.68	2.74	1.50	HNF4-alpha
*nhr-59*	2.30	1.16	6.30	2.71	CAR
*nhr-64*	3.03	1.11	8.23	2.53	HNF4-alpha
*nhr-79*	2.00	0.90	3.52	1.22	NR1H3
*nhr-90*	4.81	1.68	5.13	1.16	HNF4-alpha
*nhr-99*	2.72	0.96	16.51	0.75	RAR-beta
*nhr-101*	5.12	1.21	4.61	0.89	HNF4-gamma
*nhr-109*	2.61	1.28	2.01	0.97	RAR-alpha
*nhr-110*	3.46	1.04	2.09	1.02	VDR
*nhr-117*	2.62	0.98	19.85	0.86	PPAR-alpha
*nhr-120*	2.84	0.92	5.41	1.18	RXR-alpha
*nhr-122*	4.20	1.07	3.46	1.37	HNF4-alpha
*nhr-133*	3.29	0.90	5.17	1.29	NR2E1
*nhr-143*	3.22	1.03	4.91	1.93	HNF4-gamma
*nhr-144*	2.22	1.02	5.54	1.37	no homolog
*nhr-162*	2.79	1.11	1.69	0.75	HNF4-gamma
*nhr-163*	2.39	0.89	4.69	1.70	HNF4-gamma
*nhr-170*	3.60	1.55	15.07	4.72	RAR-alpha
*nhr-206*	3.90	1.13	2.47	1.11	Mineralocorticoid receptor
*nhr-211*	1.99	0.99	2.33	1.49	HNF4-gamma
*nhr-212*	2.35	1.39	2.35	1.69	LXR-beta
*nhr-232*	3.89	0.90	1.88	1.60	RXR-beta

The fold changes were normalized by the level of mRNA in well-fed. 12S: 12 hrs of starvation, 12S 1RF: 12 hrs of starvation and 1 hr of refeeding.

**Table 2:** The 28 NHRs’ expression is upregulated in a *daf-7* mutant.

NHRs	*daf-7*(normalize to N2)
*nhr-8*	2.685
*nhr-18*	7.161
*nhr-21*	5.819
*nhr-32*	4.229
*nhr-36*	2.382
*nhr-40*	3.577
*nhr-50*	7.814
*nhr-59*	6.805
*nhr-64*	3.758
*nhr-79*	5.492
*nhr-90*	9.011
*nhr-99*	18.957
*nhr-101*	9.307
*nhr-109*	15.735
*nhr-110*	9.607
*nhr-117*	28.203
*nhr-120*	3.543
*nhr-122*	5.626
*nhr-133*	4.131
*nhr-143*	6.625
*nhr-144*	6.630
*nhr-162*	3.599
*nhr-163*	4.905
*nhr-170*	7.867
*nhr-206*	8.001
*nhr-211*	5.540
*nhr-212*	5.836
*nhr-232*	3.086
daf-12*	1.294
daf-16*	0.869

^*^The levels of *daf-12* and *daf-16* were tested as controls to show that the increased expression of the 28 is not because of general increase in transcription in *daf-7* mutants.

**Table 3:** Eleven NHRs that change satiety quiescence and fat storage when knocked down.

RNAi	Quiescence ratio to control (%)	ORO	pORO	pSQ
Group 1: Both decreased				
*nhr-170*	0.62	less fat	***	***
*nhr-206*	0.45	less fat	**	***
Group 2: Both increased				
*nhr-21*	1.62	more fat	***	***
*nhr-64*	1.74	more fat^#^		***
Group 3: SQ increased, Fat decreased				
*nhr-8*	1.26	less fat	*	***
*nhr-50*	3.53	less fat	***	***
*nhr-99*	1.69	less fat	***	**
*nhr-120*	2.04	less fat	**	***
*nhr-144*	2.26	less fat	***	***
Group 4: SQ decreased, Fat increased				
*nhr-162*	0.64	more fat	**	***
*nhr-212*	0.44	more fat	***	***
No or weak correlation				
*nhr-18*	1.00	less fat	***	N.S
*nhr-36*	1.12	less fat	***	N.S
*nhr-117*	0.90	less fat	***	N.S
*nhr-143*	0.85	more fat	***	N.S
*nhr-163*	0.84	more fat	***	N.S
*nhr-232*	1.03	more fat	***	N.S
*nhr-79*	2.09	no change	N.S	***
*nhr-90*	1.73	no change	N.S	***
*nhr-110*	1.76	no change	N.S	***
*nhr-211*	0.60	no change	N.S	***
*nhr-32*	1.30	less fat	**	N.S
*nhr-40*	1.18	no change	N.S	N.S
*nhr-59*	0.98	more fat	*	N.S
*nhr-101*	1.54	less fat	*	*
*nhr-109*	1.17	no change	N.S	*
*nhr-122*	1.27	no change	N.S	N.S
*nhr-133*	1.15	no change	N.S	N.S

^#^Result based on the report by Liang *et al.*^31^.

Quiescence ratio is normalized to the concurrent control (see Materials and Methods). 12–25 samples were tested for each RNAi and 7–10 samples for the control. ORO is oil red O staining, pORO is statistical significance for oil red O staining. pSQ is statistical significance for satiety quiescence. *p < *0.05*, **p < *0.01*, ***p < *0.001* by Student’s *t* test.
