# Supplementary material for: Fat Metabolism Regulates Satiety Behavior in C. elegans
Source: Sci Rep. 2016 Apr 21;6:24841. doi: 10.1038/srep24841 (PMC4838937; doi:10.1038/srep24841)
Supplement: Supplementary Information [file srep24841-s1.pdf]

Fat Metabolism Regulates Satiety behavior in *C. elegans*

Moonjung Hyun[1], Kristen Davis[1], Inhwan Lee[1], Jeongho Kim[2], Catherine Dumur[3], Young-Jai You[1*]

1. Department of Biochemistry and Molecular Biology, Virginia Commonwealth University, Richmond, Virginia
2. Department of Biological Sciences, Inha University, Incheon, 402-751, South Korea
3. Department of Pathology, Virginia Commonwealth University, Richmond, VA 23298

* Author who corresponds.

Supplemental Materials

Supplemental Table S1: RNAi clones tested for satiety quiescence

chromosome I	genes	descriptions
C30F12.7		Isocitrate dehydrogenase
C34B2.7	*sdha-2*	Succinate dehydrogenase
C55B7.4	*acdh-1*	acyl-CoA dehydrogenase
F08A8.1		Acyl-CoA oxidase
F08A8.2		Acyl-CoA oxidase
F08A8.3		Acyl-CoA oxidase
F08A8.4		Acyl-CoA oxidase
F14B4.2		Hexokinase
F25H5.3		Pyruvate Kinase
F32H2.5	*fasn-1*	fatty acid synthase
F32H2.6	*phi-46*	fatty acid synthase
F36A2.3		malate dehydrogenase
F43G9.1		Isocitrate dehydrogenase
F47G4.3	*gpdh-1*	Glycerol-3-phosphate dehydrogenase
F47G6.2		Acyl-CoA synthetase
F57B10.3		Phosphoglycerate mutase
F57B10.7	*tre-1*	Neutral trehalase
H25P06.1		Hexokinase
K06A5.6	*acdh-3*	acyl-CoA dehydrogenase
K07A3.1	*fbp-1*	Fructose-1,6-bisphosphatase
R11A5.4		Phosphoenolpyruvate carboxykinase
T03F1.3	*pgk-1*	3-phosphoglycerate kinase
T10E9.9	*acdh-4*	Short-chain acyl-CoA dehydrogenase
T25G3.4		Glycerol-3-phosphate dehydrogenase
W02D3.5	*lbp-6*	Fatty acid-binding protein FABP
W02D3.7	*lbp-5*	Fatty acid-binding protein FABP
Y110A7A.6		fructose-6-phospate 2-kinase/ fructose-2,6-biphosphate
Y54E5A.1		Fatty acid desaturase
Y65B4BL.5	*acs-13*	Acyl-CoA synthetase
Y87G2A.8	*gpi-1*	Glucose-6-phosphate isomerase
chromosome II		
C03H5.4		phospholipase
C07E3.9		phospholipase
C23H3.7	*tre-5*	Neutral trehalase
C44B7.9	*pmp-2*	Peroxisomal long-chain acyl-CoA transporter
E04F6.5	*acdh-12*	acyl-CoA dehydrogenase
F19H8.1	*tps-2*	Trehalose-6-phosphate synthase
F28A10.6	*acdh-9*	acyl-CoA dehydrogenase
F33H1.2	*gpd-4*	Glyceraldehyde 3-phosphate dehydrogenase
F41C3.3	*acs-11*	Acyl-CoA synthetase
F42A8.2	*sdhb-1*	Succinate dehydrogenase
K05F1.3	*acdh-8*	acyl-CoA dehydrogenase
R06F6.9	*ech-4*	Enoyl-CoA hydratase/isomerase
R07C3.4		Acyl-CoA synthetase
R11F4.1		Glycerol Kinase
T02G5.4		Acetyl-CoA acetyltransferase
T02G5.7		Acetyl-CoA acetyltransferase
T02G5.8	*kat-1*	Acetyl-CoA acetyltransferase
T05C12.3		Enoyl-ACP reductase

T05H10.6		Pyruvate dehydrogenase
T21B10.2	*enol-1*	Enolase
W01C9.4		Enoyl-ACP reductase
W09B6.1	*pod-2*	fatty acid synthase
Y17G7B.7	*tpi-1*	Triosephosphate isomerase
Y46G5A.31	*gsy-1*	Glycogen synthase
Y48B6A.12		NADP+-dependent malic enzyme
Y57A10C.6		Peroxisomal 3-ketoacyl-CoA-thiolase
chromosome III		
C40H1.2		Predicted lipase
C48B4.1		Acyl-CoA oxidase
F01F1.12		Fructose-biphosphate aldolase
F20H11.3	*mdh-1*	Malate dehydrogenase
F23H11.3		Succinyl-CoA synthetase
F37C12.7	*acs-16*	Acyl-CoA synthetase
F48E8.3		Succinate dehydrogenase
F54C8.1		3-hydroxyacyl-CoA dehydrogenase
F54H12.1	*aco-2*	Aconitase/homoaconitase
K08E3.5		UDP-glucose pyrophosphorylase
K11H3.1	*gpdh-2*	Glycerol-3-phosphate dehydrogenase
T05D4.1		Fructose-biphosphate aldolase
T05G5.6	*ech-6*	Enoyl-CoA hydratase
T07C4.7	*mev-1*	Succinate dehydrogenase
T20G5.2	*cts-1*	Citrate synthase
W05G11.6		Phosphoenolpyruvate carboxykinase
Y48G9A.10	*cpt-3*	Carnitine O-acyltransferase CPT
Y76A2B.3	*acs-14*	Acyl-CoA synthetase
chromosome IV		
C02B10.1	*ivd-1*	Isovaleryl-CoA dehydrogenase
C04C3.3		Pyruvate dehydrogenase
C28C12.9	*acdh-13*	Very-long-chain acyl-CoA dehydrogenase
C30H6.7		Dihydrolipoamide acetyltransferase
C50F7.4		Succinyl-CoA synthetase
F01G10.2	*ech-8*	Hydroxyacyl-CoA dehydrogenase/enoyl-CoA hydratase
F01G10.3	*ech-9*	Hydroxyacyl-CoA dehydrogenase/enoyl-CoA hydratase
F01G4.2	*ard-1*	3-hydroxyacyl-CoA dehydrogenase
F28D1.9		Long-chain fatty acid transport protein
F33D4.4		Fatty acid desaturase
F38H4.8	*ech-2*	Enoyl-CoA isomerase
H04M03.1		Phosphoenolpyruvate carboxykinase
K02B2.1		phospho-fructo-kinase
LLC1.3		Dihydrolipoamide dehydrogenase
R07H5.2	*cpt-2*	Carnitine O-acyltransferase CPT
R09E10.3		Long-chain acyl-CoA synthetases
T02D1.5	*pmp-4*	Peroxisomal long-chain acyl-CoA transporter
T05A12.2	*tre-2*	Neutral trehalase
T13F2.1	*fat-4*	Delta 6-fatty acid desaturase/delta-8 sphingolipid desaturase
T22B11.5		2-oxoglutarate dehydrogenase
VZK822L.1	*fat-6*	Fatty acid desaturase
W02A2.1	*fat-2*	Oleate desaturase/linoleate desaturase
W08D2.4	*fat-3*	Delta 6-fatty acid desaturase/delta-8 sphingolipid desaturase
Y67H2A.8	*fat-1*	Oleate desaturase/linoleate desaturase
Y77E11A.1		Hexokinase
ZK593.1		Pyruvate Kinase
chromosome V		

C05E4.9	*gei-7*	Malate Synthase
C29F3.1	*ech-1*	Hydroxyacyl-CoA dehydrogenase/enoyl-CoA hydratase
C50F4.2		phospho-fructo-kinase
D2023.2	*pyc-1*	Pyruvate carboxylase
F09F3.9	*cpt-5*	Carnitine O-acyltransferase CPT
F10D2.9	*fat-7*	Fatty acid desaturase
F23B12.5		Dihydrolipoamide acetyltransferase
F25C8.1		Acyl-CoA oxidase
F28F8.2	*acs-2*	Long chain fatty acid acyl-CoA ligase
F43H9.1	*ech-3*	Enoyl-CoA hydratase
F46E10.1	*acs-1*	Acyl-CoA synthetase
F46E10.10		Malate dehydrogenase
F47B8.10		Glucose-6-phosphate transporter
F53C11.3		Enoyl-ACP reductase
K04A8.5		Triglyceride lipase
T22F3.3		Glycogen phosphorylase
T22G5.2	*lbp-7*	Fatty acid-binding protein FABP
T22G5.6	*lbp-8*	Fatty acid-binding protein FABP
W01A11.5	*cpt-6*	Carnitine O-acyltransferase CPT
W02F12.5		2-oxoglutarate dehydrogenase
W06D12.3	*fat-5*	Fatty acid desaturase
Y40B10A.1	*lbp-9*	Fatty acid-binding protein FABP
Y50E8A.6		Glycerol-3-phosphate dehydrogenase
ZK742.5	*lbp-4*	Fatty acid-binding protein FABP
chromosome X		
B0272.3		3-hydroxyacyl-CoA dehydrogenase
C05G5.4		Succinyl-CoA synthetase
C34F6.8		NADP-dependent isocitrate dehydrogenase
C37E2.1		Isocitrate dehydrogenase
C46C11.1		hormone sensitive lipase
C46F4.2	*acs-17*	Acyl-CoA synthetase
D1005.2		UDP-glucose pyrophosphorylase
F09E10.3	*dhs-25*	Mitochondrial beta-ketoacyl-ACP reductase
F40F4.2	*lbp-2*	Fatty acid-binding protein FABP
F40F4.3	*lbp-1*	Fatty acid-binding protein FABP
F40F4.4	*lbp-3*	Fatty acid-binding protein FABP
F47B10.1		Succinyl-CoA synthetase
F59F4.1		Acyl-CoA oxidase
K10B3.7	*gpd-3*	Glyceraldehyde 3-phosphate dehydrogenase
K10B3.8	*gpd-2*	Glyceraldehyde 3-phosphate dehydrogenase
T08G2.3	*acdh-10*	Medium-chain acyl-CoA dehydrogenase
T22B7.7		Acyl-CoA thioesterase
Y71H10A.1		phospho-fructo-kinase
ZK54.2	*tps-1*	Trehalose-6-phosphate synthase

Supplemental Table S2: The list of the genes whose expression changed significantly by starvation and refeeding (Total 707 genes)

Gene symbols	Description	Well fed (Mean)	12 hrs starved (Mean)	1 hr refed (Mean)	2 hr refed (Mean)	3 hr refed (Mean)	p-value
aakb-1	aakb-1 encodes one of two C. elegans AMP kinase (AMPK) beta regulatory subunits.	134.17	249.04	147.30	134.92	137.36	9.74E-05
aakg-4	aakg-4 encodes one of five C. elegans AMP kinase (AMPK) gamma regulatory subunits.	21.64	64.86	25.90	20.13	21.29	1.70E-05
aat-4	aat-4 encodes a predicted amino acid transporter catalytic subunit; unlike catalytic subunits in other organisms, however, AAT-4 does not contain the highly conserved cysteine residue known to facilitate covalent interaction with a glycoprotein subunit, suggesting that AAT-4 does not require this residue for heterodimer formation or, alternatively, does not require the glycoprotein subunit for function.	61.04	19.69	87.70	89.27	89.59	1.48E-05
acbp-3	acbp-3 encodes a highly conserved protein containing a functional acyl-CoA-binding binding domain required for protein stability and ligand binding; mutant contains approximately 30% fewer lipid droplets and 20% less triglycerides; loss of ACBP-3 results in 35-40% reduction in lipid chain unsaturation; ACBP-3::GFP is predominantly expressed in hypodermis, body wall muscles and pharynx.	140.44	170.32	225.31	311.04	304.94	1.82E-04
acdh-5	acdh-5 is an ortholog of human GCDH (glutaryl-CoA dehydrogenase), ACAD10 (acyl-CoA dehydrogenase 10), ACAD9 (acyl-CoA dehydrogenase 9), ACOX3 (acyl-CoA oxidase 3, pristanoyl) and ACOXL (acyl-CoA oxidase-like); acdh-5 is predicted to have acyl-CoA dehydrogenase activity and flavin adenine dinucleotide binding activity, based on protein domain information.	23.58	13.37	14.51	12.10	11.39	1.53E-04
acdh-8	acdh-8 is an ortholog of human ACADM (acyl-CoA dehydrogenase, C-4 to C-12 straight chain); acdh-8 is predicted to have acyl-CoA dehydrogenase activity and flavin adenine dinucleotide binding activity, based on protein domain information.	397.48	164.72	116.97	65.57	59.94	1.61E-04
acl-1	acl-1 encodes a glycerol-3-phosphate acyltransferase; ACL-1 is predicted to be a membrane protein that plays a role in phospholipid biosynthesis; acl-1 expression decreases gradually throughout aging.	415.57	255.80	706.81	1098.93	1179.56	2.06E-04
acl-4	acl-4	177.33	153.64	176.44	295.51	292.91	7.46E-05
acly-1	acly-1 is an ortholog of human ACLY (ATP citrate lyase); acly-1 is predicted to have ATP citrate synthase activity, ATP binding activity, and cofactor binding activity, based on protein domain information.	451.39	122.18	355.61	746.30	899.49	7.49E-05
acox-2	acox-2 is an ortholog of human ACOX2 (acyl-CoA oxidase 2, branched chain) and ACOX1 (acyl-CoA oxidase 1, palmitoyl); acox-2 is predicted to have acyl-CoA dehydrogenase activity, acyl-CoA oxidase activity, and flavin adenine dinucleotide binding activity, based on protein domain information.	69.67	20.48	21.98	20.99	23.12	1.69E-05
acs-1	acs-1 is an ortholog of human ACSF2 (acyl-CoA synthetase family member 2); acs-1 is predicted to have catalytic activity, based on protein domain information.	975.85	498.01	1547.99	2794.60	2330.30	1.32E-04
acs-18	acs-18 is an ortholog of human SLC27A3 (solute carrier family 27 (fatty acid transporter), member 3), SLC27A6 (solute carrier family 27 (fatty acid transporter), member 6), SLC27A2 (solute carrier family 27 (fatty acid transporter), member 2), SLC27A4 (solute carrier family 27 (fatty acid transporter), member 4) and SLC27A1 (solute carrier family 27 (fatty acid transporter), member 1); acs-18 is predicted to have catalytic activity, based on protein domain information.	42.11	25.08	20.45	16.24	15.54	1.05E-04
acs-6	fatty acid coA synthetase family; acs-6 is predicted to have catalytic activity, based on protein domain information.	81.70	41.26	32.44	26.05	27.05	1.14E-04
aex-6	aex-6 encodes a Rab small GTPase; aex-6 was identified in screens for defecation defective mutants and high-temperature-induced dauer formation mutants; aex-6 appears to act in the insulin branch of the dauer pathway; aex-6 is genetically required for two different steps of normal defecation in the hermaphrodite digestive tract; since its mutant phenotype resembles that induced by ablation of the interneuron AVL and the motor neuron DVB, aex-6 is likely to be required for the function or activation of these neurons rather than for any of their specific neurotransmitter systems; aex-6::gfp reporter fusions are expressed in neurons and in the intestine; in the nervous system, AEX-6	61.23	109.04	103.76	95.51	84.47	6.93E-05

	localizes to synapse-rich regions and partially colocalizes with synaptic vesicle-associated RAB-3.						
amt-4	amt-4 encodes a member of the ammonium transporter protein family.	136.24	85.65	321.99	395.34	276.74	2.33E-05
ant-1.4	ant-1.4 encodes an ortholog of the human adenine nucleotide translocase ANT genes, the other ANT genes in C. elegans include ant-1.1, ant-1.2 and ant-1.3; phylogenetic analysis indicates that ant-1.4 is specific to C. elegans, resulting from a recent duplication following the speciation of the common ancestor of C. elegans, C. briggsae, and C. remanei; by homology, ANT-1.4 is predicted to mediate the exchange of ATP generated in the mitochondria for cytosolic ADP; ANT-1.4 GFP fusion protein is expressed in a pair of head neurons, amphid socket and sheath cells and in a few body-wall muscle and vulval muscle cells.	56.08	26.79	20.38	19.12	19.70	5.13E-05
apm-1	The apm-1 gene encodes an adaptin: specifically, it encodes an ortholog of the mu1-II subunit of adaptor protein complex 1 (AP-1).	119.60	293.87	161.92	184.93	189.37	2.76E-05
asp-8	asp-8 is an ortholog of human NAPSA (napsin A aspartic peptidase), REN (renin) and CTSD (cathepsin D); asp-8 is predicted to have aspartic-type endopeptidase activity, based on protein domain information.	109.59	58.04	45.79	34.68	34.13	1.48E-04
B0041.5	B0041.5 encodes a predicted transmembrane transporter that displays similarity to members of the F5 subfamily of solute carrier family 35 (SLC35F5) proteins that function as nucleotide sugar transporters; loss of B0041.5 activity via RNAi results in decreased intestinal dipeptide uptake and a slight reduction in fat droplet diameter compared to control animals; loss of B0041.5 activity has also been reported to affect axon guidance and fasciculation.	1205.46	937.27	1471.96	1416.15	1570.38	1.18E-04
B0218.7	B0218.7	170.62	79.18	69.76	51.48	47.00	9.14E-05
B0244.9	B0244.9	189.70	88.54	74.47	46.58	45.46	1.07E-05
B0252.5	B0252.5	95.94	52.81	40.90	30.77	35.17	5.88E-05
B0261.6	B0261.6	155.89	76.89	66.06	49.00	48.21	1.08E-04
B0273.1	B0273.1	88.41	63.93	45.55	32.78	34.50	1.28E-04
B0310.3	B0310.3	9.56	57.21	18.50	13.49	12.55	1.27E-05
B0379.2	B0379.2	33.10	18.97	13.26	10.65	10.83	2.58E-05
B0546.4	B0546.4 is an ortholog of human YPEL5 (yippee-like 5 (Drosophila)).	401.35	948.39	365.26	359.20	433.25	4.44E-05
bas-1	bas-1 encodes a serotonin- and dopamine-synthetic aromatic amino acid decarboxylase (AAADC) that is required for the synthesis of serotonin from 5-hydroxytryptophan in vivo; mutations of bas-1 impair the turning step in male mating, the migration of AVM, SDQR, ALM, and BDU neurons during development.	267.26	87.08	146.66	201.59	225.02	7.30E-06
best-13	best-13 is an ortholog of human BEST1 (bestrophin 1), BEST4 (bestrophin 4), BEST3 (bestrophin 3) and BEST2 (bestrophin 2).	68.83	97.96	136.08	122.97	108.72	1.51E-04
best-26	best-26 is an ortholog of human BEST1 (bestrophin 1), BEST4 (bestrophin 4), BEST3 (bestrophin 3) and BEST2 (bestrophin 2).	26.51	18.38	25.37	24.76	26.62	6.71E-05
btb-21	BTB (Broad/complex/Tramtrack/ Bric a brac) domain protein	14.82	49.17	16.45	15.26	16.99	8.14E-05
btb-9	BTB (Broad/complex/Tramtrack/ Bric a brac) domain protein	49.69	133.96	182.20	150.23	134.38	1.58E-05
C01F6.2	C01F6.2	41.82	26.17	26.86	25.48	24.78	1.68E-04
C01G10.9	C01G10.9 is an ortholog of human MRI1 (methylthioribose-1-phosphate isomerase 1).	139.62	113.23	317.48	398.13	305.92	4.60E-06
C01G12.3	C01G12.3	127.89	96.78	62.31	54.19	54.78	9.63E-05
C02F5.5	C02F5.5	252.47	133.99	99.49	53.25	57.30	2.12E-04
C03C10.2	C03C10.2 is an ortholog of human TTBK2 (tau tubulin kinase 2) and TTBK1 (tau tubulin kinase 1); C03C10.2 is predicted to have protein kinase activity and ATP binding activity, based on protein domain information.	19.43	14.75	11.90	11.78	11.12	4.07E-05
C04F12.7	C04F12.7	791.96	437.97	278.34	112.50	97.17	1.28E-04
C04G2.2	C04G2.2 is an ortholog of human TTBK2 (tau tubulin kinase 2) and TTBK1 (tau tubulin kinase 1); C04G2.2 is predicted to have protein kinase activity and ATP binding activity, based on protein domain information.	76.33	49.90	46.41	36.63	42.71	2.11E-04

C05C10.3	C05C10.3 is orthologous to the human gene 3-OXOACID COA TRANSFERASE (also called succinyl-CoA:3-ketoacid CoA transferase; OXCT; OMIM:245050), which when mutated leads to episodic ketoacidosis.	854.95	541.34	589.64	455.38	481.45	1.68E-04
C05C12.5	C05C12.5	250.55	124.42	97.18	56.97	58.78	1.11E-04
C06A8.6	C06A8.6 is an ortholog of human PPP1R7 (protein phosphatase 1, regulatory subunit 7).	125.50	57.01	39.68	27.38	23.81	1.94E-05
C06G8.3	C06G8.3 is an ortholog of human SLC39A2 (solute carrier family 39 (zinc transporter), member 2); C06G8.3 is predicted to have metal ion transmembrane transporter activity, based on protein domain information.	143.61	382.56	109.44	119.64	162.36	4.85E-05
C07A4.3	C07A4.3	45.19	99.21	39.29	29.29	28.15	6.90E-06
C08F11.10	C08F11.10	112.44	47.20	33.57	31.81	23.15	7.58E-05
C08F8.6	C08F8.6 is an ortholog of human TTBK2 (tau tubulin kinase 2) and TTBK1 (tau tubulin kinase 1); C08F8.6 is predicted to have protein kinase activity and ATP binding activity, based on protein domain information.	113.72	57.67	46.01	29.85	29.97	7.00E-06
C09D4.1	C09D4.1 is an ortholog of human FLVCR2 (feline leukemia virus subgroup C cellular receptor 2) and FLVCR1 (feline leukemia virus subgroup C cellular receptor 1).	13.61	37.88	16.61	14.57	15.20	4.60E-06
C09D4.2	C09D4.2	180.00	1069.80	787.18	577.56	492.89	1.73E-04
C09D4.3	C09D4.3 is an ortholog of human TTBK2 (tau tubulin kinase 2) and TTBK1 (tau tubulin kinase 1); C09D4.3 is predicted to have protein kinase activity and ATP binding activity, based on protein domain information.	135.37	54.01	46.33	32.51	32.38	1.98E-05
C09H5.7	C09H5.7 is an ortholog of human PPP1CC (protein phosphatase 1, catalytic subunit, gamma isozyme) and PPP1CA (protein phosphatase 1, catalytic subunit, alpha isozyme); C09H5.7 is predicted to have hydrolase activity, based on protein domain information.	54.96	27.67	30.82	24.04	23.04	5.61E-05
C10G11.8	C10G11.8 is an ortholog of human PSMC1 (proteasome (prosome, macropain) 26S subunit, ATPase, 1); C10G11.8 is predicted to have ATP binding activity and hydrolase activity, based on protein domain information.	372.90	160.85	130.89	72.59	61.40	9.65E-05
C10H11.7	Major sperm protein	60.16	37.63	24.90	17.61	19.71	1.24E-04
C12D8.9	C12D8.9	21.56	13.94	11.54	11.17	10.83	2.58E-04
C13C4.6	C13C4.6 is an ortholog of human MFSD7 (major facilitator superfamily domain containing 7).	49.62	215.78	34.88	40.08	49.79	2.50E-06
C14B1.3	This gene encodes a protein containing an F-box, a motif predicted to mediate protein-protein interactions either with homologs of yeast Skp-1p or with other proteins.	230.08	546.30	286.59	246.03	284.81	7.24E-05
C14C10.1	C14C10.1 is an ortholog of human SLC25A3 (solute carrier family 25 (mitochondrial carrier; phosphate carrier), member 3).	229.88	112.90	92.92	65.67	57.04	2.53E-04
C14C11.1 /// C18G1.9 ///ZC317.6	C14C11.1 /// C18G1.9 ///ZC317.6	155.66	82.16	63.65	38.87	34.44	1.78E-04
C14H10.1	C14H10.1 is an ortholog of human SLC39A13 (solute carrier family 39 (zinc transporter), member 13); C14H10.1 is predicted to have metal ion transmembrane transporter activity, based on protein domain information.	277.41	120.38	133.58	120.56	116.03	1.86E-05
C15B12.1	C15B12.1 is an ortholog of human PIPOX (pipecolic acid oxidase); C15B12.1 is predicted to have oxidoreductase activity, based on protein domain information.	70.83	222.40	199.90	176.86	139.97	1.78E-04
C15C6.2	C15C6.2	309.00	124.26	84.81	40.87	32.24	8.44E-05
C15C7.6	C15C7.6	24.16	16.58	19.90	19.42	18.51	2.46E-04
C16C8.18	C16C8.18 is an ortholog of human RMDN1 (regulator of microtubule dynamics 1), RMDN3 (regulator of microtubule dynamics 3) and RMDN2 (regulator of microtubule dynamics 2).	60.16	22.55	18.35	17.04	18.57	1.39E-05
C16D9.5	C16D9.5	14.17	9.03	9.32	8.76	8.90	9.00E-07
C17F4.7	C17F4.7	5944.03	8251.34	7095.58	8167.82	7892.94	6.14E-05
C17H11.6	C17H11.6 is an ortholog of human RNF19B (ring finger protein 19B) and RNF19A (ring finger protein 19A, RBR E3 ubiquitin protein ligase); C17H11.6 is predicted to have zinc ion binding activity, based on protein domain information.	61.09	122.51	76.34	85.86	85.92	2.22E-05

C17H12.3	C17H12.3 is an ortholog of human PTPN22 (protein tyrosine phosphatase, non-receptor type 22 (lymphoid)), PTPN18 (protein tyrosine phosphatase, non-receptor type 18 (brain-derived)) and PTPN12 (protein tyrosine phosphatase, non-receptor type 12); C17H12.3 is predicted to have protein tyrosine phosphatase activity, based on protein domain information.	157.35	78.27	58.45	40.67	38.71	2.10E-04
C18B12.2	C18B12.2 encodes a G-protein-coupled receptor (GPCR) that is a member of the secretin family (also known as family B or family 2) of GPCRs.	13.58	10.76	10.62	10.36	11.19	5.74E-05
C18C4.5	C18C4.5	35.89	61.57	57.86	56.19	46.44	1.20E-04
C18D4.8	C18D4.8	9.99	44.08	14.26	16.07	17.09	3.00E-07
C24A11.1	C24A11.1	75.65	36.29	31.73	24.72	19.37	1.58E-04
C25A8.2 /// R02D5.7	C25A8.2 /// R02D5.7	14.57	11.39	12.38	11.00	11.31	1.30E-04
C26C6.6	C26C6.6 is an ortholog of human LMO1 (LIM domain only 1 (rhombotin 1)), LMO3 (LIM domain only 3 (rhombotin-like 2)) and LMO2 (LIM domain only 2 (rhombotin-like 1)); C26C6.6 is predicted to have zinc ion binding activity, based on protein domain information.	15.54	12.60	11.27	9.14	9.50	9.53E-05
C26E1.2	C26E1.2	56.78	243.52	105.22	88.45	96.67	2.16E-05
C27D6.3	C27D6.3	340.37	149.08	90.70	48.72	37.50	7.56E-05
C28D4.5	C28D4.5	83.71	33.90	24.13	13.82	12.45	3.78E-05
C29F7.1	C29F7.1 is predicted to have transferase activity, transferring phosphorus-containing groups, based on protein domain information.	31.49	75.26	44.59	47.17	42.36	2.20E-04
C29F9.1	C29F9.1 is an ortholog of human ALS2CR12 (amyotrophic lateral sclerosis 2 (juvenile) chromosome region, candidate 12).	41.14	60.93	64.38	63.04	56.49	1.83E-04
C31C9.2	C31C9.2 is orthologous to the human gene PHOSPHOGLYCERATE DEHYDROGENASE (PHGDH; OMIM:606879), which when mutated leads to phosphoglycerate dehydrogenase deficiency.	127.78	111.62	186.84	215.59	235.02	2.58E-04
C31H1.1	C31H1.1	39.26	24.29	26.21	24.86	26.08	2.78E-05
C31H1.5	C31H1.5 is an ortholog of human PLA2G12B (phospholipase A2, group XIIB) and PLA2G12A (phospholipase A2, group XIIA); C31H1.5 is predicted to have phospholipase A2 activity and calcium ion binding activity, based on protein domain information.	186.31	78.95	53.33	26.50	24.05	4.91E-05
C32D5.12	C32D5.12 is predicted to have 3-beta-hydroxy-delta5-steroid dehydrogenase activity, based on protein domain information.	37.55	181.52	132.83	111.49	73.72	1.72E-04
C32E8.4	C32E8.4	288.35	149.14	121.16	81.40	86.98	2.23E-04
C32F10.4	C32F10.4	141.35	1523.06	718.27	455.66	414.87	3.13E-05
C33H5.13	C33H5.13 is involved in innate immune response.	272.07	1162.01	1018.03	878.46	650.34	3.26E-05
C34B2.3	C34B2.3 is an ortholog of human TTBK2 (tau tubulin kinase 2) and TTBK1 (tau tubulin kinase 1); C34B2.3 is predicted to have protein kinase activity and ATP binding activity, based on protein domain information.	51.84	30.77	30.79	30.04	28.22	7.12E-05
C34C6.7	C34C6.7	25.93	587.41	348.67	225.94	134.65	2.04E-05
C34D1.4	C34D1.4	41.26	79.88	33.06	35.95	33.45	8.30E-06
C34D10.2	C34D10.2 is an ortholog of human UNK (unkempt family zinc finger) and UNKL (unkempt family zinc finger-like); C34D10.2 is predicted to have metal ion binding activity, based on protein domain information.	31.45	149.22	54.33	38.33	33.51	8.41E-05
C34D4.3	C34D4.3	334.55	213.27	109.78	45.85	34.49	1.69E-04
C34F11.2	C34F11.2	105.28	51.48	29.64	16.69	13.85	9.96E-05
C35A11.4	C35A11.4 is an ortholog of human SLC2A9 (solute carrier family 2 (facilitated glucose transporter), member 9), SLC2A11 (solute carrier family 2 (facilitated glucose transporter), member 11), SLC2A7 (solute carrier family 2 (facilitated glucose transporter), member 7) and SLC2A5 (solute carrier family 2 (facilitated glucose/fructose transporter), member 5); C35A11.4 is predicted to have substrate-specific transmembrane transporter activity, based on protein domain information.	106.70	46.53	171.87	192.45	152.31	7.10E-06
C35C5.8	C35C5.8	22.65	233.28	80.86	38.99	31.11	5.06E-05
C38C10.3	C38C10.3	83.24	45.87	45.74	36.57	39.54	1.80E-04

C38C3.4	C38C3.4 is an ortholog of human TTBK2 (tau tubulin kinase 2) and TTBK1 (tau tubulin kinase 1); C38C3.4 is predicted to have protein kinase activity and ATP binding activity, based on protein domain information.	19.77	58.00	30.58	22.58	21.83	2.73E-05
C39H7.1	C39H7.1 is an ortholog of human TTBK2 (tau tubulin kinase 2) and TTBK1 (tau tubulin kinase 1); C39H7.1 is predicted to have protein kinase activity and ATP binding activity, based on protein domain information.	248.45	108.95	81.08	41.90	30.73	2.42E-04
C39H7.1 /// Y38H8A.3	C39H7.1 is an ortholog of human TTBK2 (tau tubulin kinase 2) and TTBK1 (tau tubulin kinase 1); C39H7.1 is predicted to have protein kinase activity and ATP binding activity, based on protein domain information. Y38H8A.3 is an ortholog of human TTBK2 (tau tubulin kinase 2) and TTBK1 (tau tubulin kinase 1); Y38H8A.3 is predicted to have protein kinase activity and ATP binding activity, based on protein domain information.	590.53	248.56	177.45	87.64	61.97	1.16E-04
C41G11.1	C41G11.1	73.70	654.45	539.10	512.21	420.97	5.52E-05
C42D4.2	C42D4.2 is an ortholog of human CES4A (carboxylesterase 4A), CEL (carboxyl ester lipase), CES1 (carboxylesterase 1), CES5A (carboxylesterase 5A) and CES3 (carboxylesterase 3).	52.80	14.27	14.93	36.13	41.74	1.04E-04
C43E11.5 /// C50F2.5	C43E11.5 is an ortholog of human PTPN2 (protein tyrosine phosphatase, non-receptor type 2) and PTPN1 (protein tyrosine phosphatase, non-receptor type 1); C43E11.5 is predicted to have protein tyrosine phosphatase activity, based on protein domain information. C50F2.5 is an ortholog of human PTPN2 (protein tyrosine phosphatase, non-receptor type 2) and PTPN1 (protein tyrosine phosphatase, non-receptor type 1); C50F2.5 is predicted to have protein tyrosine phosphatase activity, based on protein domain information.	147.21	74.69	60.01	39.15	35.87	5.88E-05
C44C1.5	C44C1.5 is an ortholog of human ABHD1 (abhydrolase domain containing 1) and ABHD3 (abhydrolase domain containing 3).	530.39	50.51	783.32	895.53	754.81	5.00E-07
C44H9.6	C44H9.6	24.34	38.23	57.79	74.30	53.83	1.50E-05
C45E5.1	C45E5.1 is an ortholog of human PDXP (pyridoxal (pyridoxine, vitamin B6) phosphatase) and PGP (phosphoglycolate phosphatase); C45E5.1 is predicted to have phosphatase activity, based on protein domain information.	70.54	32.63	282.27	191.15	163.52	1.33E-04
C47A4.3	C47A4.3 is an ortholog of human PPP1CC (protein phosphatase 1, catalytic subunit, gamma isozyme) and PPP1CA (protein phosphatase 1, catalytic subunit, alpha isozyme); C47A4.3 is predicted to have hydrolase activity, based on protein domain information.	36.24	20.58	18.98	16.54	15.38	4.25E-05
C47E12.11	C47E12.11	38.42	20.10	16.72	12.95	11.57	8.95E-05
C48B6.3	C48B6.3 is an ortholog of human C16orf72 (chromosome 16 open reading frame 72).	125.04	93.24	95.73	89.10	76.85	7.65E-05
C49A9.2	C49A9.2	42.23	53.89	74.08	75.82	78.27	4.81E-05
C49A9.4	C49A9.4 is orthologous to the human gene CYSTATIN B (STEFIN B) (CSTB; OMIM:601145), which when mutated leads to disease.	91.27	76.56	172.72	174.07	167.44	1.21E-04
C50A2.3	C50A2.3	73.96	31.19	272.53	149.79	106.57	6.16E-05
C50D2.3	C50D2.3	59.17	34.61	24.16	14.05	11.41	1.60E-05
C52B11.5	C52B11.5 is an ortholog of human RAB20 (RAB20, member RAS oncogene family); C52B11.5 is predicted to have GTP binding activity, based on protein domain information.	145.60	166.68	266.82	428.00	464.48	5.40E-05
C52D10.1	dead and merged to C52D10.3	151.70	54.11	214.26	317.66	219.41	6.68E-05
C52E2.4	C52E2.4	30.51	81.30	30.78	30.32	30.15	3.10E-06
C53B7.2	C53B7.2 encodes a putative secreted TIL-domain protease inhibitor paralogous to SWM-1, ISL-1, and the products of 11 other C. elegans genes; C53B7.2 and its relatives are collectively similar to other TIL-domain protease inhibitors from nematodes, insects, and vertebrates; C53B7.2 has no obvious function in mass RNAi assays.	455.58	1524.70	1325.61	1095.80	1078.74	3.43E-05
C53B7.3	C53B7.3	191.67	1719.85	1437.49	1195.95	989.94	3.50E-06
C54D10.10	C54D10.10 is an ortholog of human TFPI2 (tissue factor pathway inhibitor 2) and TFPI (tissue factor pathway inhibitor (lipoprotein-associated coagulation inhibitor)); C54D10.10 is predicted to have serine-type endopeptidase inhibitor activity, based on protein domain information.	31.73	15.76	14.86	14.20	15.71	1.61E-05
C54G4.3	C54G4.3	74.37	34.54	27.30	20.95	19.96	7.47E-05

C55A1.6	C55A1.6	22.08	70.61	80.20	83.41	69.97	1.24E-04
C55A6.4	C55A6.4 is an ortholog of human RDH8 (retinol dehydrogenase 8 (all-trans)) and DHRS1 (dehydrogenase/reductase (SDR family) member 1); C55A6.4 is predicted to have oxidoreductase activity, based on protein domain information.	221.65	59.01	54.60	44.58	58.94	3.00E-06
C55B7.3	C55B7.3 is an ortholog of human PTPN22 (protein tyrosine phosphatase, non-receptor type 22 (lymphoid)), PTPN18 (protein tyrosine phosphatase, non-receptor type 18 (brain-derived)) and PTPN12 (protein tyrosine phosphatase, non-receptor type 12); C55B7.3 is predicted to have protein tyrosine phosphatase activity, based on protein domain information.	98.45	41.94	29.79	14.96	16.30	5.83E-05
C55C2.4	C55C2.4	23.57	17.00	17.85	15.55	17.37	2.52E-04
C55C3.4	C55C3.4 is an ortholog of human ABL2 (ABL proto-oncogene 2, non-receptor tyrosine kinase) and ABL1 (ABL proto-oncogene 1, non-receptor tyrosine kinase); C55C3.4 is predicted to have protein tyrosine kinase activity, based on protein domain information.	79.00	45.47	38.49	32.46	30.72	1.70E-04
cah-5	cah-5 encodes a member of the carbonic anhydrase family.	232.83	88.82	338.91	408.01	376.52	1.53E-05
cal-2	cal-2 encodes a calmodulin homolog required for embryonic development or viability; CAL-2 is closely similar to its paralogs CAL-1, CAL-3, CAL-4 and CMD-1.	32.76	104.85	48.97	48.04	48.01	1.70E-04
catp-4	catp-4 is an ortholog of human ATP1A3 (ATPase, Na+/K+ transporting, alpha 3 polypeptide), ATP1A4 (ATPase, Na+/K+ transporting, alpha 4 polypeptide), ATP1A2 (ATPase, Na+/K+ transporting, alpha 2 polypeptide), ATP4A (ATPase, H+/K+ exchanging, alpha polypeptide) and ATP1A1 (ATPase, Na+/K+ transporting, alpha 1 polypeptide); catp-4 is predicted to have sodium:potassium-exchanging ATPase activity, ATP binding activity, and metal ion binding activity, based on protein domain information.	140.20	50.22	46.18	36.98	33.35	5.89E-05
cbl-1	cbl-1 encodes a putative cystathionine gamma-lyase orthologous to human CTH (OMIM:607657, mutated in cystathioninuria), and paralogous to CTH-1 and CTH-2; CBL-1, unlike its paralog CTH-2, does not inhibit DHC-1 in vivo.	149.09	37.24	186.36	276.34	233.99	1.57E-05
cbs-1	cbs-1 encodes an ortholog of human CYSTATHIONINE-BETA-SYNTHASE (CBS), which when mutated leads to homocystinuria (OMIM:236200).	155.05	54.00	183.74	193.79	150.35	2.11E-05
cdf-1	cdf-1 encodes a protein similar to members of the cation diffusion facilitator (CDF) family of transmembrane proteins and is most similar to vertebrate ZnT-1; two other elegans CDF proteins, cdf-2 and sur-7 have also been identified; CDF proteins regulate the transport of heavy metal ions like zinc; cdf-1 positively regulates Ras-mediated signaling during vulval development, most likely by promoting the efflux of zinc ions, as cytosolic zinc inhibits Ras signaling; cdf-1 mutants are hypersensitive to zinc but not other heavy metals tested, indicating that cdf-1 is specifically involved in regulating zinc metabolism; cdf-1 is expressed in the vulval muscles, the intestinal cells, and in the vulval precursor cells and localizes to the plasma membrane.	83.08	71.09	132.77	122.02	133.28	1.51E-04
ceh-20	ceh-20 encodes one of three C. elegans homeodomain proteins (CEH-20, CEH-40, and CEH-60) homologous to Extradenticle (Exd/Pbx); together with ceh-40 and unc-62, ceh-20 activity is required for embryonic viability; ceh-20 is also required as a cofactor for LIN-39- and MAB-5-dependent postembryonic mesoderm patterning; in addition, ceh-20 is required for regulating post-embryonic migrations of the Q neuroblast descendants and for regulating vulval development; a CEH-20::GFP fusion protein is expressed in embryos and postembryonically in many cell types including the Q, P, and V cells and their descendants; CEH-20 localizes to the nucleus.	100.23	264.86	157.24	220.12	160.70	5.04E-05
cex-2	cex-2 is an ortholog of human RCN2 (reticulocalbin 2, EF-hand calcium binding domain), RCN3 (reticulocalbin 3, EF-hand calcium binding domain), RCN1 (reticulocalbin 1, EF-hand calcium binding domain) and CALU (calumenin); cex-2 is predicted to have calcium ion binding activity, based on protein domain information.	54.99	114.48	104.42	55.72	64.45	2.56E-04
chil-23	chil-23 is an ortholog of human OVGP1 (oviductal glycoprotein 1), CHI3L1 (chitinase 3-like 1 (cartilage glycoprotein-39)), CHI3L2 (chitinase 3-like 2) and CHIT1 (chitinase 1 (chitotriosidase)); chil-23 is predicted to have chitinase activity, based on protein domain information.	11.18	72.00	12.83	14.15	12.67	5.00E-07
cht-3	cht-3 is an ortholog of human OVGP1 (oviductal glycoprotein 1), CHI3L1 (chitinase 3-like 1 (cartilage glycoprotein-39)), CHI3L2 (chitinase 3-like 2) and CHIT1 (chitinase 1 (chitotriosidase)); cht-3 is predicted to have chitinase activity, based on protein domain information.	889.59	2050.87	1749.39	1698.03	1584.01	6.08E-05

cima-1	circuit maintenance abnormal: cima-1 encodes a membrane solute transporter that is a member of the SLC17 family of transporters; CIMA-1 functions postembryonially in epidermal cells to maintain synaptic contacts established during embryogenesis; specifically, CIMA-1 negatively regulates levels of the EGL-15(5A)/FGF receptor isoform to correctly position glia and control epidermal-glia cell adhesion; cima-1 expression begins during embryogenesis and continues through adulthood; in adults, CIMA-1 is mainly found in the epidermis, with some expression also seen in the intestine; a CIMA-1 reporter fusion localizes to lysosomes.	42.30	26.35	62.97	44.26	40.68	1.95E-04
ckc-1	ckc-1 encodes a putative ethanolamine or choline kinase, with somewhat more sequence similarity to ethanolamine kinases; CKC-1 is the sole member of a 'C' group of choline/ethanolamine kinases.	178.98	45.06	187.99	232.99	206.55	5.80E-06
cki-1	cki-1 encodes a homolog of the mammalian cyclin-dependent kinase inhibitor p27/KIP1 that is required for the arrest of cell division in larval blast lineages, dauer larvae and starved L1 larvae; excess CKI-1 expression prematurely stops cell division while cki-1(RNAi) induces extra cell divisions, indicating that CKI-1 quantitatively regulates the amount of mitosis in postembryonic worms.	49.80	249.22	190.41	200.95	141.46	3.15E-05
clec-151	clec-151	28.28	15.80	12.05	9.92	10.16	9.68E-05
clec-222	C-type LECtin	92.72	374.91	380.64	332.13	344.96	1.85E-04
clec-54	clec-54 is an ortholog of human CDCP2 (CUB domain containing protein 2).	70.95	206.56	191.55	152.33	125.78	2.36E-04
cnb-1	cnb-1 encodes an ortholog of calcineurin B, the regulatory subunit of the protein phosphatase 2B with four EF-hand motifs for calcium binding, that binds TAX-6 (a calcineurin A ortholog) in a calcium-dependent manner; CNB-1 binds calcium, enhances the phosphatase activity of TAX-6 in vitro, promotes transcription of rcn-1, and is required for normal cuticle formation, sperm morphology, and brood size; cnb-1 null mutants exhibit an extended lifespan which is characterized by enhanced autophagy and dependent upon the presence of functional bec-1 and atg-7.	147.84	353.03	182.53	171.25	169.26	6.20E-06
coel-1	tubulin folding COfactor E-Like protein: coel-1 encodes, by alternative splicing, two isoforms of a putative tubulin-destabilizing protein orthologous to human TBCEL (LRRC35; OMIM:610451), and paralogous to K07H8.1 and human TBCE (OMIM:604934, mutated in HRD); coel-1(tm2136) homozygotes are viable, and COEL-1 has no obvious function in mass RNAi assays.	13.14	49.63	14.62	13.59	15.42	1.00E-07
col-10	col-10	4342.80	2495.87	1682.57	1605.85	1526.51	1.17E-04
col-103	col-103	2176.99	455.75	400.78	321.75	369.81	4.77E-05
col-110	col-110	105.10	43.80	49.93	45.51	48.31	1.37E-05
col-117 /// col-3	col-117 /// col-3	10353.20	3358.35	2701.61	2655.41	2513.34	1.92E-05
col-12	col-12	7040.41	1694.70	994.10	915.19	939.30	4.23E-05
col-126 /// col-127	col-126 /// col-127	226.06	55.84	59.95	49.89	56.34	7.93E-05
col-129	col-129	7500.06	1210.99	755.10	460.03	456.43	2.10E-04
col-133	col-133	7536.23	2173.91	1494.54	1317.35	1431.62	4.38E-05
col-137	col-137	44.77	16.19	15.71	14.87	16.23	1.02E-04
col-141	col-141	135.67	51.72	40.71	39.26	47.34	1.77E-04
col-142	col-142	2309.62	459.23	192.32	146.17	193.54	9.00E-07
col-146	col-146	2633.13	1200.49	909.26	873.46	923.46	1.05E-04
col-147	col-147	3927.99	782.52	613.94	628.41	659.62	2.00E-07
col-149	col-149	536.60	48.90	56.16	56.18	54.34	1.40E-06
col-150	col-150	165.72	62.63	46.77	50.69	53.27	1.14E-05
col-161 /// col-162	col-161 /// col-162	216.14	48.49	45.11	45.84	42.45	1.98E-05
col-164	col-164	81.27	43.40	37.28	40.68	40.92	7.06E-05

col-167 /// col-168 /// col-170	col-167 /// col-168 /// col-170	3582.41	1392.39	1049.69	895.06	969.91	2.06E-04
col-17	col-17	5178.26	2111.81	1680.24	1548.15	1636.81	9.78E-05
col-173	col-173	71.29	47.26	44.04	38.61	40.47	1.97E-04
col-180	col-180	252.12	59.69	62.16	58.76	60.31	2.20E-06
col-19	col-19	3245.52	1864.05	1198.74	883.91	871.96	1.65E-04
col-2	col-2	127.59	66.89	60.75	59.18	61.08	3.57E-05
col-34	col-34	1048.98	566.54	456.88	419.35	380.87	1.44E-04
col-39	COLlagen	886.17	63.16	44.01	38.39	49.14	1.00E-07
col-43	col-43	4046.65	2131.97	1570.76	1772.65	1524.62	5.77E-05
col-53	col-53	88.76	46.68	39.36	40.49	41.28	1.77E-04
col-62 /// col-7	col-62 /// col-7	1149.76	80.34	64.81	57.61	52.69	2.51E-05
col-66	col-66	170.07	100.25	89.79	92.01	85.45	2.52E-04
col-71	col-71	102.53	40.66	44.21	39.30	39.83	5.90E-05
comp-1	comp-1 is predicted to have transferase activity, transferring phosphorus-containing groups, based on protein domain information.	108.84	58.69	49.52	42.61	48.39	1.06E-04
comt-4	comt-4 is an ortholog of human COMTD1 (catechol-O-methyltransferase domain containing 1); comt-4 is predicted to have O-methyltransferase activity, based on protein domain information.	61.17	111.18	425.62	407.30	286.69	9.05E-05
coq-1	coq-1 encodes a putative hexaprenyl pyrophosphate synthetase, orthologous to S. cerevisiae COQ1; COQ-1 is required for ubiquinone (coenzyme Q9) biosynthesis and for normally short lifespan; coq-1 mutants have slowed pharyngeal pumping, and eventually arrest as paralyzed larvae before dying; coq-1(RNAi) animals have reduced levels of coenzyme Q9 and superoxide, and have abnormally long lifespans; coq-1 mutants are not rescued by dietary coenzyme Q.	363.65	284.25	535.19	456.48	407.01	9.00E-07
crm-1	crm-1 encodes a predicted transmembrane protein with multiple extracellular cysteine-rich (CR) domains that is the C. elegans ortholog of vertebrate CRIM1 (Cysteine-RIch Motor neuron 1); genetic analyses indicate that CRM-1 functions non-autonomously to positively regulate DBL-1/BMP-mediated signaling in the pathway that controls body size determination; in addition, loss of crm-1 activity via RNAi has been reported to result in animals that are sick, have molting defects, and show maternal sterility; a crm-1a::gfp reporter fusion is expressed in ventral cord neurons, neurons in the pharyngeal region, and male tail neurons, while a crm-1b::gfp reporter is seen in the pharynx and posterior intestine.	67.54	199.68	126.55	95.37	87.84	1.64E-04
cutl-16	CUTiclin-Like	12.97	35.91	23.98	25.35	20.82	5.07E-05
cyn-2	cyn-2 is a predicted member of the cytosolic Cyclosporin A-binding cyclophilin family that is functional when expressed in E. coli.	102.24	60.00	47.21	34.29	32.16	4.72E-05
cyp-13A5	cyp-13A5 encodes one of ~80 C. elegans cytochrome P450s: membrane-associated, heme-containing NADPH-dependent monooxygenases that catalyze the oxidative metabolism of a variety of exogenous compounds and endogenous substrates; loss of cyp-13A5 activity in large-scale RNAi screens results in uncoordinated locomotion, decreased and/or slow growth, and cadmium hypersensitivity.	10.72	79.44	19.43	11.02	12.31	1.37E-04
cyp-29A3	cyp-29A3 is an ortholog of human CYP19A1 (cytochrome P450 19A1), CYP4V2 (cytochrome P450 4V2), CYP4B1 (cytochrome P450 4B1), CYP4F3 (cytochrome P450 4F3) and CYP4A22 (cytochrome P450 4A22); cyp-29A3 is predicted to have iron ion binding activity, oxidoreductase activity, acting on paired donors, with incorporation or reduction of molecular oxygen, and heme binding activity, based on protein domain information.	43.67	28.36	31.49	33.27	33.45	9.39E-05
cyp-32A1	cyp-32A1 is predicted to have iron ion binding activity, oxidoreductase activity, acting on paired donors, with incorporation or reduction of molecular oxygen, and heme binding activity, based on protein domain information.	18.33	38.08	19.89	15.12	15.82	2.13E-04

cyp-32B1	cyp-32B1 is predicted to have iron ion binding activity, oxidoreductase activity, acting on paired donors, with incorporation or reduction of molecular oxygen, and heme binding activity, based on protein domain information.	40.87	271.08	142.11	89.80	63.97	7.81E-05
cyp-33C4	cyp-33C4 is an ortholog of human CYP2D6 (cytochrome P450 2D6), CYP2J2 (cytochrome P450 2J2), CYP2R1 (cytochrome P450 2R1), CYP2E1 (cytochrome P450 2E1) and CYP2C8 (cytochrome P450 2C8); cyp-33C4 is predicted to have iron ion binding activity, oxidoreductase activity, acting on paired donors, with incorporation or reduction of molecular oxygen, and heme binding activity, based on protein domain information.	29.95	144.37	42.89	35.67	42.97	4.70E-06
cyp-36A1	cyp-36A1 is an ortholog of human CYP2C8 (cytochrome P450 2C8), CYP2C18 (cytochrome P450 2C18), CYP2E1 (cytochrome P450 2E1), CYP2U1 (cytochrome P450 2U1); cyp-36A1 is predicted to have iron ion binding activity, oxidoreductase activity, acting on paired donors, with incorporation or reduction of molecular oxygen, and heme binding activity, based on protein domain information.	24.20	14.52	59.41	65.99	47.46	1.11E-05
cysl-2	cysl-2 encodes a homolog of sulfhydrylases/cysteine synthases.	137.27	81.24	961.39	636.37	393.73	2.90E-06
cysl-3	cysl-3 is an ortholog of human CBS (cystathionine-beta-synthase); cysl-3 is involved in cysteine biosynthetic process from serine; cysl-3 exhibits cysteine synthase activity.	316.66	180.11	428.60	547.84	664.26	8.10E-06
D1025.2	gcsh-1 is an ortholog of human GCSH (glycine cleavage system protein H (aminomethyl carrier)).	199.94	20.42	232.39	270.78	233.43	1.64E-05
D1046.5	tpra-1 is an ortholog of human TPRA1 (transmembrane protein, adipocyte associated 1).	51.96	175.59	118.00	110.76	111.02	2.21E-04
D1081.4	D1081.4 is predicted to have GTP binding activity, based on protein domain information.	98.84	49.94	42.61	32.85	30.62	5.70E-06
D1081.5	D1081.5	53.05	25.01	21.62	17.79	16.63	3.63E-05
D1086.1	D1086.1	50.23	212.78	221.69	157.00	153.36	4.30E-05
D1086.2	D1086.2	13.11	104.43	107.64	70.32	57.40	1.49E-04
D2005.6	D2005.6	35.36	25.30	27.72	28.87	30.62	2.73E-05
D2062.6	D2062.6	562.14	339.64	198.80	108.19	77.06	4.48E-05
D2062.7	D2062.7	402.78	225.76	145.87	89.92	80.96	1.18E-04
daf-36	daf-36	95.39	50.10	402.35	363.60	277.53	1.00E-07
dao-2	dao-2 encodes a (putatively) secreted protein with a DB module; dao-2 is down-regulated in daf-2 mutant adults by comparison with normal adults, and thus may be involved in dauer formation. M03A1.8 is an ortholog of human FRRS1 (ferric-chelate reductase 1).	130.71	691.63	439.22	637.32	420.73	4.64E-05
dct-1	dct-1 encodes a protein with similarity to the mammalian BNIP3 proteins that interact with Bcl-2 and the Adenovirus E1B 19kDa protein and that have been shown to have pro-apoptotic activity; loss of dct-1 activity via RNAi in daf-2 mutants and in gld-1; daf-2 doubly mutant animals indicates that dct-1 can function to regulate both lifespan and tumor cell proliferation; when expressed in mammalian cells, DCT-1 can: 1) induce delayed apoptosis and increase apoptosis when co-expressed with CED-3 (perhaps by increasing proteolyic processing of CED-3), 2) physically interact with CED-9 or the CED-3 prodomain when co-expressed, and 3) physically interact with both CED-9 and CED-3 when all three are co-expressed; chromatin immunoprecipitation experiments have demonstrated that the dct-1 promoter is bound in vivo by the forkhead transcription factor DAF-16; expression of DCT-1 in mammalian cells suggests that, like its mammalian orthologs, DCT-1 localizes to mitochondria via a predicted C-terminal transmembrane domain.	112.41	429.75	104.12	121.98	125.44	2.30E-06
decr-1.1	decr-1.1 DiEnoyl-Co A reductase, mitochondria	198.41	20.10	18.01	15.17	15.82	1.00E-07
decr-1.2	decr-1.2 is an ortholog of human DECR1 (2,4-dienoyl CoA reductase 1, mitochondrial).	43.99	23.90	18.27	16.10	15.73	6.66E-05
decr-1.3	decr-1.3 is an ortholog of human DECR1 (2,4-dienoyl CoA reductase 1, mitochondrial).	42.84	25.02	21.99	19.60	21.44	7.50E-06
dgk-2	dgk-2 encodes a putative diacylglycerol kinase.	125.31	224.95	114.97	130.44	151.24	3.42E-05
DH11.2	DH11.2	70.46	125.49	26.86	34.26	35.69	6.12E-05
dhhc-10	dhhc-10 is an ortholog of human ZDHHC22 (zinc finger, DHHC-type containing 22); dhhc-10 is predicted to have zinc ion binding activity, based on protein domain information.	124.40	281.99	148.90	162.10	175.79	9.44E-05

dlc-6	dlc-6 encodes a putative dynein light chain 1; DLC-6 is orthologous to human DYNLL1 (OMIM:601562) and DYNLL2 (OMIM:608942), but much more divergent from them than its paralog DLC-1; DLC-6's other paralogs are DLC-2/-5; DLC-6 has no obvious function in mass RNAi assays.	24.97	14.61	13.82	12.69	12.66	1.97E-04
dnpp-1	dnpp-1 is an ortholog of human DNPEP (aspartyl aminopeptidase); dnpp-1 is predicted to have aminopeptidase activity and zinc ion binding activity, based on protein domain information.	1844.07	1361.82	3868.13	3891.44	3168.63	5.12E-05
dpy-9	dpy-9 encodes a cuticular collagen family member with similarity to human collagen alpha 5, type IV, and affects body length.	98.47	66.79	60.02	59.00	64.86	4.87E-05
dur-1	The dur-1 ('Dauer UpRegulated') gene encodes proteins (DUR-1A and DUR-1C) that are predicted to be hydrophilic and heat-resistant, and that might participate in anhydrobiosis; expression of at least one dur-1 transcript is upregulated in dauer larvae and other transcripts may be specifically expressed in dauer larvae.	22.82	30.66	21.26	22.06	23.57	9.99E-05
E04F6.6	E04F6.6	35.12	23.67	23.75	25.13	23.62	1.07E-05
elo-9	The elo-9 gene encodes a paralog of elo-1 and elo-2, each of which encodes a polyunsaturated fatty acid (PUFA) elongase; elo-9 has no known function in vivo.	34.54	21.82	43.08	52.70	46.69	2.36E-04
elt-7	elt-7 encodes a GATA-type transcription factor; during embryonic development, ELT-7 functions together with ELT-2 in the endoderm/gut to regulate the transition from cell fate specification to differentiation; expression of ELT-7 is sufficient to induce gut-specific differentiation; an elt-7::gfp promoter fusion is expressed in the gut lineage beginning at the 2E-cell stage of gut development and continuing through adulthood; elt-7 expression is initiated through the combined action of the END-1 and END-3 transcription factors.	126.30	511.86	232.41	246.75	265.08	2.20E-05
ets-4	ets-4 is an ortholog of human SPDEF (SAM pointed domain containing ETS transcription factor); ets-4 is predicted to have sequence-specific DNA binding transcription factor activity and sequence-specific DNA binding activity, based on protein domain information; ets-4 is localized to the nucleus.	183.30	539.05	248.81	386.24	381.10	1.90E-06
F02E9.3	F02E9.3	58.15	38.94	38.97	31.64	31.10	2.49E-04
F02E9.5	F02E9.5 is an ortholog of human OSER1 (oxidative stress responsive serine-rich 1).	258.85	519.44	413.65	371.16	348.07	4.57E-05
F07A5.2	F07A5.2	434.87	206.20	141.31	67.34	56.54	9.75E-05
F07F6.1	F07F6.1 is predicted to have nucleotide binding activity, based on protein domain information.	64.43	32.91	29.21	23.74	23.49	1.44E-04
F08G2.4	F08G2.4	155.45	467.49	217.46	194.22	193.65	1.50E-04
F08H9.3	F08H9.3 encodes a heat shock protein (HSP) of the HSP16 class; unlike most HSP16s in C. elegans, it is specifically expressed in a particular tissue (the larval pharynx and the anterior adult pharynx), and its expression is not strongly induced by heat shock; however, F08H9.3 expression is increased by heat shock (with adult expression in the full pharynx) and probably aids heat shock resistance, since F08H9.3(RNAi) animals have sensitivity to heat shock that is sporadically higher than normal; in general, HSP16 proteins are thought to act as passive ligands for unfolded proteins that keep them safe from aggregation until the proteins can be refolded by a large (ATP-consuming) HSP.	9.46	32.63	9.66	9.89	9.99	1.00E-07
F09B12.3	F09B12.3 is an ortholog of human PLBD2 (phospholipase B domain containing 2).	285.55	107.60	636.99	777.20	572.62	5.98E-05
F09C12.8	F09C12.8 encodes an ortholog, predicted to be catalytically inactive, of Bombyx mori ecdysteroid phosphate phosphatase (EPP), human STS1 (OMIM:609201) and human UBASH3A (OMIM:605736); F09C12.8 is also paralogous to C. elegans C52E4.7, F53B6.7, F55A11.11, and T07F12.1.	210.95	100.58	84.99	73.34	73.39	4.68E-05
F09E5.3	F09E5.3 is an ortholog of human DERA (deoxyribose-phosphate aldolase (putative)); F09E5.3 is predicted to have deoxyribose-phosphate aldolase activity, based on protein domain information.	1017.96	801.87	1000.88	1140.99	1213.88	2.79E-05
F10D11.3	F10D11.3	25.86	15.92	14.66	11.53	10.56	1.02E-04
F10G8.8	F10G8.8 is an ortholog of human TRIOBP (TRIO and F-actin binding protein) and MPRIP (myosin phosphatase Rho interacting protein).	15.02	34.60	23.02	26.94	20.48	1.74E-04
F11G11.4	F11G11.4	85.77	49.61	44.81	28.82	31.44	2.01E-04
F13A7.1	Major sperm protein	279.58	146.23	113.75	98.92	94.45	3.94E-05

F13E9.11	F13E9.11	23.30	311.07	109.11	213.03	104.92	1.06E-04
F16C3.2	F16C3.2	21.08	92.54	19.96	18.54	19.26	3.40E-06
F17C11.4	F17C11.4	193.80	1254.40	169.04	266.28	293.63	2.44E-05
F17C8.7 /// R07E5.15 /// T23F11.2	F17C8.7 /// R07E5.15 /// T23F11.2	287.46	120.66	89.21	51.17	39.39	2.26E-04
F17E9.5	F17E9.5 encodes a novel protein conserved amongst nematodes.	304.80	133.92	86.14	43.20	30.59	1.82E-04
F17H10.3	snx-17 is an ortholog of human SNX31 (sorting nexin 31) and SNX17 (sorting nexin 17); snx-17 is predicted to have phosphatidylinositol binding activity, based on protein domain information.	55.08	101.12	85.23	91.05	74.06	4.46E-05
F18C5.10	F18C5.10	259.02	877.44	301.04	286.98	288.94	6.42E-05
F18G5.6	F18G5.6 is involved in innate immune response.	63.03	933.68	281.67	152.48	147.82	1.80E-06
F19B2.5	F19B2.5 is predicted to have DNA binding activity and ATP binding activity, based on protein domain information.	124.93	36.21	569.09	364.66	318.62	1.88E-04
F20C5.6	F20C5.6	15.80	54.58	26.76	43.13	26.71	8.17E-05
F21A3.2	F21A3.2	81.77	578.54	324.66	236.70	236.75	1.00E-06
F21A3.3	F21A3.3 encodes a putatively secreted protein, with an N-terminal EGF-liked domain and a DUF1794 domain, that is homologous to human THAP4/CGI-36; F21A3.3 is a paralog of MAB-7, C15F1.1, C28H8.5, C52G5.2, T28H10.2, and a domain in Y46G5A.29.	56.13	24.76	26.94	29.39	31.42	1.59E-04
F21D5.3	F21D5.3 is predicted to have copper ion binding activity and oxidoreductase activity, based on protein domain information.	29.77	34.81	169.75	136.81	86.70	1.81E-04
F21F8.2	Pseudogene	10.95	23.80	12.38	12.34	14.99	1.37E-05
F22E5.1	F22E5.1	141.91	55.74	112.18	162.67	171.82	8.19E-05
F22H10.3	F22H10.3	4768.34	7215.67	8018.07	8917.80	8204.02	2.29E-04
F25B3.4	F25B3.4 is an ortholog of human PPP1CB (protein phosphatase 1, catalytic subunit, beta isozyme); F25B3.4 is predicted to have hydrolase activity, based on protein domain information.	27.48	20.85	18.24	16.32	16.02	1.08E-04
F26F12.3	F26F12.3	42.70	188.60	38.90	47.05	40.36	1.00E-07
F27C8.5	F27C8.5	34.27	20.87	17.26	16.85	14.79	1.44E-04
F31D4.8	F31D4.8	107.20	54.09	241.36	286.72	241.28	6.30E-06
F31E8.5	F31E8.5	63.03	30.27	24.52	21.12	20.02	2.20E-06
F32B6.4	F32B6.4	268.71	112.15	70.15	42.38	32.35	1.25E-04
F33D11.2	F33D11.2	21.24	12.96	12.53	13.25	12.84	9.23E-05
F33D11.7	F33D11.7 is an ortholog of human TTBK2 (tau tubulin kinase 2) and TTBK1 (tau tubulin kinase 1); F33D11.7 is predicted to have protein kinase activity and ATP binding activity, based on protein domain information.	28.05	17.58	19.15	18.01	17.57	1.40E-04
F34H10.3	F34H10.3	149.07	467.09	112.73	114.72	127.85	7.00E-07
F35C11.3 /// M05D6.1	F35C11.3 is an ortholog of human TTBK2 (tau tubulin kinase 2) and TTBK1 (tau tubulin kinase 1); F35C11.3 is predicted to have protein kinase activity and ATP binding activity, based on protein domain information. M05D6.1 is an ortholog of human TTBK2 (tau tubulin kinase 2) and TTBK1 (tau tubulin kinase 1); M05D6.1 is predicted to have protein kinase activity and ATP binding activity, based on protein domain information.	139.69	57.41	51.37	29.87	23.52	9.41E-05
F35D11.3	F35D11.3 is an ortholog of human TMCO4 (transmembrane and coiled-coil domains 4).	69.52	209.82	120.17	102.72	96.94	2.28E-04
F36A2.11	F36A2.11	51.05	26.31	22.29	17.14	17.14	1.61E-04
F36A2.14	F36A2.14	41.85	24.31	20.63	17.27	19.76	1.42E-04
F36A4.2	F36A4.2	153.50	75.04	48.84	26.49	20.64	6.24E-05
F36A4.4	F36A4.4	75.68	51.80	35.93	22.14	22.07	3.46E-05
F36F2.2	F36F2.2	24.68	320.68	294.77	246.91	193.16	2.09E-04

F36H12.8 /// R13H9.5	F36H12.8 /// R13H9.5	171.86	79.79	64.73	33.23	32.16	5.91E-05
F36H12.9 /// R13H9.6	F36H12.9 is an ortholog of human TTBK2 (tau tubulin kinase 2) and TTBK1 (tau tubulin kinase 1); F36H12.9 is predicted to have protein kinase activity and ATP binding activity, based on protein domain information. R13H9.6 is an ortholog of human TTBK2 (tau tubulin kinase 2) and TTBK1 (tau tubulin kinase 1); R13H9.6 is predicted to have protein kinase activity and ATP binding activity, based on protein domain information.	114.04	60.64	44.49	29.73	26.08	3.52E-05
F37A4.4	Ankyrin repeat-containing protein F37A4.4	50.76	22.87	23.18	17.47	19.73	7.29E-05
F37A4.5	F37A4.5 is an ortholog of human PSMD14 (proteasome (prosome, macropain) 26S subunit, non-ATPase, 14).	101.89	50.45	32.70	21.06	21.86	1.52E-04
F38H4.5	F38H4.5 is an ortholog of human DDAH2 (dimethylarginine dimethylaminohydrolase 2) and DDAH1 (dimethylarginine dimethylaminohydrolase 1); F38H4.5 is predicted to have hydrolase activity, acting on carbon-nitrogen (but not peptide) bonds, in linear amidines, based on protein domain information.	35.57	21.59	20.74	16.94	17.69	6.08E-05
F40H3.2	F40H3.2	108.09	41.61	41.85	40.84	49.35	2.04E-04
F40H6.1	F40H6.1	138.62	60.43	36.76	25.58	21.86	4.11E-05
F41E6.12	F41E6.12	249.32	52.75	117.80	202.13	171.01	6.00E-07
F41E6.5	F41E6.5 is an ortholog of human HAO2 (hydroxyacid oxidase 2 (long chain)) and HAO1 (hydroxyacid oxidase (glycolate oxidase) 1); F41E6.5 is predicted to have FMN binding activity and oxidoreductase activity, based on protein domain information.	56.53	461.48	44.45	31.99	43.33	3.00E-07
F41G4.8	F41G4.8	27.07	170.45	152.55	120.06	91.41	9.60E-06
F42A9.7	Major sperm protein	115.06	43.91	34.48	22.70	20.43	2.10E-06
F42C5.5	F42C5.5 is an ortholog of human PTPN22 (protein tyrosine phosphatase, non-receptor type 22 (lymphoid)), PTPN18 (protein tyrosine phosphatase, non-receptor type 18 (brain-derived)) and PTPN12 (protein tyrosine phosphatase, non-receptor type 12); F42C5.5 is predicted to have protein tyrosine phosphatase activity, based on protein domain information.	19.23	11.38	8.94	7.32	7.13	7.79E-05
F42C5.9	F42C5.9 is localized to the plasma membrane and the striated muscle dense body.	111.04	250.93	148.25	149.12	140.91	5.95E-05
F43C11.7	F43C11.7 is an ortholog of human TRIM38 (tripartite motif containing 38), TRIM11 (tripartite motif containing 11), TRIM50 (tripartite motif containing 50), TRIM74 (tripartite motif containing 74) and TRIM73 (tripartite motif containing 73); F43C11.7 is involved in defense response to Gram-positive bacterium; F43C11.7 is predicted to have zinc ion binding activity, based on protein domain information.	102.86	1588.20	462.96	154.01	121.82	7.15E-05
F43C9.1	This gene encodes a protein containing an F-box, a motif predicted to mediate protein-protein interactions either with homologs of yeast Skp-1p or with other proteins.	30.84	26.46	687.82	45.91	30.51	4.20E-06
F43G6.8	F43G6.8 is an ortholog of human RNF224 (ring finger protein 224) and RNF182 (ring finger protein 182); F43G6.8 is predicted to have zinc ion binding activity, based on protein domain information.	69.54	341.54	107.58	94.31	96.33	2.00E-06
F44D12.8	F44D12.8	45.25	23.41	19.47	16.06	16.90	1.70E-06
F44G4.5	F44G4.5	52.09	29.96	20.70	18.59	14.51	1.16E-05
F45D3.4	F45D3.4	191.30	2174.86	291.95	169.44	197.19	2.64E-05
F46C3.2	F46C3.2	13.81	38.51	37.64	37.29	29.10	1.12E-04
F46G10.2	F46G10.2	56.26	127.29	49.36	46.82	53.13	2.80E-06
F47B3.2 /// F47B3.7	F47B3.2 is an ortholog of human PTPN22 (protein tyrosine phosphatase, non-receptor type 22 (lymphoid)), PTPN18 (protein tyrosine phosphatase, non-receptor type 18 (brain-derived)) and PTPN12 (protein tyrosine phosphatase, non-receptor type 12); F47B3.2 is predicted to have protein tyrosine phosphatase activity, based on protein domain information. F47B3.7 is an ortholog of human PTPN22 (protein tyrosine phosphatase, non-receptor type 22 (lymphoid)), PTPN18 (protein tyrosine phosphatase, non-receptor type 18 (brain-derived)) and PTPN12 (protein tyrosine phosphatase, non-receptor type 12); F47B3.7 is predicted to have protein tyrosine phosphatase activity, based on protein domain information.	236.79	108.99	74.68	54.02	52.90	4.62E-05

F47B3.7	F47B3.7 is an ortholog of human PTPN22 (protein tyrosine phosphatase, non-receptor type 22 (lymphoid)), PTPN18 (protein tyrosine phosphatase, non-receptor type 18 (brain-derived)) and PTPN12 (protein tyrosine phosphatase, non-receptor type 12); F47B3.7 is predicted to have protein tyrosine phosphatase activity, based on protein domain information.	48.03	21.95	16.70	13.15	13.94	2.40E-04
F47B8.4	F47B8.4 is an ortholog of human GLRX5 (glutaredoxin 5); F47B8.4 is involved in innate immune response; F47B8.4 is predicted to have electron carrier activity and protein disulfide oxidoreductase activity, based on protein domain information.	8.61	58.43	9.57	8.93	10.09	1.39E-05
F47D12.6	F47D12.6	83.17	472.81	492.92	533.13	346.96	4.83E-05
F47D12.7	F47D12.7 is an ortholog of human IVNS1ABP (influenza virus NS1A binding protein), KLHL6 (kelch-like family member 6), KLHDC8A (kelch domain containing 8A) and KLHDC8B (kelch domain containing 8B).	54.55	27.83	23.80	15.97	14.97	2.27E-04
F48E3.8	F48E3.8 encodes a polysaccharide deacetylase.	14.79	42.96	28.47	31.04	27.65	1.15E-04
F49C12.14	F49C12.14	187.73	90.95	264.95	305.83	303.46	2.03E-05
F49C12.15	F49C12.15	112.45	73.87	55.01	39.11	36.01	7.55E-05
F52G2.3	F52G2.3	44.42	81.60	109.72	99.96	83.01	6.00E-06
F52H3.6 /// ZK938.1	F52H3.6 is an ortholog of human PPP1CC (protein phosphatase 1, catalytic subunit, gamma isozyme) and PPP1CA (protein phosphatase 1, catalytic subunit, alpha isozyme); F52H3.6 is predicted to have hydrolase activity, based on protein domain information.	119.84	64.80	35.16	17.26	14.43	1.73E-04
F53A9.6	F53A9.6 is involved in innate immune response.	214.70	888.24	901.85	700.04	562.36	2.33E-05
F53A9.8	F53A9.8 is involved in defense response to Gram-positive bacterium.	306.39	7321.45	6373.12	5117.89	3348.03	1.30E-06
F53B2.8	F53B2.8	42.61	428.91	72.49	82.82	99.39	3.09E-05
F53B6.4	Major sperm protein	632.00	268.59	203.44	112.05	79.02	1.83E-04
F53E10.1	F53E10.1 is an ortholog of human MARC1 (mitochondrial amidoxime reducing component 1) and MARC2 (mitochondrial amidoxime reducing component 2); F53E10.1 is predicted to have catalytic activity, molybdenum ion binding activity, and pyridoxal phosphate binding activity, based on protein domain information.	151.95	384.72	163.32	160.62	163.64	3.49E-05
F53F1.6	F53F1.6	18.99	74.06	140.41	89.93	52.99	2.54E-04
F53F10.1	F53F10.1 is an ortholog of human CCDC102A (coiled-coil domain containing 102A) and CCDC102B (coiled-coil domain containing 102B); F53F10.1 is localized to the mitochondrion.	51.90	150.22	83.17	77.39	72.10	2.24E-04
F53H8.3	F53H8.3 is an ortholog of human SLC2A14 (solute carrier family 2 (facilitated glucose transporter), member 14), SLC2A3 (solute carrier family 2 (facilitated glucose transporter), member 3), SLC2A1 (solute carrier family 2 (facilitated glucose transporter), member 1), SLC2A2 (solute carrier family 2 (facilitated glucose transporter), member 2) and SLC2A4 (solute carrier family 2 (facilitated glucose transporter), member 4); F53H8.3 is predicted to have substrate-specific transmembrane transporter activity, based on protein domain information.	93.56	87.10	216.49	222.55	190.92	4.12E-05
F54C1.8	F54C1.8	92.25	51.32	39.65	31.00	30.89	1.14E-05
F55B11.1	The F55B11.1 gene encodes an ortholog of the human gene XANTHINE DEHYDROGENASE (XDH), which when mutated leads to xanthinuria (OMIM:278300).	57.97	111.48	218.66	205.02	140.39	1.78E-04
F55F8.7	F55F8.7 is predicted to have protein tyrosine phosphatase activity, based on protein domain information.	45.44	35.64	30.12	26.67	30.11	6.71E-05
F55H12.5	F55H12.5 is predicted to have protein tyrosine phosphatase activity, based on protein domain information.	65.53	33.54	30.76	23.37	20.43	2.23E-04
F56B3.6	F56B3.6 is an ortholog of human REEP6 (receptor accessory protein 6) and REEP5 (receptor accessory protein 5).	180.23	93.93	65.24	44.89	34.85	6.23E-05
F56D6.13	F56D6.13	166.20	121.66	74.53	45.84	47.23	2.12E-05
F58A6.9	F58A6.9	2440.82	1439.56	990.83	560.16	394.82	2.35E-04
F58D5.2	F58D5.2	152.04	575.55	598.38	480.92	419.63	1.41E-04
F58D5.7	F58D5.7	45.95	23.55	20.89	15.73	14.70	3.10E-06
F58E6.5	F58E6.5 is an ortholog of human VAPB (VAMP (vesicle-associated membrane protein)-associated	141.64	59.32	47.52	28.83	29.86	6.51E-05

	protein B and C) and VAPA (VAMP (vesicle-associated membrane protein)-associated protein A).						
F58F9.4	F58F9.4	49.46	43.20	131.90	101.22	79.76	1.24E-05
F58G6.3 /// F58G6.7	F58G6.7 is an ortholog of human SLC31A2 (solute carrier family 31 (copper transporter), member 2) and SLC31A1 (solute carrier family 31 (copper transporter), member 1); F58G6.7 is predicted to have copper ion transmembrane transporter activity, based on protein domain information.	567.61	114.87	440.66	568.29	641.82	1.49E-04
F58H1.6	F58H1.6	93.09	43.11	34.20	32.30	34.87	6.77E-05
F59B2.13	F59B2.13 is an ortholog of human GPR142 (G protein-coupled receptor 142) and GPR139 (G protein-coupled receptor 139); F59B2.13 is predicted to have G-protein coupled receptor activity, based on protein domain information.	54.90	104.16	165.37	120.71	100.86	6.26E-05
F59E11.5	F59E11.5	107.38	44.37	44.26	39.11	41.45	1.25E-04
F59F5.3	F59F5.3 is an ortholog of human EPHA4 (EPH receptor A4), EPHB2 (EPH receptor B2), EPHA10 (EPH receptor A10), EPHB1 (EPH receptor B1) and EPHB3 (EPH receptor B3); F59F5.3 is predicted to have protein tyrosine kinase activity, based on protein domain information.	6.35	10.17	6.16	6.56	6.84	7.26E-05
fard-1	fard-1 is an ortholog of human FAR1 (fatty acyl CoA reductase 1) and FAR2 (fatty acyl CoA reductase 2); fard-1 is predicted to have fatty-acyl-CoA reductase (alcohol-forming) activity, based on protein domain information.	42.21	118.90	203.19	203.78	176.59	3.25E-05
fbp-1	fbp-1 encodes fructose 1,6-bisphosphatase, a gluconeogenic enzyme that catalyzes the hydrolysis of fructose 1,6-bisphosphate to fructose 6-phosphate and inorganic phosphate in a reaction that reverses the third enzymatic step of glycolysis; FBP-1 is orthologous to human FBP1 (OMIM:229700, mutated in FBP deficiency).	1081.04	642.55	960.32	995.14	1110.29	2.00E-06
fbxa-115	This gene encodes a protein containing an F-box, a motif predicted to mediate protein-protein interactions either with homologs of yeast Skp-1p or with other proteins; this gene's encoded protein also contains an FTH/DUF38 motif, which may also mediate protein-protein interaction.	23.91	42.54	48.08	61.25	55.28	1.69E-05
fbxa-156	This gene encodes a protein containing an F-box, a motif predicted to mediate protein-protein interactions either with homologs of yeast Skp-1p or with other proteins; this gene's encoded protein also contains an FTH/DUF38 motif, which may also mediate protein-protein interaction.	101.22	274.57	189.60	201.70	188.16	1.90E-04
fbxa-162	This gene encodes a protein containing an F-box, a motif predicted to mediate protein-protein interactions either with homologs of yeast Skp-1p or with other proteins; this gene's encoded protein also contains an FTH/DUF38 motif, which may also mediate protein-protein interaction.	14.76	51.64	16.17	19.40	21.54	1.79E-04
fbxa-196	This gene encodes a protein containing an F-box, a motif predicted to mediate protein-protein interactions either with homologs of yeast Skp-1p or with other proteins; this gene's encoded protein also contains an FTH/DUF38 motif, which may also mediate protein-protein interaction.	13.24	9.62	8.45	7.89	8.47	7.81E-05
fbxc-1-5	fbxc-1-5	142.55	274.06	308.07	336.97	356.20	8.52E-05
fis-1	fis-1 encodes a protein similar to the yeast and human Fis1 proteins involved in mitochondrial fission; fis-1 does not seem to be required for mitochondrial fission in C. elegans, but it is possible that it may function redundantly along with fis-2 and other genes; fis-1 expression is enriched in the germline.	29.68	18.06	15.50	12.71	14.08	5.24E-05
flp-10	flp-10 encodes a FMRFamide-related neuropeptide; in males, flp-10 activity is required for a sensory transduction pathway that negatively regulates the frequency of certain substeps of turning behavior during mating; a flp-10::gfp reporter is expressed in a number of neurons including AIM, ASI, AUA, BAG, BDU, DVB, PQR, PVR, and URX, and in the vulD cells.	86.53	256.27	131.81	109.74	106.28	1.74E-04
fmi-1	fmi-1 encodes a highly conserved cadherin-like protein with unique domain composition of six EGF and two laminin G domains, a GPS cleavage site and seven-pass transmembrane domain; fmi-1 plays a crucial role in pioneer axon navigation as well as pioneer-mediated follower navigation; FMI-1 is required cell autonomously in pioneer and partially non-cell autonomously in the follower; different domains of FMI-1 are required for pioneer and follower navigation; mutations in fmi-1 cause strong axon navigation defects of VNC pioneer axons; HSN axons are more susceptible to changes in fmi-1 protein; LIN-17/frizzled functions together with fmi-1 in follower axon navigation; in addition, FMI-1 functions together with the CDH-4 cadherin to regulate GABAergic neuronal development; FMI-1 is expressed in neurons and localized to pioneer and follower axons during and after nervous system development; in the embryos FMI-1::GFP is found in axons in the nerve	16.03	54.07	30.70	52.62	30.05	2.80E-05

	ring, the tail and along the dendrites of sensory neurons.						
fmo-1	fmo-1 encodes a flavin-containing monoxygenase homologous to human FMO1, FMO2, and FMO3 (OMIM:602079, mutated in trimethylaminuria).	51.40	447.70	40.30	34.78	36.86	6.00E-07
fmo-2	fmo-2 encodes a flavin-containing monoxygenase homologous to human FMO1, FMO2, and FMO3 (OMIM:602079, mutated in trimethylaminuria).	16.07	2693.12	84.98	53.75	52.61	6.70E-06
fmo-3	fmo-3 encodes a flavin-containing monoxygenase homologous to human FMO1, FMO2, and FMO3 (OMIM:602079, mutated in trimethylaminuria).	52.27	19.05	21.67	18.72	22.67	1.51E-04
frpr-11	frpr-11 is predicted to have G-protein coupled receptor activity, based on protein domain information.	20.37	106.92	20.76	22.94	22.57	1.04E-04
ftn-1	ftn-1 encodes one of two C. elegans ferritin heavy chain homologs; ftn-1 activity is essential for normal lifespan under iron stress conditions and, in addition, has been reported to be essential for embryogenesis; an ftn-1::gfp reporter is expressed in the intestine at all stages of development and its expression, as well as that of ftn-1 mRNA, increases under iron stress conditions and in the background of mutations in the second ferritin-encoding gene, ftn-2.	230.92	936.24	82.91	98.19	167.48	1.59E-04
gale-1	gale-1 encodes a putative UDP-galactose-4-epimerase, orthologous to human GALE (OMIM:606953, mutated in galactose epimerase deficiency); GALE-1 is thought to interconvert UDP-N-acetylglucosamine and UDP-N-acetylgalactosamine at the end of the hexosamine pathway; gale-1 is expressed in neurons, intestine and the developing reproductive system, but has no observed function in mass RNAi assays.	791.87	334.96	411.48	500.33	535.71	2.25E-04
gipc-1	RGS-GAIP Interacting Protein C; gipc-1 is an ortholog of human GIPC2 (GIPC PDZ domain containing 2), GIPC3 (GIPC PDZ domain containing 3) and GIPC1 (GIPC PDZ domain containing 1).	462.56	266.39	157.17	82.18	67.26	4.36E-05
gipc-2	RGS-GAIP interacting protein C; gipc-2 is an ortholog of human GIPC2 (GIPC PDZ domain containing 2), GIPC3 (GIPC PDZ domain containing 3) and GIPC1 (GIPC PDZ domain containing 1).	587.10	284.20	162.81	89.29	59.30	9.30E-05
gln-1	gln-1 is an ortholog of human GLUL (glutamate-ammonia ligase); gln-1 is predicted to have glutamate-ammonia ligase activity, based on protein domain information.	102.90	70.23	233.35	178.09	138.52	4.70E-06
glo-4	glo-4 encodes a guanine nucleotide exchange factor (GEF) that is similar to Drosophila Claret and human RP3; throughout development, glo-4 activity is required for biogenesis of the lysosome-related gut granules; based upon phenotypic similarity, glo-4 is likely to encode a GEF for the GLO-1 Rab-like GTPase; in regulating gut granule formation, glo-4 acts downstream of apb-3, which encodes the AP-3 complex beta subunit.	87.69	151.71	82.77	74.17	79.87	1.30E-05
glt-5	glt-5 encodes an ortholog of glutamate/aspartate and neutral amino acid transporters.	15.41	162.73	64.42	34.05	28.21	2.01E-05
gly-8	gly-8 encodes a predicted transmembrane polypeptide N-acetylgalactosaminyl transferase (ppGaNTase) similar to the essential Drosophila ppGaNTase encoded by Pgant35A; by homology, GLY-8 is predicted to function as a Golgi enzyme that catalyzes transfer of N-acetylgalactosamine to serine and threonine-containing acceptor peptides, thus initiating mucin-type O-glycosylation; as loss of gly-8 activity via large-scale RNAi screens does not result in any obvious abnormalities, the precise role of GLY-8 in C. elegans development and/or behavior is not yet known.	179.31	75.08	114.11	114.65	108.79	1.14E-04
gpa-9	gpa-9 encodes a member of the G protein alpha subunit family of heterotrimeric GTPases; it is expressed in ASJ, PHB, PVQ, pharyngeal muscle, and the spermatheca.	25.57	83.31	95.65	76.15	71.34	4.03E-05
gpdh-1	Glycerol-3-Phosphate DeHydrogenase	83.46	37.02	334.18	270.11	154.39	1.00E-07
gska-3	gska-3 is an ortholog of human MAPK15 (mitogen-activated protein kinase 15); gska-3 is predicted to have protein kinase activity and ATP binding activity, based on protein domain information.	119.88	60.07	48.34	37.21	28.92	2.94E-05
gsp-3	gsp-3 encodes an ortholog of protein phosphatase 1 gamma, a Glc7/PP1 phosphatase (HGNC:PPP1CA); GSP-3 is nearly identical to GSP-4, and the two proteins appear to function redundantly during sperm development; GSP-3/4 activity is required for chromosome partitioning during sperm meiosis and for sperm motility, specifically for pseudopod development and a normal rate of pseudopodial treadmilling; in regulating sperm development, GSP-3/4 functions upstream of, and regulates the localization of, the major sperm proteins (MSPs); GSP-3/4 may also play a role in egg laying; GSP-3/4 is expressed at high levels in sperm, with strong localization seen around meiotic and mature sperm DNA.	518.17	208.28	149.95	71.36	60.64	2.49E-04
gst-22	gst-22 is an ortholog of human HPGDS (hematopoietic prostaglandin D synthase).	110.54	812.84	385.81	326.73	350.93	2.03E-05

gst-26	gst-26 is an ortholog of human HPGDS (hematopoietic prostaglandin D synthase).	702.82	120.29	534.34	806.51	880.65	7.40E-06
gsto-3	K10F12.4 encodes, by alternative splicing, one isoform of a putative omega-class glutathione transferase (GST; EC 2.5.1.18) which, like its paralog GSTO-1, might have thiol oxidoreductase and dehydroascorbate reductase activity; other K10F12.4 paralogs include GST-44 and C02D5.3; K10F12.4 has no obvious function in mass RNAi assays.	110.27	342.01	179.88	131.18	116.45	6.51E-05
H06A10.1	H06A10.1	68.30	16.73	25.35	19.85	23.29	7.13E-05
H06I04.6	H06I04.6	111.80	219.66	122.01	112.89	126.26	1.85E-05
H19N07.3	H19N07.3	454.28	1167.16	611.57	505.86	500.73	1.91E-04
H20E11.3	H20E11.3 is involved in innate immune response.	54.84	107.85	193.76	142.11	104.88	1.49E-04
H32C10.1	H32C10.1 is an ortholog of human CECR5 (cat eye syndrome chromosome region, candidate 5).	67.56	35.37	32.63	26.60	21.67	2.46E-04
haao-1	haao-1 is an ortholog of human HAAO (3-hydroxyanthranilate 3,4-dioxygenase); haao-1 is predicted to have 3-hydroxyanthranilate 3,4-dioxygenase activity and iron ion binding activity, based on protein domain information.	186.22	35.43	150.05	184.98	139.08	5.60E-06
hex-1	hex-1 encodes a beta-N-acetylhexosaminidase that is orthologous to the human gene CERVICAL CANCER PROTO-ONCOGENE 7 (HEXB; OMIM:606873), which when mutated leads to disease.	74.12	531.22	561.20	433.94	423.49	7.90E-06
hgo-1	hgo-1 encodes a putative homogentisate 1,2-dioxygenase, orthologous to human HGD (OMIM:607474, mutated in alkaptonuria), that is required for normal resistance to hypertonic stress; HGO-1 is expressed in larval and adult hypodermis and intestine, and in larval pharynx; HGO-1 is required for the tyrosinemic phenotype of K10C2.4(RNAi) animals.	593.34	355.54	860.05	1123.02	1152.63	8.56E-05
hil-1	hil-1 is an ortholog of human H1F0 (H1 histone 0); hil-1 is predicted to have DNA binding activity, based on protein domain information; hil-1 is localized to the nucleolus and the intermediate filament cytoskeleton.	62.90	310.82	85.03	69.33	72.80	5.20E-06
his-41	his-41 encodes an H2B histone.	461.20	498.70	783.43	869.28	783.74	2.57E-04
hmgs-1	F25B4.6 is orthologous to the human gene 3-HYDROXY-3-METHYLGLUTARYL COA SYNTHASE (HMGCS2; OMIM:600234), which when mutated leads to disease.	212.36	260.48	359.80	360.82	324.68	1.80E-04
hpd-1	hpd-1 encodes a putative 4-hydroxyphenylpyruvate dioxygenase required for normally short lifespan and for negative regulation of the dauer larval stage, with loss of hpd-1 function prolonging lifespan and promoting dauer formation; hpd-1 is a (perhaps evolutionarily conserved) target of transcriptional activation by DAF-16; HPD-1 is orthologous to human HPD (OMIM:609695, mutated in tyrosinemia type III), and is paralogous to C31H2.4 and human HPDL/GLOXD1; HPD-1 is expressed in hypodermis and intestine, and is required for the tyrosinemic phenotype of K10C2.4(RNAi) animals.	745.39	397.44	2030.51	2095.98	2074.57	8.80E-06
hprt-1	hprt-1 encodes an ortholog of human HYPOXANTHINE PHOSPHORIBOSYLTRANSFERASE 1 (HPRT1), which when mutated leads to Lesch-Nyhan syndrome (OMIM:308000).	942.95	583.75	1558.39	1969.78	1487.69	3.53E-05
hrg-1	hrg-1 encodes a transmembrane protein that is orthologous to vertebrate heme transporters; in C. elegans, hrg-1 functions to mediate heme homeostasis, likely by regulating intracellular heme availability from endosomal or lysosomal-like compartments; HRG-1 exhibits pH-dependent heme binding in vitro and when expressed in Xenopus oocytes, can stimulate heme-dependent currents; an hrg-1::gfp promoter fusion is expressed in the larval and adult intestine in response to heme deficiency; when expressed in HEK293 cells, HRG-1 localizes primarily to endosomes and lysosome-related organelles; HRG-1 function is likely conserved across species, as hrg-1 expression is able to rescue developmental defects seen in zebrafish hrg-1 mutants.	572.71	1708.24	953.36	1324.54	1411.37	1.21E-05
hrg-4	hrg-4 encodes a transmembrane protein that is conserved amongst nematodes; hrg-4 functions to mediate heme homeostasis, likely by regulating heme transport across the plasma membrane; in vitro, HRG-4 exhibits heme binding over a broad pH range and when expressed in Xenopus oocytes, can stimulate heme-dependent currents; when expressed in HEK293 cells, HRG-4 localizes primarily to the plasma membrane; hrg-4 mRNA expression is significantly upregulated in response to heme deficiency.	219.84	60.49	512.10	619.28	513.57	3.40E-06
hsp-16.1 /// hsp-16.11	hsp-16.1 /// hsp-16.11	83.98	46.94	1729.94	1181.90	544.82	2.07E-05

hsp-16.2	hsp-16.2 encodes a 16-kD heat shock protein (HSP) that is a member of the hsp16/hsp20/alphaB-crystallin (HSP16) family of heat shock proteins; hsp-16.2 expression, strongest in intestine and pharynx, is induced in response to heat shock or other environmental stresses; HSP-16.2 has been shown to interact with intracellular human beta amyloid peptide, a primary component of the extracellular plaques found in Alzheimer's disease; HSP-16.2 is likely to function as a passive ligand temporarily preventing unfolded proteins from aggregating.	19.32	20.14	827.08	798.26	363.41	7.20E-06
hsp-16.41	hsp-16.41 encodes a 16-kD heat shock protein (HSP) that is a member of the hsp16/hsp20/alphaB-crystallin (HSP16) family of heat shock proteins; an hsp-16.41 reporter fusion, expressed broadly but strongest in intestine and pharynx, is induced in response to heat shock or other environmental stresses; expression is detectable in somatic tissues in post-gastrulation embryos, all larval stages, and in adults; HSP-16.41 is likely to function as a passive ligand temporarily preventing unfolded proteins from aggregating.	12.50	10.29	812.22	1122.89	517.81	3.00E-07
hsp-16.48 /// hsp-16.49	hsp-16.48 /// hsp-16.49	129.06	49.86	1625.87	1737.43	704.79	5.90E-05
hsp-17	hsp-17 encodes a heat shock protein that is a member of the hsp16/hsp20/alpha-crystallin family of heat shock proteins; by homology, HSP-17 is predicted to function as a molecular chaperone that protects cells from heat-induced protein aggregation and denaturation.	281.41	108.31	1554.78	2075.84	1558.80	7.44E-05
htas-1	htas-1 is an ortholog of human H2AFX (H2A histone X), H2AFJ (H2A histone J), HIST2H2AC (histone cluster 2, H2ac), HIST2H2AA4 (histone cluster 2, H2aa4) and HIST1H2AJ (histone cluster 1, H2aj); htas-1 is predicted to have DNA binding activity and protein heterodimerization activity, based on protein domain information.	329.25	195.92	125.71	67.92	53.95	8.50E-05
icl-1	icl-1 encodes a predicted isocitrate lyase/malate synthase, an enzyme known to function in the glyoxylate cycle; ICL-1 was identified in a screen for proteins that interact with GEX-3, a homolog of human HEM-2 which is required for embryonic development; ICL-1 is required for embryonic morphogenesis and appears to act downstream of DAF-16 to influence lifespan.	254.59	4054.13	1075.02	378.90	279.72	5.31E-05
ikb-1	ikb-1 encodes an ortholog of human BCL3 (OMIM:109560) that physically interacts with the checkpoint protein MRT-2; ikb-1(nr2019) and ikb-1(nr2027) mutants display no obvious phenotype other than shortened lifespan; mutation of BCL3 is frequently associated with recurring translocations found in the neoplastic cells of patients with chronic lymphocytic leukemia, and has been implicated in cell cycle control (perhaps via apoptosis).	26.83	165.50	39.24	44.55	35.10	4.00E-07
irld-14	Insulin/EGF-Receptor L Domain protein	32.11	19.31	18.43	14.45	17.02	7.98E-05
ist-1	ist-1 encodes a pleckstrin homology (PH) and phosphotyrosine binding (PTB) domain-containing insulin receptor substrate (IRS) homolog that negatively regulates lifespan and dauer development; IST-1 potentiates insulin-like signaling, although it is not absolutely required for such signaling under most conditions; in addition to acting through the AGE-1/PI3K branch of the insulin-like signaling pathway, IST-1 may also function in a parallel pathway to activate downstream protein-kinase Bs encoded by akt-1 and akt-2.	13.77	60.13	13.68	12.15	13.23	6.70E-06
K01A2.10	K01A2.10 is an ortholog of human CCDC92 (coiled-coil domain containing 92).	38.91	162.44	44.75	43.98	45.91	2.20E-06
K01H12.4	K01H12.4	154.21	74.63	58.57	40.26	37.69	2.50E-04
K02A6.3	This gene encodes a protein containing an F-box, a motif predicted to mediate protein-protein interactions either with homologs of yeast Skp-1p or with other proteins.	74.18	152.88	57.08	64.01	82.19	3.31E-05
K03A11.5	K03A11.5	26.88	62.04	27.33	33.81	30.46	5.59E-05
K03E6.7	K03E6.7 is an ortholog of human SH3BP5L (SH3-binding domain protein 5-like).	22.94	60.73	22.42	26.76	28.53	1.75E-05
K03H1.5	K03H1.5 is an ortholog of human SUSD2 (sushi domain containing 2).	50.03	124.10	41.94	52.63	59.81	1.45E-04
K04F10.1	The K04F10.1 gene encodes a homolog of SCA1, which when mutated leads to spinocerebellar ataxia 1 (OMIM:164400).	33.26	51.35	63.03	68.88	67.10	1.07E-04
K04G2.4	K04G2.4	297.90	141.04	94.12	53.10	38.97	1.84E-04
K05C4.2	K05C4.2 is an ortholog of human JAGN1 (jagunal 1 (Drosophila)).	1729.55	1330.13	1702.64	1929.07	1940.79	1.89E-04

K05F1.10	K05F1.10 encodes a putative secreted TIL-domain protease inhibitor paralogous to SWM-1, ISL-1, and the products of 11 other C. elegans genes; K05F1.10 and its relatives are collectively similar to other TIL-domain protease inhibitors from nematodes, insects, and vertebrates; K05F1.10 transcription is reproducibly induced by infection, suggesting that it may participate in innate immunity.	121.27	62.15	112.80	226.92	267.42	1.22E-04
K05F1.9	Major sperm protein	214.96	117.94	77.39	43.69	41.42	1.05E-04
K06A1.2	K06A1.2	20.48	13.77	13.73	13.02	13.55	1.46E-04
K06A5.2	K06A5.2	448.62	230.36	154.40	72.29	49.34	7.12E-05
K06A5.3	Major sperm protein	53.36	34.65	26.44	15.37	14.48	2.30E-04
K07A1.3	K07A1.3 is an ortholog of human C19orf54 (chromosome 19 open reading frame 54).	68.92	126.26	103.72	110.44	103.55	1.94E-04
K07A1.4	K07A1.4	75.44	39.86	27.99	20.02	18.56	6.47E-05
K07A1.6/// R10H1.1	K07A1.6 encodes a putative secreted TIL-domain protease inhibitor paralogous to SWM-1, ISL-1, and the products of 11 other C. elegans genes; K07A1.6 and its relatives are collectively similar to other TIL-domain protease inhibitors from nematodes, insects, and vertebrates; K07A1.6 has no obvious function in mass RNAi assays. R10H1.1 is an ortholog of human CCDC50 (coiled-coil domain containing 50	120.04	569.63	393.76	324.95	329.49	2.62E-05
K07C11.7	K07C11.7	309.86	1082.00	1207.07	1235.80	1171.70	5.93E-05
K08B12.3	K08B12.3 is an ortholog of human HDHD2 (haloacid dehalogenase-like hydrolase domain containing 2).	72.98	96.16	113.90	113.07	112.60	1.92E-04
K08C9.2	K08C9.2	267.81	127.80	87.87	47.61	47.37	2.60E-05
K08F4.5	K08F4.5	48.66	24.36	16.82	11.46	11.91	1.31E-04
K09C4.5	K09C4.5 is predicted to have transmembrane transporter activity, based on protein domain information.	51.40	15.37	17.64	21.64	24.35	1.65E-05
K10D11.2	K10D11.2 is an ortholog of human EPHX1 (epoxide hydrolase 1, microsomal (xenobiotic))	24.44	575.45	421.61	244.91	149.10	1.16E-05
K11C4.1	K11C4.1 is an ortholog of human TTBK2 (tau tubulin kinase 2) and TTBK1 (tau tubulin kinase 1); K11C4.1 is predicted to have protein kinase activity and ATP binding activity, based on protein domain information.	37.56	14.47	12.56	10.58	10.91	2.41E-04
K11C4.2	K11C4.2 is an ortholog of human PAQR7 (progestin and adipoQ receptor family member VII) and PAQR8 (progestin and adipoQ receptor family member VIII).	29.26	23.88	32.92	35.30	29.45	1.60E-04
K11E4.2	K11E4.2	66.85	181.70	137.12	157.87	153.44	2.31E-04
K11H3.3	K11H3.3 is an ortholog of human SLC25A1 (solute carrier family 25 (mitochondrial carrier; citrate transporter), member 1).	120.44	112.48	169.89	204.45	184.68	1.73E-05
kel-8	kel-8 is an ortholog of human KLHL8 (kelch-like family member 8); kel-8 is localized to the postsynaptic density, the neuron projection and the neuronal cell body.	148.64	206.67	262.17	271.03	282.10	3.53E-05
kgb-2	kgb-2 is an ortholog of human MAPK8 (mitogen-activated protein kinase 8); kgb-2 is predicted to have MAP kinase activity and ATP binding activity, based on protein domain information.	9.84	35.74	14.80	35.53	41.77	1.83E-04
kin-15	kin-15 encodes a novel receptor protein tyrosine kinase; like kin-16, with which it is co-transcribed, kin-15 encodes a protein that displays some unusual features, including a very small extracellular domain (50 amino acids) that lacks a cysteine-rich region typical of ligand-binding domains of known receptor tyrosine kinases, an unusual amino acid substitution in the subdomain VI motif, and a lack of typical autophosphorylation sites in the kinase insert and C-terminal domains; a kin-15 reporter is first detected in young L1 larvae in the hyp7 syncytium; later expression is detected in ventral and lateral cells that fuse with hyp7 as well as in the more anterior hyp6 syncytium late in development; this distinct expression pattern suggests that kin-15 may also play a role in development of the hypodermal syncytium and perhaps be involved in cell-cell interactions regulating postembryonic cell fusions.	72.89	17.90	18.70	23.97	27.78	1.11E-04
kin-21	kin-21 is predicted to have protein tyrosine kinase activity, based on protein domain information.	34.07	21.97	21.89	21.82	21.32	8.79E-05
kpc-1	kpc-1	36.23	135.14	51.98	52.36	49.94	1.00E-07
laat-1	laat-1 (lysosome associated amino acid transporter) is an ortholog of human PQLC2 (PQ loop	81.83	40.75	118.12	148.20	118.79	1.20E-06

	repeat containing 2).						
lact-2	lact-2 encodes a beta-lactamase domain-containing protein that contains a predicted transmembrane domain in its N terminus.	114.61	797.46	411.55	396.48	374.03	4.23E-05
lec-7	lec-7 is an ortholog of human LGALS3 (lectin, galactoside-binding, soluble, 3); lec-7 is predicted to have carbohydrate binding activity, based on protein domain information.	7.89	13.93	11.63	9.83	10.83	1.54E-04
lgg-2	lgg-2 encodes an ortholog of the autophagic budding yeast protein Atg8p; LGG-2 is also homologous to the light chain 3 (LC3) subunit of the microtubule-associated proteins 1A and 1B (MAP1A and MAP1B); LGG-2 is predicted to associate with MAP1A and MAP1B and with microtubules and to perhaps play a role in regulating the microtubule-binding activity of MAP1A and MAP1B; LGG-2 has no obvious function in RNAi assays, and is not required for either dauer formation or extended lifespan.	664.27	2006.99	580.69	624.70	643.86	3.00E-07
lim-9	lim-9 encodes a protein containing one PET domain and six LIM domains and is orthologous to Drosophila LIMPET and vertebrate FHL2; in vitro binding and yeast two-hybrid assays indicate that LIM-9 physically interacts with UNC-96 and UNC-97 and thus, is likely a component of a protein complex that links muscle focal adhesions to thick filaments; LIM-9 also enables Wnt-directed planar cell polarity and is required for the fully asymmetrical division of B.a versus B.p cells, though this requirement is quantitatively weak; a lim-9::gfp promoter fusion is expressed in pharyngeal and body wall muscles, as well as in some neuronal processes, vulva, spermathecae, anal sphincter and depressor muscles, gonadal sheath, and the excretory canal; staining with LIM-9 antibodies reveals that in body wall muscle LIM-9 localizes, at least partially, to M-lines, around which myosin thick filaments are organized.	52.37	75.00	113.80	83.66	81.58	7.75E-05
M01A8.1	M01A8.1	57.21	151.48	56.11	60.21	64.24	2.12E-05
M01B2.10	M01B2.10 is an ortholog of human PHYHIP (phytanoyl-CoA 2-hydroxylase interacting protein) and PHYHIPL (phytanoyl-CoA 2-hydroxylase interacting protein-like).	44.18	80.14	40.16	41.13	45.29	1.57E-05
M01H9.4	M01H9.4	57.64	147.25	94.01	97.74	93.39	1.90E-04
M163.1	M163.1	145.08	1186.46	278.50	398.44	356.90	4.36E-05
M57.1	M57.1	9.42	11.79	10.67	10.58	9.63	1.63E-04
M60.4	M60.4 is an ortholog of human MAL (mal, T-cell differentiation protein), MALL (mal, T-cell differentiation protein-like), CMTM8 (CKLF-like MARVEL transmembrane domain containing 8), MARVELD1 (MARVEL domain containing 1) and PLLP (plasmolipin).	465.88	2578.69	1996.51	1318.56	1007.59	1.57E-04
M70.2	M70.2	12.00	9.61	11.32	10.29	11.41	1.88E-04
maa-1	maa-1 encodes a novel, transmembrane acyl-CoA-binding protein; maa-1 activity, together with that of rme-1, is required for normal rates of endosomal recycling and for proper endosomal shape and morphology; a rescuing MAA-1::GFP fusion protein is expressed in the hypodermis (excluding the seam cells) and the intestine, as well as in an area near the dorsal and ventral nerve cords; within the hypodermis and intestine, MAA-1::GFP localizes to endosomal and Golgi vesicle membranes.	109.56	226.74	187.61	190.87	186.87	1.87E-04
mab-31	mab-31 encodes a novel protein that is highly conserved amongst nematodes; mab-31 functions in the TGF-beta signaling pathway to regulate the male tail ray patterning process, specifically the positioning of the R7-derived ray cell cluster; a mab-31::gfp reporter is expressed in a wide variety of tissues, including the intestine, hypodermis, pharynx, and neuronal support cells; in males, mab-31::gfp is also seen in the ray structural cells; a MAB-31::GFP localizes to the nucleus.	133.91	470.67	159.71	313.22	331.46	1.09E-05
madf-10	MADF domain transcription factor	50.20	139.58	67.33	58.94	62.24	1.78E-05
marc-2	marc-2 is an ortholog of human MARCH8 (membrane-associated ring finger (C3HC4) 8, E3 ubiquitin protein ligase), MARCH1 (membrane-associated ring finger (C3HC4) 1, E3 ubiquitin protein ligase) and MARCH2 (membrane-associated ring finger (C3HC4) 2, E3 ubiquitin protein ligase); marc-2 is predicted to have zinc ion binding activity, based on protein domain information.	29.57	18.61	15.82	13.43	13.51	1.09E-04
math-20	The C40D2.2 gene encodes a protein closely similar to C46F9.3, which has a meprin-associated Traf homology (MATH) domain and may be involved in apoptosis.	44.29	98.74	226.33	162.00	132.51	4.21E-05
math-32	MATH (meprin-associated Traf homology) domain containing	28.61	17.19	16.24	13.96	13.53	1.81E-04

mboa-3	mboa-3 encodes a putative transmembrane O-acyl transferase (MBOAT) orthologous to human MBOAT1 (OMIM:611732), and paralogous to C08F8.4 and ZK550.1; MBOA-3 has no obvious function in mass RNAi assays, and mboa-3(RNAi) animals incorporate exogenous arachidonic acid into phosphatidylcholine (PC), phosphatidylserine (PS) and phosphatidylethanolamine (PE) normally.	416.73	430.63	808.65	1132.48	1080.41	3.66E-05
memo-1	memo-1 is an ortholog of human MEMO1 (mediator of cell motility 1).	64.18	266.39	100.73	83.53	73.90	6.60E-06
mfb-1	This gene encodes a protein containing an F-box, a motif predicted to mediate protein-protein interactions either with homologs of yeast Skp-1p or with other proteins.	42.49	145.21	56.29	60.43	52.89	3.00E-07
mpk-2	mpk-2 encodes a mitogen activated protein (MAP) kinase; by homology, MPK-2 is predicted to function as a serine/threonine kinase that acts downstream of a receptor tyrosine kinase in a cell signaling pathway; however, as loss of mpk-2 activity via large-scale RNAi screens does not produce any obvious abnormalities, the precise role of MPK-2 in C. elegans development and/or behavior is not yet known.	22.58	67.08	31.11	27.53	22.29	3.50E-06
mpst-4	mpst-4 is an ortholog of human TST (thiosulfate sulfurtransferase (rhodanese)) and MPST (mercaptopyruvate sulfurtransferase).	45.79	16.35	16.60	14.88	15.80	1.22E-04
msd-2	msd-2 is localized to the pseudopodium and the cell body.	2980.64	1956.32	1373.17	651.48	500.27	1.95E-04
msp-45	msp-45 encodes a member of the major sperm protein family.	5829.22	3623.55	2740.64	1322.30	968.72	2.05E-04
msp-49	msp-49 encodes a protein that belongs to a family of proteins called the Major Sperm Proteins (MSPs) that is conserved in nematodes; this family consists of closely related, small, basic proteins that make up 15% of sperm protein; this multigene family consists of over fifty genes, including many pseudogenes; MSPs are involved in both extracellular signaling and cytoskeletal functions during reproduction-MSP antagonizes Eph/ephrin signaling, in part, by binding VAB-1 Eph receptor tyrosine kinase on oocytes and sheath cells to promote oocyte maturation and MAPK activation; MSPs assemble into fibrous networks that drive movement of the C. elegans sperm; msp genes are expressed only in late primary spermatocytes.	5554.63	3442.60	2526.65	1304.82	969.04	2.25E-04
nape-1	nape-1 is an ortholog of human NAPEPLD (N-acyl phosphatidylethanolamine phospholipase D); nape-1 is predicted to have zinc ion binding activity and N-acylphosphatidylethanolamine-specific phospholipase D activity, based on protein domain information.	33.50	78.80	45.24	33.73	37.41	1.08E-04
ncr-1	ncr-1 encodes a large transmembrane glycoprotein with a patched-like domain that is orthologous to human NPC1; NCR-1 and NPC1 are eukaryotic members of the resistance-nodulation-division (RND) family of membrane permeases, and have a putative sterol-sensing domain; by homology, NCR-1 is predicted to function in intracellular cholesterol and glycolipid trafficking; in C. elegans, NCR-1 is required for growth and survival in the absence of cholesterol, newly hatched ncr-1 mutant larvae grow poorly on cholesterol-free medium and die at the L1 or L2 stage; NCR-1 is also involved in negative regulation of dauer formation, being required redundantly with NCR-2, a second C. elegans NPC1-like protein, for preventing constitutive dauer formation; dauer formation in ncr-1; ncr-2 double mutants is suppressed by mutations in daf-12, which encodes a steroid hormone receptor, and by overexpression of daf-9, which encodes a cytochrome P450, suggesting that NCR-1 and NCR-2 may function to transport a sterol precursor that is metabolized by DAF-9 to then serve as the DAF-12 ligand.	58.21	128.36	104.10	74.62	67.05	5.59E-05
ncx-1	ncx-1 encodes, by alternative splicing, two isoforms of a putative 3Na[+]/1Ca[2+] exchanger, orthologous to human SLC8A1-3 and paralogous to NCX-2/-3; NCX-1 is predicted to export free cytoplasmic Ca[2+] with low affinity but high capacity, being complemented by low-capacity/high-affinity Ca[2+] ATPase pumps such as MCA-1/-3; NCX-3 has tandem Calx-alpha and Calx-beta domains predicted to carry out ion transport and regulation; NCX-1 is required for embryonic viability in mass RNAi assays.	101.79	51.74	49.30	43.11	37.77	1.23E-04
nfm-1	nfm-1 encodes a homolog of human merlin/schwannomin (NF2), which when mutated leads to neurofibromatosis, type 2 (OMIM:101000); at the same time, it is also homologous to the ERM family of cytoskeletal linkers with approximately equal similarity to ezrin, radixin and moesin.	59.53	223.73	169.88	128.71	134.07	2.90E-06
nhl-3	nhl-3 is predicted to have zinc ion binding activity, based on protein domain information.	27.39	37.81	23.34	23.31	24.59	2.21E-04
nhr-101	nhr-101	14.62	74.82	17.64	14.79	14.42	2.20E-06

nhr-109	nhr-109	67.03	174.60	86.04	93.70	106.11	1.15E-04
nhr-110	nhr-110	25.66	88.77	26.61	29.19	29.54	2.40E-06
nhr-117	nhr-117	10.76	28.23	10.49	10.66	12.49	7.30E-06
nhr-120	nhr-120	17.58	49.96	16.12	17.81	18.97	1.04E-04
nhr-122	nhr-122	58.79	246.76	62.99	75.76	66.64	1.00E-07
nhr-133	nhr-133	78.01	256.89	70.02	82.71	92.62	5.70E-06
nhr-143	nhr-143	9.26	29.80	9.55	10.06	12.50	8.38E-05
nhr-144	nhr-144	35.76	79.35	36.40	39.59	50.30	1.16E-04
nhr-162	nhr-162	10.68	29.76	11.81	11.08	10.66	1.14E-04
nhr-163	nhr-163	11.33	27.07	10.14	13.32	14.21	1.54E-04
nhr-170	nhr-170	9.67	34.78	15.00	14.52	14.10	1.00E-07
nhr-18	nhr-18	22.53	123.95	41.84	36.01	38.58	3.70E-06
nhr-206	nhr-206	11.86	46.21	13.36	13.87	13.64	8.00E-07
nhr-21	nhr-21	35.94	111.61	31.43	31.96	34.52	1.13E-04
nhr-211	nhr-211	17.05	34.00	16.87	20.49	21.20	5.37E-05
nhr-212	nhr-212	27.77	65.15	38.71	34.12	37.47	1.97E-05
nhr-232	nhr-232	19.74	76.80	17.86	39.00	33.77	3.70E-06
nhr-32	nhr-32	72.40	227.56	56.84	73.21	89.73	5.00E-07
nhr-36	nhr-36	11.81	31.37	11.10	12.28	13.44	5.47E-05
nhr-40	nhr-40	43.34	57.35	55.42	62.84	57.80	1.23E-05
nhr-50	nhr-50	26.00	59.28	43.66	35.61	30.51	1.40E-04
nhr-59	nhr-59	16.73	38.44	19.42	17.79	19.19	2.08E-05
nhr-64	nhr-64	69.83	211.51	77.82	91.60	87.22	2.32E-05
nhr-79	nhr-79	57.12	114.46	51.45	68.63	72.97	8.35E-05
nhr-8	nhr-8	80.71	315.24	217.79	272.15	207.40	1.36E-05
nhr-90	nhr-90	30.69	147.61	51.49	46.64	50.89	1.30E-06
nhr-99	nhr-99	19.60	53.32	18.74	18.87	17.40	2.58E-04
nlp-39	Neuropeptide-Like Protein	10.24	177.17	163.83	161.63	167.00	9.24E-05
npr-28	npr-28 is predicted to have G-protein coupled receptor activity, based on protein domain information.	45.32	136.23	66.83	67.75	77.17	2.55E-04
nspb-3 /// nspb-2	nspb-3 /// nspb-2	99.59	31.13	33.65	29.34	30.85	4.40E-06
nspe-3	Nematode Specific Peptide family, group E	52.24	38.92	115.26	84.30	64.12	2.29E-04
oat-1	oat-1 encodes a transmembrane organic anion transporter; although loss of oat-1 activity via large-scale RNAi screens results in no obvious abnormalities, when expressed in mammalian cells OAT-1 can transport a variety of structurally diverse organic anions via an anion exchange mechanism that is functionally coupled to a sodium-coupled dicarboxylate transporter; by homology with mammalian OAT1 transporters, C. elegans OAT-1 is predicted to function in xenobiotic elimination.	121.15	65.63	193.19	152.13	121.20	2.33E-04
pcp-2	pcp-2 encodes a prolyl carboxypeptidase.	123.62	26.69	114.92	123.39	111.61	3.30E-06
pcyt-1	pcyt-1 encodes a lipid-activated CTP:phosphocholine cytidylyltransferase (CCT), with CCT activity in vitro; recombinant PCYT-1 is most activated by a 1:1 mixture of phosphatidylcholine:oleate vesicles; an inhibitory 21-residue segment (residues 246-266) is critical for specific activation of PCYT-1 by lipids, since deletion or mutation of this segment causes PCYT-1 to be constitutively active without its normal lipid substrate.	612.34	238.45	928.91	1305.60	1349.04	1.00E-07
perm-2	PERMeable eggshell	4463.45	7071.90	6506.17	6337.66	6770.51	1.30E-04

pes-8	pes-8 encodes a novel protein that contains a proline-rich region and a predicted transmembrane domain, but no otherwise recognizably conserved domains; pes-8 was identified in promoter trapping screens and based on large-scale RNA-mediated interference (RNAi) screens, appears to be required for egg laying, germline development, locomotion, and regulation of adult lifespan; a pes-8 reporter is expressed at the cell surfaces of the spermathecal valve cell, the uterine wall, the vulva, and the rectal epithelium; strong expression is also detected in the uterus during its development; PES-8 may be required for normal morphology of the openings of the spermathecal valve, the vulva, and the rectum, a hypothesis consistent with its expression pattern and RNAi phenotype.	99.02	153.99	327.05	247.80	232.20	5.19E-05
pes-9	pes-9 encodes a zinc metallopeptidase; pes-9 was originally identified in a promoter trap screen for sequences that direct reporter gene expression in specific cell types during C. elegans development; pes-9 sequences direct expression in a limited number of cells in the early embryo and in putative head and tail hypodermal cells in adults; after some confusion, the pes-9 reporter gene fusion was mapped to R11H6.1.	1445.41	888.79	1811.44	2193.71	2256.94	7.40E-05
pho-13	pho-13 is an ortholog of human ACPT (acid phosphatase, testicular), ACPP (acid phosphatase, prostate) and ACP2 (acid phosphatase 2, lysosomal); pho-13 is predicted to have acid phosphatase activity, based on protein domain information.	76.73	18.43	90.74	123.00	130.98	8.30E-06
pitr-3	pitr-3 is an ortholog of human SLC20A1 (solute carrier family 20 (phosphate transporter), member 1) and SLC20A2 (solute carrier family 20 (phosphate transporter), member 2); pitr-3 is predicted to have inorganic phosphate transmembrane transporter activity, based on protein domain information.	35.33	120.95	111.49	93.19	87.16	7.34E-05
plc-2	plc-2 encodes one of five C. elegans phospholipase C (PLC) isozymes; the amino acid sequence of PLC-2 is most closely related to members of the PLC-beta group, but is divergent and does not include an extended C terminus typical of other PLC-betas.	60.99	135.80	85.48	79.40	83.69	8.70E-06
poml-1	The E01A2.7 gene encodes one of five paraoxonase-like proteins; it is homologous to the human genes PARAOXONASE 1 gene (PON1, OMIM:168820) and PARAOXONASE 2 (OMIM:602447), which in some allelic forms are associated with susceptibility to coronary artery disease or to variations in fasting plasma glucose.	137.23	51.64	37.24	36.34	31.30	1.93E-04
ppat-1	ppat-1 is an ortholog of human PPAT (phosphoribosyl pyrophosphate amidotransferase); ppat-1 is predicted to have amidophosphoribosyltransferase activity, based on protein domain information.	150.32	73.56	414.83	434.64	281.86	2.80E-06
psd-1	psd-1 is an ortholog of human PISD (phosphatidylserine decarboxylase); psd-1 is predicted to have phosphatidylserine decarboxylase activity, based on protein domain information.	274.56	424.58	327.64	480.98	570.80	7.80E-06
ptr-14	ptr-14 encodes a nematode-specific member of the sterol sensing domain (SSD) proteins, distantly paralogous to Drosophila PATCHED (PTC) and human PTCH (OMIM:601309, mutated in basal cell nevus syndrome); PTR-14 is weakly required for normal molting from L4 to adult stages; PTR-14 is also required for normal growth to full size and locomotion.	27.34	26.12	44.59	44.84	34.40	2.41E-04
R06C1.6	R06C1.6	12.87	19.47	14.14	13.37	11.66	1.04E-05
R07E3.4	R07E3.4 is an ortholog of human SLC25A6 (solute carrier family 25 (mitochondrial carrier; adenine nucleotide translocator), member 6), SLC25A4 (solute carrier family 25 (mitochondrial carrier; adenine nucleotide translocator), member 4) and SLC25A5 (solute carrier family 25 (mitochondrial carrier; adenine nucleotide translocator), member 5); R07E3.4 is predicted to have transporter activity, based on protein domain information.	100.10	45.98	576.42	515.92	335.71	1.82E-05
R07E5.15	R07E5.15	791.27	382.84	298.95	154.00	144.58	1.05E-04
R07G3.8	R07G3.8 is an ortholog of human FAM49A (family with sequence similarity 49, member A) and FAM49B (family with sequence similarity 49, member B).	77.20	182.33	108.00	113.88	106.19	1.49E-04
R08A2.1	R08A2.1	67.96	47.23	41.82	36.56	39.83	1.39E-04
R08A2.2 /// R08C7.8	R08A2.2 is an ortholog of human PPP1CC (protein phosphatase 1, catalytic subunit, gamma isozyme) and PPP1CA (protein phosphatase 1, catalytic subunit, alpha isozyme); R08A2.2 is predicted to have hydrolase activity, based on protein domain information. R08C7.8 is an ortholog of human PPP1CC (protein phosphatase 1, catalytic subunit, gamma isozyme) and PPP1CA (protein phosphatase 1, catalytic subunit, alpha isozyme); R08C7.8 is predicted to have hydrolase activity,	15.35	9.96	9.99	8.81	9.62	3.91E-05

	based on protein domain information.						
R09E10.6	R09E10.6 encodes a novel protein conserved amongst nematodes; R09E10.6 expression is enriched in the germline and loss of R09E10.6 activity via RNAi results in germ cell abnormalities, including oogenesis defects and abnormal syncytial germ cell compartments, that lead to reduced fertility.	743.75	388.03	262.82	123.14	111.12	4.70E-05
R102.8	R102.8	17.97	12.30	11.64	10.70	10.76	1.96E-04
R10D12.10	R10D12.10 is an ortholog of human TTBK2 (tau tubulin kinase 2) and TTBK1 (tau tubulin kinase 1); R10D12.10 is predicted to have protein kinase activity and ATP binding activity, based on protein domain information.	28.72	22.54	19.86	19.48	19.74	2.54E-04
R10E9.2	R10E9.2	892.45	428.11	271.12	141.92	126.23	6.32E-05
ras-1	ras-1 is orthologous to the human oncogene, Related Ras Viral Oncogene Homolog 2 (RRAS2).	62.89	166.93	87.56	79.19	72.29	1.91E-04
rfs-1	rfs-1 encodes a RAD51-like protein, orthologous to mammalian RAD-51C, that is related to bacterial recA, a multifunctional enzyme involved in DNA repair, homologous recombination, and induction of the SOS response; RFS-1 interacts with RAD-51, a C. elegans RAD51 homolog involved in meiosis and DNA repair, suggesting that RFS-1 may also play a role in these processes.	114.88	430.63	167.21	137.50	139.21	3.00E-06
rhi-1	rhi-1 encodes a Rho GDP-dissociation inhibitor (GDI).	221.80	489.38	368.60	324.66	312.06	1.94E-04
ric-19	The ric-19 gene encodes an evolutionarily conserved cytosolic protein involved in neuroendocrine secretion via association with secretory vesicles.	27.83	113.03	56.96	62.29	61.24	3.00E-07
rig-1	rig-1 is an ortholog of human BOC (BOC cell adhesion associated, oncogene regulated) and TMIGD1 (transmembrane and immunoglobulin domain containing 1).	82.61	119.03	114.06	124.07	100.12	2.16E-04
rmd-3	rmd-3 is an ortholog of human RMDN2 (regulator of microtubule dynamics 2) and RMDN3 (regulator of microtubule dynamics 3).	267.55	137.96	92.40	48.62	39.40	1.80E-04
rmd-4 /// rmd-6	rmd-4 is an ortholog of human RMDN1 (regulator of microtubule dynamics 1), RMDN2 (regulator of microtubule dynamics 2) and RMDN3 (regulator of microtubule dynamics 3). rmd-6 is an ortholog of human RMDN2 (regulator of microtubule dynamics 2) and RMDN3 (regulator of microtubule dynamics 3).	307.16	158.25	111.99	47.96	40.07	9.99E-05
rnh-1.1	rnh-1.1 is one of four C. elegans genes that can encode an RNase H ribonuclease; when expressed in vitro, RNH-1.1 exhibits RNase H activity; rnh-1.1 transcripts are expressed from the L3 larval stage through adulthood.	60.17	27.21	25.70	21.05	18.07	5.60E-06
rol-1	rol-1 encodes a nematode cuticular collagen required for normal body morphology at the adult stage of development; rol-1 expression is under the control of the heterochronic pathway, as heterochronic mutants that synthesize adult cuticle early produce animals that roll as larvae, while heterochronic mutants that fail to execute normal adult development never display the roller phenotype.	110.00	66.30	63.60	63.20	65.65	2.23E-04
rsd-3	rsd-3 is an ortholog of human CLINT1 (clathrin interactor 1).	178.93	294.45	229.03	238.73	190.26	9.00E-05
sax-1	The sax-1 gene encodes a homolog of Ndr kinase that regulates neuronal cell shape and neurite initiation.	160.18	384.90	149.48	158.61	175.64	5.70E-06
scav-4	scav-4 is an ortholog of human CD36 (CD36 molecule (thrombospondin receptor)) and SCARB2 (scavenger receptor class B, member 2).	186.10	85.16	213.99	299.23	307.72	1.45E-05
scrm-7	scrm-7 encodes a putative phospholipid scramblase homologous to human PLSCR1-5, and paralogous to other C. elegans SCRMs (e.g., SCRM-1); scrm-7(RNAi) animals have no obvious phenotype.	30.54	20.90	19.61	19.27	20.54	3.93E-05
sek-4	sek-4 is an ortholog of human MAP2K7 (mitogen-activated protein kinase kinase 7); sek-4 is predicted to have protein kinase activity and ATP binding activity, based on protein domain information.	40.79	113.63	54.53	51.13	61.60	4.40E-06
siah-1	siah-1 is an ortholog of human SIAH2 (siah E3 ubiquitin protein ligase 2), SIAH3 (siah E3 ubiquitin protein ligase family member 3) and SIAH1 (siah E3 ubiquitin protein ligase 1); siah-1 is predicted to have ubiquitin-protein transferase activity and zinc ion binding activity, based on protein domain information.	19.31	121.69	21.62	19.87	21.75	1.64E-05
sir-2.3	sir-2.3 encodes one of four C. elegans proteins with similarity to the Saccharomyces cerevisiae	13.84	12.76	25.83	57.43	67.66	1.34E-04

	Sir2p NAD-dependent histone deacetylase.						
skr-19	SKp1 Related (ubiquitin ligase complex component): The skr-19 gene encodes a homolog of Skp1 in S. cerevisiae that has no known function in vivo, since skr-19(RNAi) animals are at least superficially normal.	97.22	335.30	198.44	184.48	160.63	1.87E-04
slc-17.5	slc-17.5 is an ortholog of human SLC37A1 (solute carrier family 37 (glucose-6-phosphate transporter), member 1), SLC37A2 (solute carrier family 37 (glucose-6-phosphate transporter), member 2), SLC37A3 (solute carrier family 37, member 3), SLC17A4 (solute carrier family 17, member 4) and SLC17A1 (solute carrier family 17 (organic anion transporter), member 1).	86.48	57.06	380.87	272.57	182.69	1.00E-07
smd-1	smd-1 encodes an S-adenosylmethionine decarboxylase; SMD-1 functions in polyamine biosynthesis, exhibiting adenosylmethionine decarboxylase activity in vitro that is stimulated by putrescine; in large-scale RNAi screens, loss of smd-1 results in defective axon guidance and, in a sensitized genetic background, locomotion defects; smd-1::gfp reporter fusions are expressed in the intestine and in head and tail neurons; in males, there is also additional smd-1::gfp expression in ray neurons.	123.54	34.62	109.95	110.30	123.41	2.45E-04
smz-2	smz-2 encodes a PDZ domain-containing protein; SMZ-2 activity is required for spermatocytes to progress through meiotic divisions, and specifically for meiotic chromosome segregation; SMZ-2 localizes to spermatogenic meiotic chromosomes and mature sperm chromatin.	281.87	135.00	110.86	62.15	64.68	1.93E-04
snb-2	snb-2 is an ortholog of human VAMP5 (vesicle-associated membrane protein 5), VAMP3 (vesicle-associated membrane protein 3) and VAMP1 (vesicle-associated membrane protein 1 (synaptobrevin 1)).	162.08	585.76	332.76	289.78	287.10	6.65E-05
snb-5	snb-5 encodes a predicted synaptobrevin.	45.44	30.63	25.22	21.22	21.75	1.77E-04
snf-5	snf-5 is an ortholog of human SLC6A5 (solute carrier family 6 (neurotransmitter transporter), member 5) and SLC6A7 (solute carrier family 6 (neurotransmitter transporter), member 7); snf-5 is predicted to have neurotransmitter:sodium symporter activity, based on protein domain information.	127.72	49.27	42.98	38.84	36.16	3.23E-05
sodh-1	sodh-1 is predicted to have zinc ion binding activity and oxidoreductase activity, based on protein domain information.	94.67	4854.64	1625.56	704.26	354.94	1.77E-04
sox-2	sox-2 encodes, by alternative splicing, three isoforms of a putative HMG-box transcription factor orthologous to human SOX1 (OMIM:602148), SOX2 (OMIM:184429, mutated in syndromic anophthalmia), and SOX3 (OMIM:313430, mutated in hypopituitarism), and paralogous to SOX-3; SOX-2 is required for normal embryonic and larval viability, fertility, egg-laying, locomotion, and normally rapid growth; SOX-2 is expressed in larval and adult hypodermis and neurons; the sox-2 gene contains a predicted pan-neuronal regulatory motif.	60.08	196.58	146.55	146.73	124.58	4.03E-05
spe-11	spe-11 encodes a novel protein that is required for early embryonic development and for regulating the dynamic morphology of sperm pseudopods; SPE-11 is one of the few paternally provided proteins known to be essential for embryogenesis; SPE-11 is first detected in the nuclei of primary spermatocytes and then remains tightly associated with sperm chromatin until fertilization at which point it appears to be degraded.	173.73	89.86	70.79	27.36	28.36	9.42E-05
spe-46	defective SPErmatogenesis	108.71	56.73	40.50	28.47	30.05	1.24E-05
spp-10	spp-10 encodes two protein isoforms that are orthologous to the human gene PROSAPOSIN (PSAP; OMIM:176801, mutated in Gaucher disease and metachromatic leukodystrophy); SPP-10A and -10B are predicted to have 3 and 4 embedded saposin sequences, that are likely to be split into individual saposin peptides by proteolysis; in mammals, saposins activate the enzymes sphingomyelinphosphodiesterase and beta-glucosylceramidase; spp-10 has no obvious function in mass RNAi assays.	1423.07	2691.36	2499.11	2356.79	2335.84	1.43E-04
spp-23	spp-23	232.28	85.58	506.28	726.86	564.21	9.00E-06
spp-8	spp-8 is an ortholog of human PSAPL1 (prosaposin-like 1 (gene/pseudogene)) and PSAP (prosaposin).	288.41	1787.57	623.29	427.34	421.45	1.02E-04
sptl-2	sptl-2 is an ortholog of human SPTLC3 (serine palmitoyltransferase, long chain base subunit 3) and SPTLC2 (serine palmitoyltransferase, long chain base subunit 2); sptl-2 is predicted to have catalytic activity and pyridoxal phosphate binding activity, based on protein domain information.	325.34	91.77	1107.79	1352.42	1284.26	3.50E-06

sqst-1	sqst-1 encodes a protein with similarity to mammalian sequestosome 1(SQSTM1)/p62, a signal transduction or adaptor protein involved in receptor-mediated signaling pathways; SQST-1 is weakly expressed in embryos and exhibits a diffuse, cytoplasmic localization; SQST-1 is selectively removed by autophagy during embryogenesis.	182.69	1263.56	381.32	363.45	366.18	1.57E-05
sqst-2	sqst-2 is an ortholog of human SQSTM1 (sequestosome 1); sqst-2 is predicted to have zinc ion binding activity, based on protein domain information.	99.99	204.57	617.49	448.27	265.01	2.25E-04
sri-24	7TM GPCR, serpentine chemoreceptor class i (Sri)	10.12	7.95	9.56	10.39	9.61	9.15E-05
srr-6	7TM GPCR, serpentine receptor class r (Str)	10.42	143.79	10.56	9.51	12.08	1.00E-07
ssp-31	Sperm-specific class P protein 31	294.43	207.71	148.52	78.92	62.28	1.50E-04
ssp-33	Sperm Specific family, class P	200.84	130.80	82.33	43.66	44.46	9.82E-05
ssq-1	ssq-1 encodes a member of the sperm-specific, class Q family of proteins; about 5% of ssq-1 transcripts are RNA edited to change A to G resulting in a change to residue 127 from an G to a G.	771.27	402.40	280.23	165.29	142.35	2.33E-04
ssq-3	Sperm-Specific family, class Q	490.62	198.05	124.59	83.18	76.32	2.07E-04
sss-1	sss-1	786.67	309.32	179.76	99.09	82.31	8.80E-06
sss-2	Sperm-Specific family, class S	291.07	102.19	82.02	62.62	65.67	3.69E-05
sta-1	sta-1 encodes a protein that is a member of the STAT family of transcription factors; from N- to C-terminus, STA-1 contains conserved coiled-coil, DNA-binding, and SH2 domains, but apparently lacks a conserved amino-terminal oligomerization domain found in other STAT family members; sta-1 activity is required for repressing dauer formation at high temperatures and genetic analyses indicate that STA-1 acts redundantly with some members of the DAF-7/TGF-beta signaling pathway to repress dauer formation, particularly at low temperatures; tyrosine-phosphorylated STA-1 is able to bind a high affinity mammalian STAT binding sequence, and the STA-1 C-terminus can function as a transcriptional activation domain; sta-1 is widely expressed during most life stages, including the dauer stage, and is found in the pharynx, intestine, body wall muscles, and in neurons; STA-1 localizes to both the cytoplasm and the nucleus, with expression in the latter found particularly in some amphid neurons; in some neurons, STA-1 expression appears to be negatively regulated by DAF-7/TGF-beta signaling.	14.56	49.66	43.78	41.39	34.10	1.84E-04
sto-1	sto-1 is an ortholog of human NPHS2 (nephrosis 2, idiopathic, steroid-resistant (podocin)).	88.79	256.88	181.31	200.23	156.08	1.80E-04
str-56	Seven TM Receptor	6.47	6.32	7.94	6.75	6.80	1.83E-04
sur-5	SUppressor of activated let-60 Ras	962.91	211.35	1200.58	2141.24	1623.10	1.00E-07
svh-2	Suppressor of VHp-1 deletion lethality: svh-2 is an ortholog of human MERTK (MER proto-oncogene, tyrosine kinase), TYRO3 (TYRO3 protein tyrosine kinase), AXL (AXL receptor tyrosine kinase), MST1R (macrophage stimulating 1 receptor (c-met-related tyrosine kinase)) and MET (MET proto-oncogene, receptor tyrosine kinase); svh-2 is predicted to have protein tyrosine kinase activity, based on protein domain information.	14.49	36.42	19.79	18.31	20.03	1.90E-04
T01B10.5	T01B10.5	12.45	61.17	16.33	15.53	15.43	1.00E-07
T01E8.1	T01E8.1 is an ortholog of human FBXL18 (F-box and leucine-rich repeat protein 18) and FBXL12 (F-box and leucine-rich repeat protein 12).	74.67	175.75	117.61	73.15	71.37	1.95E-04
T01H8.2	T01H8.2 is an ortholog of human SRR (serine racemase).	142.93	64.60	262.51	298.90	231.40	4.72E-05
T02E1.7	T02E1.7 is an ortholog of human SURF4 (surfeit 4).	57.35	19.52	21.82	19.39	18.07	2.00E-06
T02E9.5	T02E9.5	47.09	198.30	124.40	186.68	134.74	1.89E-04
T04F8.7	T04F8.7	62.63	252.29	71.98	63.30	66.48	1.23E-05
T05A8.3	T05A8.3	96.94	442.94	393.27	312.53	290.13	1.22E-04
T05C12.1	T05C12.1 is an ortholog of human TTBK2 (tau tubulin kinase 2) and TTBK1 (tau tubulin kinase 1); T05C12.1 is predicted to have protein kinase activity and ATP binding activity, based on protein domain information.	23.38	19.47	14.62	13.32	11.32	5.34E-05
T07A5.1	T07A5.1 is an ortholog of human FKTN (fukutin).	39.83	8.82	14.36	12.16	11.48	5.36E-05
T07E3.4	This gene encodes a protein containing an F-box, a motif predicted to mediate protein-protein interactions either with homologs of yeast Skp-1p or with other proteins.	187.18	64.56	195.64	192.58	143.24	2.44E-04

T08B6.4	T08B6.4	145.22	62.09	52.65	36.10	35.04	3.76E-05
T08B6.9	T08B6.9	95.95	44.12	31.95	28.42	29.80	1.21E-04
T08G11.2	T08G11.2 encodes a nematode-specific sperm protein, required for a normally high ovulation rate, that interacts with EGL-32; despite not being EGL-32, and although T08G11.2(tm336) complements egl-32(n155), T08G11.2 can partially rescue egl-32 mutants when expressed transgenically, implying that T08G11.2 can be a non-orthologous replacement for EGL-32 function in vivo; T08G11.2 has an SH2 motif, but this motif lacks a critical arginine residue; T08G11.2 has no obvious non-nematode homologs, but is paralogous to four other C. elegans proteins (B0207.11, F42G4.6, F44F4.10, and Y81G3A.1); T08G11.2(tm336) hermaphrodites have a lowered ovulation rate (and thus a lowered rate of egg-laying), retaining significantly fewer eggs than wild-type, yet also retaining late-stage embryos; T08G11.2 has no obvious phenotype in mass RNAi experiments, possibly because of genetic redundancy with its paralogs; T08G11.2 expression is strongly enriched in spermatogenesis.	81.32	35.60	33.00	24.46	25.10	1.34E-04
T08H10.1	T08H10.1 is predicted to have oxidoreductase activity, based on protein domain information.	1419.47	1156.70	811.55	607.63	586.01	3.52E-05
T10E9.4	T10E9.4	382.49	126.28	79.52	52.19	36.15	7.63E-05
T10E9.6	T10E9.6	34.37	17.91	18.58	13.68	14.14	2.20E-04
T10G3.3	T10G3.3	93.50	22.39	115.34	146.80	116.66	2.10E-06
T11G6.4	T11G6.4 is an ortholog of human SLC37A3 (solute carrier family 37, member 3); T11G6.4 is predicted to have transporter activity, based on protein domain information.	106.02	83.13	120.32	130.31	120.44	1.25E-04
T12A7.6	T12A7.6	23.82	91.57	30.42	25.91	27.62	2.00E-07
T12B3.3	T12B3.3 is an ortholog of human GDE1 (glycerophosphodiester phosphodiesterase 1); T12B3.3 is predicted to have glycerophosphodiester phosphodiesterase activity, based on protein domain information.	417.90	1350.03	1426.72	1302.26	1229.19	4.55E-05
T16A9.5	T16A9.5	557.91	204.88	149.51	69.30	55.84	1.81E-05
T16A9.5 /// Y69E1A.1	T16A9.5 /// Y69E1A.1	426.62	155.41	101.30	54.53	41.99	1.17E-04
T16G1.4	T16G1.4 is predicted to have transferase activity, transferring phosphorus-containing groups, based on protein domain information.	13.48	200.50	16.78	13.06	14.37	7.90E-06
T16G12.7	T16G12.7 is predicted to have hydrolase activity, based on protein domain information.	42.03	25.89	25.59	22.81	24.08	2.22E-04
T18D3.7	T18D3.7 is an ortholog of human TSC22D3 (TSC22 domain 3); T18D3.7 is predicted to have sequence-specific DNA binding transcription factor activity, based on protein domain information.	43.86	240.41	52.22	45.07	54.10	2.80E-06
T18D3.8	dead and merged to T18D3.7 is an ortholog of human TSC22D3 (TSC22 domain 3); T18D3.7 is predicted to have sequence-specific DNA binding transcription factor activity, based on protein domain information.	70.69	220.95	86.75	74.78	82.83	6.18E-05
T19C3.4	T19C3.4 is an ortholog of human REEP4 (receptor accessory protein 4), REEP3 (receptor accessory protein 3), REEP1 (receptor accessory protein 1) and REEP2 (receptor accessory protein 2).	32.23	88.86	40.20	56.64	55.45	6.40E-05
T19C4.5	T19C4.5 is predicted to have iron ion binding activity, oxygen binding activity, and heme binding activity, based on protein domain information.	22.39	57.62	26.26	27.30	28.32	1.38E-04
T19H5.4	T19H5.4	300.43	924.08	354.48	330.38	336.20	6.40E-06
T20H4.2	T20H4.2	37.92	25.14	21.92	17.91	20.51	2.29E-04
T21F4.1	T21F4.1 is orthologous to the human gene Arginase Type I Erythroid Variant (ARG1), which contributes most of the arginase activity in the liver.	8.93	21.64	38.58	22.77	20.46	1.31E-04
T22B3.3	Major sperm protein	275.86	152.34	107.04	67.51	66.47	1.72E-04
T22H2.4	T22H2.4	86.56	85.89	124.38	131.59	124.69	8.01E-05
T23F11.2	T23F11.2	540.35	242.66	162.87	82.81	85.69	1.86E-04
T23G11.1	T23G11.1	580.89	282.65	178.64	84.72	62.20	3.17E-05
T25B9.1	T25B9.1 encodes an protein highly similar to 2-amino-3-keto-butyrate coenzyme A ligases (also called AKB ligases or glycine acetyltransferases; EC 2.3.1.29; R.W.	332.11	87.59	103.40	142.38	143.91	2.01E-04

T25B9.9	T25B9.9 is an ortholog of human PGD (phosphogluconate dehydrogenase); T25B9.9 is predicted to have phosphogluconate dehydrogenase (decarboxylating) activity and NADP binding activity, based on protein domain information.	888.00	429.62	770.80	1199.91	1485.21	1.20E-06
T27A3.5	T27A3.5 is an ortholog of human PTPN22 (protein tyrosine phosphatase, non-receptor type 22 (lymphoid)), PTPN18 (protein tyrosine phosphatase, non-receptor type 18 (brain-derived)) and PTPN12 (protein tyrosine phosphatase, non-receptor type 12); T27A3.5 is predicted to have protein tyrosine phosphatase activity, based on protein domain information.	144.49	58.03	45.66	32.32	35.24	2.03E-04
T27E4.7	T27E4.7	61.82	292.72	87.89	111.74	113.68	1.33E-05
T27E7.1	T27E7.1	413.58	167.18	128.24	64.67	72.86	7.17E-05
T28B8.1	T28B8.1	153.49	472.74	143.85	156.59	163.97	9.67E-05
T28C6.8	T28C6.8	132.50	206.90	296.85	453.89	454.99	3.71E-05
T28D6.3	T28D6.3	250.93	49.87	67.65	86.17	90.97	8.64E-05
T28H11.7 /// ZC477.7	non-coding Transcript Isoform	435.92	198.41	146.65	83.04	63.42	6.95E-05
tag-234	tag-234	24.81	204.06	54.97	44.19	36.23	2.00E-07
tag-243	tag-243 is an ortholog of human ABRA (actin-binding Rho activating protein).	82.95	311.23	158.71	140.38	130.44	7.51E-05
tag-290	tag-290 is an ortholog of human TFPI (tissue factor pathway inhibitor (lipoprotein-associated coagulation inhibitor)) and TFPI2 (tissue factor pathway inhibitor 2); tag-290 is predicted to have serine-type endopeptidase inhibitor activity, based on protein domain information.	31.88	20.42	22.86	22.80	24.77	3.61E-05
tag-304	tag-304 is an ortholog of human GID8 (GID complex subunit 8); tag-304 is localized to the nucleus.	109.33	216.25	99.36	108.44	104.24	1.04E-04
tdo-2	tdo-2 is an ortholog of human TDO2 (tryptophan 2,3-dioxygenase); tdo-2 is predicted to have tryptophan 2,3-dioxygenase activity and heme binding activity, based on protein domain information.	391.41	162.89	576.99	501.67	379.15	2.25E-04
toh-1	toh-1 encodes an astacin-like metalloprotease; TOH-1 is predicted to function as a secreted protease; experiments that specifically assessed toh-1's role in molting indicate that toh-1(RNAi) causes no abnormal phenotypes.	14.28	10.87	9.43	11.18	11.78	1.19E-04
tps-1	tps-1 is predicted to have catalytic activity, based on protein domain information.	40.07	81.97	50.05	37.75	35.75	2.14E-04
trap-2	trap-2 is an ortholog of human SSR2 (signal sequence receptor, beta (translocon-associated protein beta)).	822.95	362.15	407.62	638.65	730.80	1.01E-04
tre-3	tre-3 encodes one of four putative trehalases in C. elegans; tre-3 is expressed throughout development.	88.09	202.53	310.97	288.09	237.95	4.30E-06
tre-5	tre-5 is an ortholog of human TREH (trehalase (brush-border membrane glycoprotein)); tre-5 is predicted to have alpha,alpha-trehalase activity, based on protein domain information.	32.41	150.84	42.21	37.04	38.38	4.15E-05
trx-3 (thioredoxin)	trx-3 is an ortholog of human NXNL1 (nucleoredoxin-like 1) and NXNL2 (nucleoredoxin-like 2); trx-3 is localized to the nucleus, the cytoplasm and the apical plasma membrane.	69.91	507.62	69.61	81.56	113.74	5.24E-05
tsp-19	tsp-19 is an ortholog of human CD63 (CD63 molecule).	13.42	9.07	9.19	8.94	9.04	1.20E-06
tth-1	tth-1 encodes a thymosin beta ortholog that contains four functionally distinct thymosin beta repeats; in vitro, TTH-1 binds multiple actin monomers as well as filamentous actin suggesting that, as its homology predicts, TTH-1 plays a direct role in regulating actin polymerization and cytoskeletal organization in C. elegans; consistent with this, loss of tth-1 activity via a deletion mutation that removes upstream sequences results in animals with a dumpy morphology that are sterile as a result of maternal effect lethality associated with deformed oocytes containing abnormally distributed actin; tth-1 mRNA is present throughout development, while TTH-1 protein is detected in the adult gonad in the inside edges of the membranous structures surrounding germline nuclei, the cytoplasm and cortex of oocytes, early embryos at points of cell contact, the developing nerve ring, and then throughout the larval and adult body, with specific enrichment in the intestine and spermatheca.	143.02	693.13	572.89	426.76	330.63	1.23E-05
ttr-26	TransThyretin-Related family domain	41.85	258.87	233.56	162.20	142.56	1.09E-04
ttr-27	TransThyretin-Related family domain	19.36	15.29	13.51	10.53	13.61	8.98E-05

ttr-37	ttr-37	30.97	199.97	34.33	29.01	36.18	2.60E-06
ttr-5	C40H1.5 encodes a protein containing a predicted signal sequence followed by a transthyretin-like domain; the product of C40H1.5 belongs to a family of apparently nematode-specific proteins whose function is not yet known.	217.18	887.49	801.84	629.06	582.39	5.23E-05
twk-7	twk-7 is an ortholog of human KCNK18 (potassium channel, two pore domain subfamily K, member 18), KCNK5 (potassium channel, two pore domain subfamily K, member 5) and KCNK17 (potassium channel, two pore domain subfamily K, member 17); twk-7 is predicted to have potassium channel activity, based on protein domain information.	20.36	44.35	31.44	23.40	24.54	1.61E-04
ugt-21	ugt-21 is an ortholog of human UGT3A2 (UDP glycosyltransferase 3 family, polypeptide A2) and UGT3A1 (UDP glycosyltransferase 3 family, polypeptide A1); ugt-21 is predicted to have transferase activity, transferring hexosyl groups, based on protein domain information.	64.68	32.01	135.64	136.73	143.86	1.19E-05
ugt-22	ugt-22 is an ortholog of human UGT3A2 (UDP glycosyltransferase 3 family, polypeptide A2) and UGT3A1 (UDP glycosyltransferase 3 family, polypeptide A1); ugt-22 is predicted to have transferase activity, transferring hexosyl groups, based on protein domain information.	939.85	122.44	337.97	610.57	711.79	3.00E-05
ugt-46	ugt-46 is predicted to have transferase activity, transferring hexosyl groups, based on protein domain information.	548.08	183.49	519.71	703.85	658.15	1.69E-04
ugt-48	ugt-48 is predicted to have transferase activity, transferring hexosyl groups, based on protein domain information.	50.96	29.43	90.32	89.14	81.91	2.46E-04
ugt-54	ugt-54 is an ortholog of human UGT2A3 (UDP glucuronosyltransferase 2 family, polypeptide A3), UGT2B17 (UDP glucuronosyltransferase 2 family, polypeptide B17), UGT2A1 (UDP glucuronosyltransferase 2 family, polypeptide A1, complex locus), UGT2B4 (UDP glucuronosyltransferase 2 family, polypeptide B4) and UGT2A2 (UDP glucuronosyltransferase 2 family, polypeptide A2); ugt-54 is predicted to have transferase activity, transferring hexosyl groups, based on protein domain information.	13.92	60.54	42.34	23.00	19.20	5.70E-06
ugt-63	ugt-63 is predicted to have transferase activity, transferring hexosyl groups, based on protein domain information.	143.22	10.64	173.26	148.23	90.02	5.80E-06
vab-9	vab-9 encodes a claudin homolog orthologous to human brain cell membrane protein 1 (BCMP1) and Drosophila CG6982; VAB-9 colocalizes with HMP-1 to an apical layer of the adherens junctions of all epithelial cells, one layer more apically than AJM-1; vab-9 mutants have disorganized F-actin at the adherens junction, implying that VAB-9 links junctions to circumferential actin filaments; VAB-9 is also expressed in the nerve ring; VAB-9 requires the cadherin HMR-1 to localize to the cell membrane, and both alpha-catenin (HMP-1) and beta-catenin (HMP-2) to remain at the cell junction after membrane localization.	70.24	144.61	122.19	128.43	114.96	2.25E-04
VC5.2	VC5.2 is an ortholog of human LAMA2 (laminin, alpha 2) and LAMA1 (laminin, alpha 1).	63.18	124.81	78.81	60.71	52.90	1.14E-04
vha-12	vha-12 encodes an ortholog of subunit B of the cytoplasmic (V1) domain of vacuolar proton-translocating ATPase (V-ATPase); VHA-12 is orthologous to human ATP6V1B1 (OMIM:192132, mutated in distal renal tubular acidosis with progressive sensorineural deafness) and ATP6V1B2 (OMIM:606939); VHA-12 and TAG-300/Y110A7A.12 are co-orthologs; VHA-17, like VHA-1 and VHA-12, antagonizes EFF-1-mediated cell fusion in hypodermal cells; VHA-12 is required for necrosis, since mutation of vha-12 suppresses necrotic neurodegeneration, cytoplasmic acidification, and thapsigargin-induced cell death; VHA-12 is predicted to bind ATP as part of a cytosolic VHA-12/VHA-13 heterohexamer, whose ATP hydrolysis drives the V-ATPase rotor; general levels of VHA-12 protein are very low in embryos but increase strongly after hatching.	2302.02	944.00	991.39	1222.65	1328.98	7.14E-05
vha-14	vha-14 encodes an ortholog of subunit D of the cytoplasmic (V1) domain of vacuolar proton-translocating ATPase (V-ATPase); VHA-14 is a predicted cytosolic rotor (stalk) component.	2136.11	1612.41	1447.20	1499.22	1685.90	2.19E-04
W01B6.6	W01B6.6 is predicted to have protein tyrosine phosphatase activity, based on protein domain information.	15.56	9.96	9.48	8.52	9.32	2.60E-05
W01C8.5	W01C8.5 is an ortholog of human THBD (thrombomodulin).	321.13	378.16	1079.04	1406.41	1125.81	5.90E-06
W01D2.3	W01D2.3 is an ortholog of human REEP6 (receptor accessory protein 6) and REEP5 (receptor accessory protein 5).	64.19	34.36	32.04	26.13	27.29	1.13E-04
W02C12.2	W02C12.2	45.96	101.61	58.81	59.27	51.06	1.84E-04

W02D7.8	W02D7.8	19.48	60.93	38.47	38.25	31.86	1.98E-04
W03D8.10 /// W03D8.9	W03D8.10 /// W03D8.9	339.49	147.24	95.66	58.99	50.62	2.03E-04
W03D8.3	W03D8.3	46.65	26.21	24.43	19.24	24.78	2.48E-05
W03F11.1	W03F11.1 encodes a protein with three chitin-binding peritrophin-A domains; like CEJ-1 and CPG-2, W03F11.1 may participate in eggshell synthesis and early embryonic development; W03F11.1 is required for fertility in mass RNAi assays; W03F11.1's multiple peritrophin-A domains might enable mechanical cross-linking of chitin.	1901.05	5910.63	6243.49	5342.74	4972.29	1.50E-06
W08A12.2	W08A12.2	160.25	648.25	612.37	548.41	465.54	1.92E-05
W09B6.5	W09B6.5	16.99	11.68	34.21	36.38	26.48	1.59E-05
W09D6.4 /// Y47D3A.13	W09D6.4 /// Y47D3A.13	576.78	328.61	189.40	114.62	84.45	5.59E-05
W09G12.7	W09G12.7	39.04	174.43	106.01	50.73	56.22	6.06E-05
Y102A11A.3	Y102A11A.3	71.64	92.04	118.26	151.63	149.90	2.56E-04
Y105C5A.24	Y105C5A.24 is an ortholog of human MAP3K7 (mitogen-activated protein kinase kinase kinase 7); Y105C5A.24 is predicted to have protein kinase activity and ATP binding activity, based on protein domain information.	90.17	301.50	122.91	130.49	130.89	1.59E-05
Y105C5B.14	Y105C5B.14	27.40	21.51	46.74	55.90	42.03	7.28E-05
Y105E8B.7	Y105E8B.7 is an ortholog of human YEATS4 (YEATS domain containing 4).	46.14	193.72	98.57	94.41	81.55	1.21E-05
Y106G6D.3	Y106G6D.3	53.22	32.68	26.75	23.48	19.87	3.39E-05
Y11D7A.3	Y11D7A.3 is an ortholog of human MFSD11 (major facilitator superfamily domain containing 11).	287.91	101.59	223.63	201.74	183.81	1.28E-04
Y18D10A.21	Y18D10A.21	43.53	27.80	25.71	22.29	19.67	3.16E-05
Y22D7AR.6	Y22D7AR.6 is an ortholog of human IP6K3 (inositol hexakisphosphate kinase 3) and IP6K1 (inositol hexakisphosphate kinase 1); Y22D7AR.6 is predicted to have inositol-1,4,5-trisphosphate 3-kinase activity, based on protein domain information.	75.39	145.14	92.50	84.76	82.50	2.26E-04
Y23H5B.2 /// Y39G10AR.16	Y23H5B.2 /// Y39G10AR.16	22.89	17.90	17.35	15.41	16.19	5.48E-05
Y26D4A.21	Y26D4A.21	40.78	24.54	153.33	152.32	96.03	7.31E-05
Y34F4.1	Y34F4.1	37.34	157.77	158.24	143.36	122.48	2.15E-04
Y37D8A.16	Y37D8A.16	296.92	1034.15	756.04	824.85	668.39	1.17E-04
Y37D8A.19	Y37D8A.19	1953.56	3964.68	3542.45	3554.36	3598.70	3.50E-06
Y37F4.5	Y37F4.5	19.35	13.08	12.71	10.66	10.44	8.96E-05
Y38C1AA.7	Y38C1AA.7	581.23	272.18	169.44	98.51	72.67	1.38E-04
Y38E10A.17	Y38E10A.17	280.51	126.89	110.91	69.62	66.22	2.19E-04
Y39D8A.1	Y39D8A.1 is an ortholog of human MFSD11 (major facilitator superfamily domain containing 11).	29.21	10.76	65.35	53.01	36.92	5.37E-05
Y39G10AR.16	Y39G10AR.16	90.69	60.49	56.66	41.65	41.63	1.81E-05
Y40B10A.9	Y40B10A.9 is an ortholog of human PLB1 (phospholipase B1).	59.44	147.56	121.99	101.55	87.46	1.12E-04
Y41C4A.12	Y41C4A.12	62.89	61.31	153.73	135.20	123.34	8.00E-07
Y42G9A.1	Y42G9A.1	31.94	125.86	28.78	25.89	31.80	1.00E-07
Y43C5A.3	Y43C5A.3	93.58	749.70	494.72	368.47	236.65	2.04E-04
Y43C5B.2	Y43C5B.2 is an ortholog of human ABL2 (ABL proto-oncogene 2, non-receptor tyrosine kinase) and ABL1 (ABL proto-oncogene 1, non-receptor tyrosine kinase); Y43C5B.2 is predicted to have protein tyrosine kinase activity, based on protein domain information.	16.82	9.77	9.69	11.05	10.40	7.28E-05
Y43E12A.2	Y43E12A.2 is an ortholog of human ZCCHC24 (zinc finger, CCHC domain containing 24); Y43E12A.2 is localized to the mitochondrion.	77.89	253.68	67.67	66.86	65.00	1.40E-06

Y43F4B.5	Y43F4B.5 is an ortholog of human PGM2L1 (phosphoglucomutase 2-like 1) and PGM2 (phosphoglucomutase 2); Y43F4B.5 is predicted to have intramolecular transferase activity, phosphotransferases, based on protein domain information; Y43F4B.5 is localized to the nucleus.	885.06	553.69	977.57	1114.84	1026.07	1.28E-05
Y43F8B.9	Y43F8B.9	59.28	584.30	160.76	95.30	98.97	3.94E-05
Y44A6D.5	Y44A6D.5 is an ortholog of human BCAT2 (branched chain amino-acid transaminase 2, mitochondrial); Y44A6D.5 is predicted to have branched-chain-amino-acid transaminase activity, based on protein domain information.	81.27	49.68	42.07	38.69	32.25	6.75E-05
Y46G5A.15	Y46G5A.15	12.66	23.39	13.71	14.65	15.96	2.74E-05
Y47D7A.13	Y47D7A.13	176.21	45.36	43.32	43.92	48.07	6.09E-05
Y48A6B.7	Y48A6B.7 encodes a cytidine deaminase; by homology the product of Y48A6B.7 is predicted to function in deamination of cytidine to uracil.	318.40	1083.13	2148.14	1986.74	1459.61	2.58E-05
Y49E10.18	Y49E10.18	291.05	262.53	305.88	575.51	589.09	7.67E-05
Y49G5A.1	Y49G5A.1 is predicted to have serine-type endopeptidase inhibitor activity, based on protein domain information.	46.56	8.39	9.87	9.65	9.49	2.16E-04
Y4C6B.5	Y4C6B.5 is an ortholog of human SLC46A1 (solute carrier family 46 (folate transporter), member 1) and SLC46A3 (solute carrier family 46, member 3).	192.83	145.30	194.47	302.79	292.63	2.41E-05
Y50D4B.3 /// Y50D4B.4	Y50D4B.3 is an ortholog of human NGLY1 (N-glycanase 1). Y50D4B.4 is an ortholog of human NGLY1 (N-glycanase 1).	112.67	33.70	37.58	43.84	44.19	2.80E-06
Y51A2D.13	Y51A2D.13 is an ortholog of human PLD4 (phospholipase D 4), PLD5 (phospholipase D 5) and PLD3 (phospholipase D 3); Y51A2D.13 is predicted to have catalytic activity, based on protein domain information.	103.46	572.14	80.28	93.33	145.19	8.09E-05
Y51A2D.18	Y51A2D.18 is an ortholog of human SLC22A16 (solute carrier family 22 (organic cation/carnitine transporter), member 16), SLC22A4 (solute carrier family 22 (organic cation/zwitterion transporter), member 4) and SLC22A5 (solute carrier family 22 (organic cation/carnitine transporter), member 5); Y51A2D.18 is predicted to have transmembrane transporter activity, based on protein domain information.	148.56	36.76	64.49	85.13	81.89	2.35E-04
Y51B9A.5	Y51B9A.5	50.07	26.88	20.32	16.48	14.24	1.28E-04
Y51B9A.9	Y51B9A.9 is an ortholog of human MAPK8 (mitogen-activated protein kinase 8); Y51B9A.9 is predicted to have protein kinase activity and ATP binding activity, based on protein domain information.	25.62	101.91	64.70	51.07	55.66	5.58E-05
Y53F4B.19	Major sperm protein	64.56	33.25	32.00	26.26	23.43	2.17E-04
Y53G8AR.7	Y53G8AR.7 is an ortholog of human MFSD8 (major facilitator superfamily domain containing 8).	87.96	70.03	165.29	181.84	205.03	1.82E-04
Y53H1B.2	Y53H1B.2	330.05	1507.70	1210.46	1028.40	846.55	2.28E-04
Y54E2A.9	Y54E2A.9 is an ortholog of human VMO1 (vitelline membrane outer layer 1 (chicken)).	36.93	23.94	18.23	12.97	15.82	4.36E-05
Y54G11A.7	Y54G11A.7 is an ortholog of human TTC38 (tetratricopeptide repeat domain 38).	262.24	154.26	381.85	565.73	556.27	8.89E-05
Y54G2A.11	Y54G2A.11	67.90	245.12	63.46	66.56	67.33	5.30E-06
Y54G9A.4	Y54G9A.4 is an ortholog of human SLC39A2 (solute carrier family 39 (zinc transporter), member 2); Y54G9A.4 is predicted to have metal ion transmembrane transporter activity, based on protein domain information.	65.15	24.19	165.09	104.96	99.05	1.00E-07
Y57A10A.14	Y57A10A.14	62.30	139.96	36.16	57.75	67.92	2.02E-04
Y57A10A.26	Y57A10A.26 is an ortholog of human FAXC (failed axon connections (Drosophila)).	242.22	543.16	510.81	494.83	431.89	2.21E-04
Y57G11A.2	Y57G11A.2 is an ortholog of human VMO1 (vitelline membrane outer layer 1 (chicken)).	183.74	53.15	48.73	39.00	42.69	1.30E-04
Y57G11B.2	Y57G11B.2	24.86	83.82	39.34	25.13	25.64	9.63E-05
Y59E9AL.2 /// Y59E9AL.3	Y59E9AL.2 /// Y59E9AL.3	1141.65	537.24	321.52	141.83	102.78	1.18E-04
Y59E9AL.6	Y59E9AL.6	544.76	304.74	227.46	131.19	117.80	8.71E-05

Y59H11AM.2 /// Y59H11AM.3	mpst-5 is an ortholog of human TST (thiosulfate sulfurtransferase (rhodanese)) and MPST (mercaptopyruvate sulfurtransferase). mpst-6 is an ortholog of human TST (thiosulfate sulfurtransferase (rhodanese)) and MPST (mercaptopyruvate sulfurtransferase).	52.88	21.97	14.69	8.23	8.50	8.55E-05
Y62H9A.3	Y62H9A.3	1444.01	2780.94	2512.65	2309.34	2279.41	1.12E-04
Y67A6A.1	Y67A6A.1	14.58	9.97	9.69	8.55	8.16	6.10E-06
Y69A2AR.19	Y69A2AR.19 is an ortholog of human PTPN2 (protein tyrosine phosphatase, non-receptor type 2) and PTPN1 (protein tyrosine phosphatase, non-receptor type 1); Y69A2AR.19 is predicted to have protein tyrosine phosphatase activity, based on protein domain information.	29.37	14.41	15.17	14.45	13.05	8.22E-05
Y69E1A.8	Y69E1A.8	59.46	31.33	30.23	27.30	26.84	1.17E-04
Y6E2A.4	Y6E2A.4	20.36	55.85	22.96	22.46	22.43	1.47E-05
Y71G12A.4	Y71G12A.4 is an ortholog of human ABHD17A (abhydrolase domain containing 17A), ABHD17B (abhydrolase domain containing 17B) and ABHD17C (abhydrolase domain containing 17C).	21.11	14.87	15.37	14.03	16.85	6.07E-05
Y71G12B.27	Y71G12B.27 is an ortholog of human CKS2 (CDC28 protein kinase regulatory subunit 2); Y71G12B.27 is predicted to have cyclin-dependent protein serine/threonine kinase regulator activity, based on protein domain information.	109.43	69.21	45.69	30.78	24.71	1.25E-04
Y73B6BL.14	Y73B6BL.14 is an ortholog of human LIG1 (ligase I, DNA, ATP-dependent); Y73B6BL.14 is predicted to have DNA binding activity and DNA ligase (ATP) activity, based on protein domain information.	50.76	25.97	36.11	32.97	37.94	2.11E-04
Y77E11A.14	Y77E11A.14	33.11	296.40	98.57	43.74	42.98	9.99E-05
Y7A5A.6	Y7A5A.6	32.72	92.84	97.93	63.38	58.07	6.80E-05
Y94H6A.12 /// Y94H6A.7	Y94H6A.12 is an ortholog of human PTRHD1 (peptidyl-tRNA hydrolase domain containing 1); Y94H6A.12 is predicted to have aminoacyl-tRNA hydrolase activity, based on protein domain information.	1030.05	1162.62	5837.25	6309.77	3463.96	1.88E-04
ZC373.2	ZC373.2	6468.78	10030.13	10246.94	9767.53	9966.86	2.14E-04
ZC443.3	ZC443.3 is an ortholog of human EIF3C (eukaryotic translation initiation factor 3, subunit C) and EIF3CL (eukaryotic translation initiation factor 3, subunit C-like).	21.96	305.29	37.91	50.05	36.77	1.00E-07
ZC477.2	ZC477.2 is an ortholog of human PPP1CB (protein phosphatase 1, catalytic subunit, beta isozyme); ZC477.2 is predicted to have hydrolase activity, based on protein domain information.	21.31	15.79	13.05	12.15	13.08	3.92E-05
zig-7	zig-7 encodes a predicted secreted protein that is a member of the immunoglobulin superfamily of proteins; loss of zig-7 activity via RNAi has been reported to result in aldicarb resistance; a zig-7::gfp reporter fusion is expressed in body wall muscle.	150.32	471.96	350.50	262.17	207.88	1.79E-04
zip-3	zip-3 encodes a bZip transcription factor.	314.60	155.74	428.35	414.96	421.13	7.67E-05
ZK1240.3	ZK1240.3 is an ortholog of human TRIM38 (tripartite motif containing 38), TRIM11 (tripartite motif containing 11), TRIM50 (tripartite motif containing 50), TRIM74 (tripartite motif containing 74) and TRIM73 (tripartite motif containing 73); ZK1240.3 is predicted to have zinc ion binding activity, based on protein domain information.	31.95	69.73	79.29	84.31	72.85	9.89E-05
ZK1248.17	ZK1248.17	205.52	151.72	103.95	66.87	56.57	2.59E-04
ZK1248.5	ZK1248.5	104.81	33.51	23.79	18.99	16.77	4.10E-06
ZK1307.1	ZK1307.1 is an ortholog of human DDAH2 (dimethylarginine dimethylaminohydrolase 2) and DDAH1 (dimethylarginine dimethylaminohydrolase 1); ZK1307.1 is predicted to have hydrolase activity, acting on carbon-nitrogen (but not peptide) bonds, in linear amidines, based on protein domain information.	436.50	440.48	968.81	1214.79	947.14	1.91E-04
ZK1307.3 /// ZK1307.4	ZK1307.3 /// ZK1307.4	490.14	303.77	186.25	113.11	88.52	6.56E-05
ZK185.3	ZK185.3	100.36	34.09	186.60	276.55	211.50	1.38E-05
ZK228.3	ZK228.3	155.19	25.35	223.85	252.70	225.20	1.77E-05
ZK228.4	ZK228.4 is involved in innate immune response.	218.60	33.23	476.68	563.67	486.35	5.37E-05
ZK262.4	ZK262.4	12.43	9.66	9.43	8.07	8.33	9.00E-06
ZK287.3	ZK287.3	148.52	340.18	219.58	197.72	183.59	7.07E-05
ZK354.7	Major sperm protein	141.19	81.08	70.10	38.10	42.26	1.12E-04

ZK418.2	non-coding Transcript Isoform	35.04	15.34	12.36	11.16	10.39	8.77E-05
ZK484.5	ZK484.5	1129.17	670.13	401.29	219.26	174.73	6.82E-05
ZK484.7	ZK484.7 is an ortholog of human PTPN22 (protein tyrosine phosphatase, non-receptor type 22 (lymphoid)), PTPN18 (protein tyrosine phosphatase, non-receptor type 18 (brain-derived)) and PTPN12 (protein tyrosine phosphatase, non-receptor type 12); ZK484.7 is predicted to have protein tyrosine phosphatase activity, based on protein domain information.	37.77	23.85	20.57	17.34	16.17	2.40E-04
ZK512.7	ZK512.7	693.16	123.43	452.31	542.65	470.00	4.80E-05
ZK550.5	ZK550.5 is an ortholog of human PHYH (phytanoyl-CoA 2-hydroxylase).	105.45	62.23	50.52	40.37	42.86	1.78E-04
ZK6.11	ZK6.11 is involved in innate immune response; ZK6.11 is localized to the membrane raft.	515.53	42.01	1107.33	1317.70	871.53	6.62E-05
ZK637.12	ZK637.12	41.85	25.98	23.15	19.06	20.32	1.80E-05
ZK673.2	ZK673.2 is an ortholog of human AK3 (adenylate kinase 3) and AK4 (adenylate kinase 4); ZK673.2 is predicted to have adenylate kinase activity and ATP binding activity, based on protein domain information.	664.49	1532.75	823.92	703.99	739.34	2.23E-05
ZK813.1	ZK813.1	4646.71	10314.13	9110.06	8731.97	8428.98	9.00E-07
ZK813.2	ZK813.2	903.78	3335.16	3135.54	2810.70	2463.45	2.32E-05
ZK813.3	ZK813.3	3364.09	6166.05	6399.17	6073.46	5861.10	5.10E-05
ZK813.7	ZK813.7	2201.00	4542.98	4325.09	4038.12	3783.60	3.01E-05
ZK84.2	ZK84.2 is predicted to have cholestenol delta-isomerase activity, based on protein domain information.	95.19	40.80	33.70	26.55	25.27	7.70E-06
ZK856.5	ZK856.5 is an ortholog of human SMPDL3A (sphingomyelin phosphodiesterase, acid-like 3A) and SMPDL3B (sphingomyelin phosphodiesterase, acid-like 3B); ZK856.5 is predicted to have hydrolase activity, based on protein domain information.	59.87	70.21	110.42	122.65	114.08	2.36E-05
ZK858.2	ZK858.2	169.26	89.64	52.68	29.37	27.09	8.21E-05
ZK899.1	ZK899.1	42.83	136.97	72.89	61.85	63.26	2.51E-04
ZK971.1	ZK971.1	40.47	95.50	107.88	88.35	76.28	2.39E-04

Supplemental Table S3: The genes whose expression was downregulated throughout the time course (Total 274 genes)

Gene symbols						
acdh-5	C28D4.5	col-173	F36A4.2	K06A1.2	str-56	Y57G11A.2
acdh-8	C31H1.1	col-180	F36A4.4	K06A5.2	T02E1.7	Y59E9AL.2 /// Y59E9AL.3
acox-2	C31H1.5	col-19	F36H12.8 /// R13H9.5	K06A5.3	T05C12.1	Y59E9AL.6
acs-18	C32E8.4	col-2	F36H12.9 /// R13H9.6	K07A1.4	T08B6.4	Y59H11AM.2 /// Y59H11AM.3
acs-6	C34B2.3	col-34	F37A4.4	K08C9.2	T08B6.9	Y67A6A.1
ant-1.4	C34D4.3	col-39	F37A4.5	K08F4.5	T08G11.2	Y69A2AR.19
asp-8	C34F11.2	col-43	F38H4.5	K09C4.5	T08H10.1	Y69E1A.8
B0218.7	C38C10.3	col-53	F40H3.2	K11C4.1	T10E9.4	Y71G12A.4
B0244.9	C39H7.1	col-62 /// col-7	F40H6.1	kin-21	T10E9.6	Y71G12B.27
B0252.5	C39H7.1 /// Y38H8A.3	col-66	F42A9.7	marc-2	T16A9.5	ZC477.2
B0261.6	C43E11.5 /// C50F2.5	col-71	F42C5.5	math-32	T16A9.5 /// Y69E1A.1	ZK1248.17
B0273.1	C47A4.3	comp-1	F44D12.8	mpst-4	T16G12.7	ZK1248.5
B0379.2	C47E12.11	cyn-2	F44G4.5	msd-2	T20H4.2	ZK1307.3 /// ZK1307.4
C01F6.2	C48B6.3	cyp-29A3	F47B3.2 /// F47B3.7	msp-45	T22B3.3	ZK262.4
C01G12.3	C50D2.3	D1081.4	F47B3.7	msp-49	T23G11.1	ZK354.7
C02F5.5	C54D10.10	D1081.5	F47D12.7	ncx-1	T27A3.5	ZK418.2
C03C10.2	C54G4.3	D2062.6	F49C12.15	nspb-3 /// nspb-2	T27E7.1	ZK484.5
C04F12.7	C55A6.4	D2062.7	F52H3.6 /// ZK938.1	poml-1	T28H11.7 /// ZC477.7	ZK484.7
C04G2.2	C55B7.3	decr-1.1	F53B6.4	ptr-14	tag-290	ZK550.5
C05C10.3	C55C2.4	decr-1.2	F54C1.8	R07E5.15	toh-1	ZK637.12
C05C12.5	C55C3.4	decr-1.3	F55F8.7	R08A2.1	tsp-19	ZK84.2
C06A8.6	catp-4	dlc-6	F55H12.5	R08A2.2 /// R08C7.8	ttr-27	ZK858.2
C08F11.10	clec-151	dpy-9	F56B3.6	R09E10.6	vha-14	
C08F8.6	col-10	E04F6.6	F56D6.13	R102.8	W01B6.6	
C09D4.3	col-103	F02E9.3	F58A6.9	R10D12.10	W01D2.3	
C09H5.7	col-110	F07A5.2	F58D5.7	R10E9.2	W03D8.10 /// W03D8.9	
C10G11.8	col-117 /// col-3	F07F6.1	F58E6.5	rmd-3	W03D8.3	
C10H11.7	col-12	F09C12.8	F58H1.6	rmd-4 /// rmd-6	W09D6.4 /// Y47D3A.13	
C12D8.9	col-126 /// 127	F10D11.3	F59E11.5	rnh-1.1	Y106G6D.3	
C14C10.1	col-129	F11G11.4	fbxa-196	rol-1	Y18D10A.21	
C14C11.1 /// C18G1.9 ///ZC317.6	col-133	F13A7.1	fis-1	scrm-7	Y23H5B.2 /// Y39G10AR.16	
C14H10.1	col-137	F17C8.7 /// T23F11.2	gipc-1	smz-2	Y37F4.5	
C15C6.2	col-141	F17E9.5	gipc-2	snb-5	Y38C1AA.7	
C15C7.6	col-142	F25B3.4	gska-3	snf-5	Y38E10A.17	
C16C8.18	col-146	F27C8.5	gsp-3	spe-11	Y39G10AR.16	
C16D9.5	col-147	F31E8.5	H06A10.1	spe-46	Y43C5B.2	
C17H12.3	col-149	F32B6.4	H32C10.1	ssp-31	Y44A6D.5	
C18B12.2	col-150	F33D11.2	htas-1	ssp-33	Y47D7A.13	
C24A11.1	col-161 /// 162	F33D11.7	irld-14	ssq-1	Y49G5A.1	
C25A8.2 /// R02D5.7	col-164	F35C11.3 /// M05D6.1	K01H12.4	ssq-3	Y51B9A.5	
C26C6.6	col-167 ///168 ///170	F36A2.11	K04G2.4	sss-1	Y53F4B.19	
C27D6.3	col-17	F36A2.14	K05F1.9	sss-2	Y54E2A.9	

Supplemental Table S4: The genes whose expression was upregulated throughout the time course (Total 25 genes)

Gene symbols
acbp-3
C17F4.7
C31C9.2
C49A9.2
C52B11.5
C55A1.6
F22H10.3
F47D12.6
fard-1
fbxc-1-5
his-41
hmgs-1
K07C11.7
K08B12.3
kel-8
mboa-3
perm-2
T21F4.1
T28C6.8
W01C8.5
Y102A11A.3
Y94H6A.12 /// Y94H6A.7
ZK1307.1
ZK813.3
ZK856.5

Supplemental Table S5: The genes whose expression was upregulated during starvation and downregulated during refeeding (Total 284 genes)

Gene symbols							
aakb-1	cki-1	F36F2.2	hrg-1	nhr-117	sek-4	twk-7	ZK813.2
aakg-4	clec-222	F41E6.5	icl-1	nhr-120	siah-1	ugt-54	ZK813.7
aex-6	clec-54	F41G4.8	ikb-1	nhr-122	skr-19	vab-9	ZK899.1
apm-1	cnb-1	F42C5.9	ist-1	nhr-133	snb-2	VC5.2	ZK971.1
B0310.3	coel-1	F43C11.7	K01A2.10	nhr-143	sodh-1	W02C12.2	
B0546.4	comt-4	F43G6.8	K02A6.3	nhr-144	sox-2	W02D7.8	
best-13	crm-1	F45D3.4	K03A11.5	nhr-162	spp-10	W03F11.1	
btb-21	cutl-16	F46C3.2	K03E6.7	nhr-163	spp-8	W08A12.2	
btb-9	cyp-13A5	F46G10.2	K03H1.5	nhr-170	sqst-1	W09G12.7	
C06G8.3	cyp-32A1	F47B8.4	K04F10.1	nhr-18	sqst-2	Y105C5A.24	
C07A4.3	cyp-32B1	F48E3.8	K07A1.3	nhr-206	srr-6	Y105E8B.7	
C09D4.1	cyp-33C4	F52G2.3	K07A1.6/// R10H1.1	nhr-21	sta-1	Y22D7AR.6	
C09D4.2	D1046.5	F53A9.6	K10D11.2	nhr-211	sto-1	Y34F4.1	
C13C4.6	D1086.1	F53A9.8	K11E4.2	nhr-212	svh-2	Y37D8A.16	
C14B1.3	D1086.2	F53B2.8	kgb-2	nhr-232	T01B10.5	Y37D8A.19	
C15B12.1	dao-2 /// M03A1.8	F53E10.1	kpc-1	nhr-32	T01E8.1	Y40B10A.9	
C17H11.6	dct-1	F53F1.6	lact-2	nhr-36	T02E9.5	Y42G9A.1	
C18C4.5	dgk-2	F53F10.1	lec-7	nhr-40	T04F8.7	Y43C5A.3	
C18D4.8	DH11.2	F58D5.2	lgg-2	nhr-50	T05A8.3	Y43E12A.2	
C26E1.2	dhhc-10	F59B2.13	lim-9	nhr-59	T12A7.6	Y43F8B.9	
C29F7.1	dur-1	F59F5.3	M01A8.1	nhr-64	T12B3.3	Y46G5A.15	
C29F9.1	elt-7	fbxa-115	M01B2.10	nhr-79	T16G1.4	Y48A6B.7	
C32D5.12	ets-4	fbxa-156	M01H9.4	nhr-8	T18D3.7	Y51A2D.13	
C32F10.4	F02E9.5	fbxa-162	M163.1	nhr-90	T18D3.8	Y51B9A.9	
C33H5.13	F08G2.4	flp-10	M57.1	nhr-99	T19C3.4	Y53H1B.2	
C34C6.7	F08H9.3	fmi-1	M60.4	nlp-39	T19C4.5	Y54G2A.11	
C34D1.4	F10G8.8	fmo-1	maa-1	npr-28	T19H5.4	Y57A10A.14	
C34D10.2	F13E9.11	fmo-2	mab-31	pes-8	T27E4.7	Y57A10A.26	
C35C5.8	F16C3.2	frpr-11	madf-10	pitr-3	T28B8.1	Y57G11B.2	
C38C3.4	F17C11.4	ftn-1	math-20	plc-2	tag-234	Y62H9A.3	
C41G11.1	F17H10.3	glo-4	memo-1	psd-1	tag-243	Y6E2A.4	
C44H9.6	F18C5.10	glt-5	mfb-1	R06C1.6	tag-304	Y77E11A.14	
C52E2.4	F18G5.6	gpa-9	mpk-2	R07G3.8	tps-1	Y7A5A.6	
C53B7.2	F20C5.6	gst-22	nape-1	ras-1	tre-3	ZC373.2	
C53B7.3	F21A3.2	gsto-3	ncr-1	rfs-1	tre-5	ZC443.3	
cal-2	F21D5.3	H06I04.6	nfm-1	rhi-1	trx-3 (thioredoxin)	zig-7	
ceh-20	F21F8.2	H19N07.3	nhl-3	ric-19	tth-1	ZK1240.3	
cex-2	F26F12.3	H20E11.3	nhr-101	rig-1	ttr-26	ZK287.3	
chil-23	F34H10.3	hex-1	nhr-109	rsd-3	ttr-37	ZK673.2	
cht-3	F35D11.3	hil-1	nhr-110	sax-1	ttr-5	ZK813.1	

Supplemental Table S6: The genes whose expression was upregulated during starvation and downregulated during refeeding (Total 124 genes)

Gene symbol			
aat-4	F21A3.3	oat-1	Y11D7A.3
acl-1	F22E5.1	pcp-2	Y26D4A.21
acl-4	F31D4.8	pcyt-1	Y39D8A.1
acly-1	F41E6.12	pes-9	Y41C4A.12
acs-1	F43C9.1	pho-13	Y43F4B.5
amt-4	F49C12.14	ppat-1	Y49E10.18
B0041.5	F53H8.3	R07E3.4	Y4C6B.5
bas-1	F55B11.1	scav-4	Y50D4B.3 /// Y50D4B.4
best-26	F58F9.4	sir-2.3	Y51A2D.18
C01G10.9	F58G6.3 /// F58G6.7	slc-17.5	Y53G8AR.7
C35A11.4	fbp-1	smd-1	Y54G11A.7
C42D4.2	fmo-3	spp-23	Y54G9A.4
C44C1.5	gale-1	sptl-2	Y73B6BL.14
C45E5.1	gln-1	sri-24	zip-3
C49A9.4	gly-8	sur-5	ZK185.3
C50A2.3	gpdh-1	T01H8.2	ZK228.3
C52D10.1	gst-26	T07A5.1	ZK228.4
cah-5	haao-1	T07E3.4	ZK512.7
cbl-1	hgo-1	T10G3.3	ZK6.11
cbs-1	hpd-1	T11G6.4	
cdf-1	hprt-1	T22H2.4	
cima-1	hrg-4	T23F11.2	
ckc-1	hsp-16.1 /// hsp-16.11	T25B9.1	
coq-1	hsp-16.2	T25B9.9	
cyp-36A1	hsp-16.41	T28D6.3	
cysl-2	hsp-16.48 /// hsp-16.49	tdo-2	
cysl-3	hsp-17	trap-2	
D1025.2	K05C4.2	ugt-21	
D2005.6	K05F1.10	ugt-22	
daf-36	K11C4.2	ugt-46	
dnpp-1	K11H3.3	ugt-48	
elo-9	kin-15	ugt-63	
F09B12.3	laat-1	vha-12	
F09E5.3	M70.2	W09B6.5	
F19B2.5	nspe-3	Y105C5B.14	

Supplemental Table S7: The primer sequences used for qRTPCR

Genes	Primer names	Sequences
nhr-8	nhr-8F	5`-caaagactcaccgcatacga-3`
	nhr-8B	5`-acgccggagaaactgtgtag-3`
nhr-18	nhr-18F	5`-gatttgaatcccagcagcat-3`
	nhr-18B	5`-cctccaacgtccttgtgatt-3`
nhr-21	nhr-21F	5`-tgctcagtttcctcatcgtg-3`
	nhr-21B	5`-ggcgtcagttttgttcgatt-3`
nhr-32	nhr-32F	5`-cattacggtgttcgatcgtg-3`
	nhr-32B	5`-acgatccattggaggtatgc-3`
nhr-36	nhr-36F	5`-attggctcggtttcgatatg-3`
	nhr-36B	5`-atgttcaccctggcctactg-3`
nhr-40	nhr-40F	5`-ggaatggtttgtgcgagatt-3`
	nhr-40B	5`-cgtacaatccggttcgagtt-3`
nhr-50	nhr-50F	5`-aatgtcagccaaagcgaaat-3`
	nhr-50B	5`-tcggaagaaagtttcggaga-3`
nhr-59	nhr-59F	5`-gaacatttggaatcggttgg-3`
	nhr-59B	5`-tgaagtcgatgattgcttgc-3`
nhr-64	nhr-64F	5`-tcagatgcagtttggctgac-3`
	nhr-64B	5`-gagaatccgttgtcgcattt-3`
nhr-79	nhr-79F	5`-aatgccaaaaagcaggaatg-3`
	nhr-79B	5`-agctcatcgattcccatcag-3`
nhr-90	nhr-90F	5`-cgtttttgcaagactcgaca-3`
	nhr-90B	5`-gaagatccaatgcggtgatt-3`
nhr-99	nhr-99F	5`-cccacgcttcaagtcaaaat-3`
	nhr-99B	5`-ttgggttcctcatcttcctg-3`
nhr-101	nhr-101F	5`-agaagccctgtgctcaaaaa-3`
	nhr-101B	5`-cagccttgtcagcatacgaa-3`
nhr-109	nhr-109F	5`-atctttgctcctcccaatga-3`
	nhr-109B	5`-actgatggctctgcgacttt-3`
nhr-110	nhr-110F	5`-gggacgtcttgaaggcatag-3`
	nhr-110B	5`-tttcacggggaatgaaaaag-3`
nhr-117	nhr-117F	5`-caggatcttgtcagccgaat-3`
	nhr-117B	5`-acgtgtcgttggtttgatga-3`
nhr-120	nhr-120F	5`-aatggctgacgtcgagaaag-3`
	nhr-120B	5`-ttgtaagacgttcccgctct-3`
nhr-122	nhr-122F	5`-cctgcagaaatggtcgaaat-3`
	nhr-122B	5`-tcaacgctgatagtgccaag-3`
nhr-133	nhr-133F	5`-acgtctggtggatgtgacaa-3`
	nhr-133B	5`-gctcccaaaaatgattgctc-3`

nhr-143	nhr-143F	5`-caaaaactgccacatggttg-3`
	nhr-143B	5`-cgaaaccacaaattgccttt-3`
nhr-144	nhr-144F	5`-cggcacagatgagggttaat-3`
	nhr-144B	5`-cgacaattccccacacaata-3`
nhr-162	nhr-162F	5`-atcatcgatgccgaaagaaa-3`
	nhr-162B	5`-gttgaaccacttttcgcaca-3`
nhr-163	nhr-163F	5`-cattatggtggcagttgctg-3`
	nhr-163B	5`-cgtggtggttgaacttgtgt-3`
nhr-170	nhr-170F	5`-ccagttctcaatcccctcaa-3`
	nhr-170B	5`-ttgtgcaatgagtgggacat-3`
nhr-206	nhr-206F	5`-tgcaaagcatttttcagacg-3`
	nhr-206B	5`-cgtggttctcttttcgcaat-3`
nhr-211	nhr-211F	5`-gccggatgaggaaatgttta-3`
	nhr-211B	5`-tgagctccgtgctttaggat-3`
nhr-212	nhr-212F	5`-tgtgccaatgagtttttgga-3`
	nhr-212B	5`-agctcaaccatttcgctgtt-3`
nhr-232	nhr-232F	5`-aagtccgtgtggaaaattgc-3`
	nhr-232B	5`-caggaaagctgagccaacat-3`
ama-1	ama-1F	5`-ggacgacgtgttcctacgat-3`
	ama-1B	5`-aacgcggtaccatcagtttc-3`
inf-1	inf-1F	5`-cgtgcaagctctcgttatgg-3`
	inf-1B	5`-gagggcgctcatgacctt-3`
daf-12	daf-12 F	5`-tgcacaacggcgaaaaa-3`
	daf-12 B	5`-ggcacgatgagccataatct-3`
daf-16	daf-16 F	5`-caggagtcgaagccgattaa-3`
	daf-16 B	5`-gacactgttcaactcgtggtatga-3`
